# Position-Dependent Stability of an Unconstrained Total Talar Replacement and Its Suture Reinforcement: A Human Cadaveric Weight-Bearing Computed Tomography Study

**DOI:** 10.3390/bioengineering13090990

**Published:** 2026-08-27

**Authors:** Georgi Raykov, Preslav Penev, Dominic Gehweiler, Jan Barcik, Boyko Gueorguiev, Hans-Christoph Pape, R. Geoff Richards, Dimitar Raykov, Torsten Pastor

**Affiliations:** 1 Clinic of Orthopaedics and Traumatology, Sonnenhofspital, 3006 Bern, Switzerland; georgidraykov@gmail.com; 2 Medical Faculty, University of Zurich, 8052 Zurich, Switzerland; torsten.pastor@luks.ch; 3 AO Research Institute Davos, 7270 Davos, Switzerland; dominic.gehweiler@aofoundation.org (D.G.); jan.barcik@aofoundation.org (J.B.); geoff.richards@aofoundation.org (R.G.R.); 4 Department of Orthopaedics and Traumatology, Medical University of Varna, 9002 Varna, Bulgaria; dr_penev@abv.bg (P.P.); dimitar.raykov@proton.me (D.R.); 5 Department of Traumatology, University Hospital Zurich, 8091 Zurich, Switzerland; hans-christoph.pape@usz.ch; 6 Department of Orthopaedic and Trauma Surgery, Lucerne Cantonal Hospital, 6004 Lucerne, Switzerland

**Keywords:** talus, total talar replacement, talar collapse, ankle stability, weight-bearing CT, suture/ligament reconstruction, cadaveric biomechanics, 3D-printing

## Abstract

Total talar replacement (TTR) is a joint-preserving alternative to arthrodesis in end-stage talar collapse. However, excising the talus detaches its anchoring ligaments and capsule, and unconstrained prosthesis constructs are prone to malalignment and dislocation. Moreover, it remains unclear whether the instability and its suture stabilisation vary across foot positions. Therefore, the aim of this study was to quantify the positional, load-dependent stability of an unconstrained TTR and the effect of its suture stabilisation. Five human cadaveric lower legs were imaged by weight-bearing computed tomography (CT) imaging in nine positions under 10 N and 700 N load in the intact condition and then with an unconstrained custom 3D-printed specimen-specific TTR, followed by supplementary transosseous suture stabilisation of the anterior talofibular ligament (ATFL) and deep tibiotalar deltoid in the third condition. In the intact ankle condition, load-induced displacements were minimal and unaffected by foot position, averaging no more than 0.52 mm and 0.62°. The unconstrained TTR exhibited position-dependent load-induced displacements, with talar shift increasing significantly by 2.29 mm in inversion and calcaneofibular distance decreasing significantly by 1.54 mm in eversion compared with the intact ankle condition (*p* ≤ 0.033). Suture stabilisation significantly decreased talar shift by 1.45 mm in inversion and significantly increased calcaneofibular distance by 1.37 mm in eversion, restoring these load-induced displacements toward the intact ankle condition (*p* = 0.002). In this human cadaveric weight-bearing CT imaging study, an unconstrained TTR benefited from suture reconstruction of the ATFL and deep tibiotalar deltoid attachments, highlighting the value of evaluating construct stability under load across the range of foot positions rather than in neutral stance alone.

## 1. Introduction

The stability of a total talar replacement (TTR) across the range of foot positions during ankle motion is poorly understood. Excision of the talus removes the capsuloligamentous attachments anchoring it within the mortise, and both existing human cadaveric and clinical studies have associated unconstrained prosthesis constructs to persistent malalignment and dislocation. However, these investigations have been confined to a statically loaded foot in neutral position, and none of them have assessed whether the prosthesis instability varies across the range of foot positions, or whether supplementary suture stabilisation restrains it in positions challenging reconstructed restraints.

The talus is anatomically predisposed to osteonecrosis—roughly 60% of its surface is articular, with sparse soft-tissue attachments, and its blood supply is retrograde and easily disrupted by trauma [1,2]. Regardless of etiology, progressive collapse of the talar dome results in pain, loss of hindfoot alignment and considerable functional impairment [3]. The traditional salvage for end-stage collapse—tibiotalocalcaneal arthrodesis—reliably relieves pain; however, it eliminates ankle and subtalar motion and carries substantial nonunion rates [4], while talectomy is complicated by leg-length discrepancy and fixed deformity. Custom TTR has therefore been proposed as a joint-preserving alternative, and modern patient-specific implants have exhibited promising clinical results, durable in large and long-term series [5,6], and satisfactory in the short term in small cohorts [7]. Among the described configurations, the unconstrained prosthesis construct preserves motion at both tibiotalar and subtalar joints and is most attractive when the surrounding cartilage is intact. It correspondingly requires the most from the ligamentous and capsular envelope for its stability.

Biomechanical stability is the central concern of the unconstrained prosthesis construct. Implantation necessarily disrupts the ligaments anchoring the talus within the mortise, and cadaveric testing has shown that the insertion increases anterior drawer translation and talar tilt relative to an intact ankle, with dislocation when downsized prostheses are used [8]. Hindfoot malalignment after TTR has likewise been reported clinically. In response, stabilisation strategies have been proposed, including an internally braced prosthesis whose suture limbs reconstruct the anterior talofibular ligament (ATFL), the deep tibiotalar deltoid and the interosseous talocalcaneal ligaments [9], as well as designs allowing reattachment of the native collateral ligaments through the implant [10]. Small clinical series have investigated TTR configurations with and without collateral-ligament reconstruction [11]. To date, however, no controlled human cadaveric study has quantified the effect of such stabilisation on prosthesis construct stability across the range of foot positions during ankle motion.

This gap matters because ankle and hindfoot stability is inherently positional. The use of sutures reconstructs talar attachments of two restraints, each one recruited in only parts of foot positions, namely the ATFL, being tensioned in plantarflexion and inversion, and the deep tibiotalar deltoid, resisting lateral translation of the talus. Much of the inversion restraint of a neutral and dorsiflexed foot is carried by the calcaneofibular ligament, whereas the hindfoot eversion restraint is mainly provided by the superficial deltoid, neither of which is reconstructed by the proposed suture stabilisation strategies [12,13]. That is why a prosthesis that appears stable in neutral stance may therefore displace when the hindfoot is driven into positions that challenge each replaced restraint, because a suture construct may exert its benefit only in specific positions. Prior biomechanical work has generally examined the construct in a neutral position only; however, the behaviour of the unconstrained and suture-stabilised prosthesis construct across the range of foot positions, as well as under physiological loading, remains unclear.

Therefore, the aim of this human cadaveric study was to quantify the positional, load-dependent stability of an unconstrained TTR and the effect of its supplementary transosseous suture stabilisation by means of weight-bearing computed tomography (CT) imaging. It was hypothesized that (1) the unconstrained TTR would demonstrate position-dependent instability, greatest in the positions that challenge the native ATFL and deep tibiotalar deltoid restraints, and that (2) the suture stabilisation would reduce the load-induced displacement of the prosthesis, most markedly in these same positions.

## 2. Materials and Methods

### 2.1. Specimens and Preparation

Five fresh-frozen human cadaveric lower legs (three right and two left specimens from three female and two male donors; age: 72.2 ± 5.7 years (mean value ± standard deviation), range: 65–81 years) were used. All donors provided their written informed consent inherent within the donation of the anatomical gift statement during their lifetime. The study was approved by the internal review board of the AO Research Institute Davos. Each specimen was stored at −20 °C and screened by CT (Revolution EVO, GE Medical Systems (Schweiz) AG, Glattbrugg, Switzerland; 0.63 mm slices) to exclude previous surgery, fracture or foot and ankle deformity. The specimens were thawed at room temperature for 24 h before preparation.

The legs were transected through the middle third of the calf, approximately 10 cm proximally to the ankle joint. The proximal tibia and fibula were embedded in polymethylmethacrylate (PMMA; SCS Beracryl D-28, Suter Kunststoffe AG, Fraubrunnen, Switzerland) with the syndesmotic ligaments and interosseous membrane preserved.

### 2.2. Surgical Conditions

**Figure 1:**
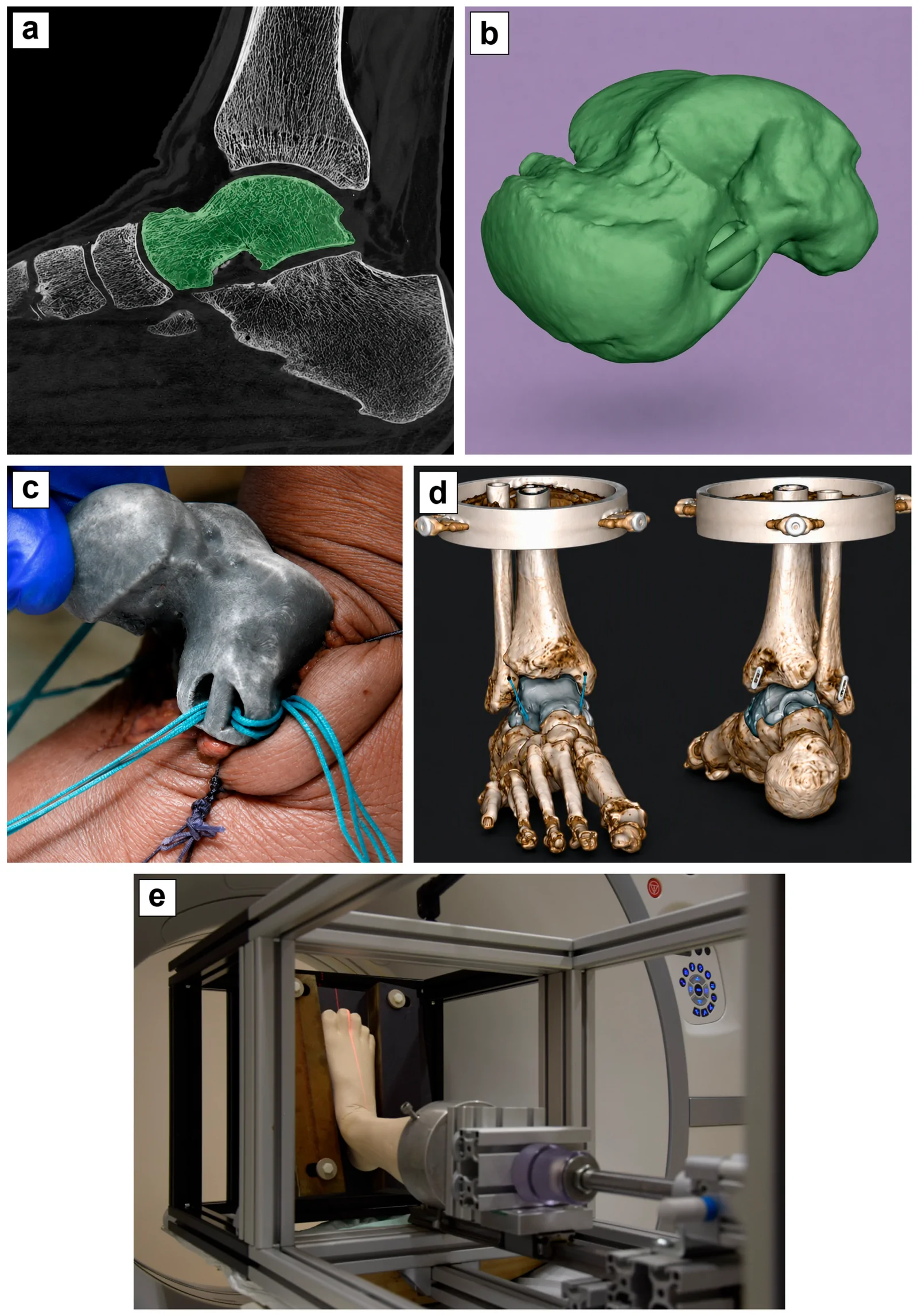
TTR fabrication, surgical construct and loading setup. (**a**) Segmentation of the native talus from a CT scan used to design the custom specimen-specific TTR. (**b**) Reconstructed 3D talar model. (**c**) Custom 3D-printed specimen-specific TTR with a supplementary transosseous suture construct, whose limbs replace ATFL and deep tibiotalar deltoid restraints through eyelets on the medial and lateral aspects of the prosthesis. (**d**) 3D weight-bearing CT rendering of the foot in the loading ring in anterior (**left**) and posterior (**right**) views. (**e**) Specimen mounted in a custom radiolucent, pneumatically actuated loading frame within the CT gantry for weight-bearing acquisition.

Each specimen served as its own control across three sequential surgical conditions with the following: (1) intact native talus (Intact-Native); (2) an unconstrained custom three-dimensionally (3D)-printed specimen-specific TTR (TTR-Alone); and (3) the same unconstrained TTR plus supplementary transosseous suture stabilisation (TTR-Suture). The sequential order was fixed because each condition resulted from an irreversible surgical step: the resected talus could not be reimplanted, and suture removal would leave bone tunnels that would alter the TTR-Alone condition. Consequently, counterbalancing the sequence was not feasible. The prosthesis was inserted through an anterior ankle approach. Following capsulotomy, the native talus was excised while preserving the malleoli. The prosthesis was implanted using a press-fit technique without bone cement. The suture construct implemented two limbs—a lateral and a medial one—and was fixed using two two-hole 12 mm suture buttons (BicepsButton, 2.6 × 12 mm, Arthrex, Naples, FL, USA) made of titanium. The buttons were placed posteriorly at the medial and lateral malleoli, following drilling of the corresponding 3.0 mm tunnels in anteroposterior direction and using the same anterior ankle approach with two posterior incisions. No. 2 FiberWire (Arthrex) sutures—one strand per limb—were attached to the eyelets on both sides of the prosthesis, functionally substituting the talar attachments of the ATFL laterally and the deep tibiotalar deltoid ligament medially. Each limb was manually tensioned to maximum tension by a single surgeon with the foot held in a neutral position under 10 N preload and tied posteriorly over the button with a surgeon’s knot backed by five alternating half-hitches [14]. No tensiometer was used. FiberWire suture was chosen because of its high tensile strength and established use in ankle ligament reconstruction. Only the ATFL and the deep tibiotalar deltoid were reconstructed, being the two restraints most relevant to coronal-plane stability of the mortise. Additional limbs would have extended the exposure. The TTR and the suture construct are presented in Figure 1a–c.

The specimen-specific TTRs were fabricated by polyamide fused-filament additive manufacturing following CT segmentation of the intact tali. They were coated with a thin radiopaque layer to improve CT visibility without altering the geometry; a comparable radiopaque surface coating of a plastic talar prototype has been described by Sato et al. [8].

### 2.3. Weight-Bearing CT Imaging

Each specimen was mounted in a custom radiolucent, pneumatically actuated loading frame designed for axial loading of human cadaveric lower legs during CT acquisition (Figure 1d,e) [15,16]. For each separate surgical condition, weight-bearing CT scans were acquired in nine standardized foot positions: neutral (0°), plantarflexion (PF, 15°), dorsiflexion (DF, 10°), eversion (EV, 5°), inversion (IN, 5°), and combined foot positions in PF-eversion, DF-eversion, PF-inversion and DF-inversion. Each position was set using a custom radiolucent jig with angle-specific stops, locked before acquisition. Reproduction of the target angles was confirmed on the scout images with an accuracy of within ±1°. Every foot position was imaged under two loading scenarios: in unloaded reference state under 10 N axial preload and in loaded state under physiological axial load of 700 N approximating 70 kg full body weight of an average-weight adult in single-leg stance. Gait-level loads exceeding full body weight were not considered in order to avoid cumulative soft-tissue damage over the repeated acquisitions. The load was ramped once to the target value and held constant during acquisition. No preconditioning cycles were implemented. As a result, each specimen underwent 54 acquisitions (three surgical conditions with nine foot positions and two loading scenarios each), yielding 270 datasets across the specimens.

### 2.4. Measurements

Five parameters of interest were defined and evaluated during the measurements in a common reference coordinate system, following previously published validated weight-bearing CT definitions: two primary parameters—(1) talar shift (TAS) and (2) calcaneofibular distance (CFD); two parameters characterising coronal-alignment—(3) hindfoot alignment angle (HAA) and (4) subtalar vertical angle (SVA); and one parameter characterising the global-alignment in neutral foot position—(5) foot-ankle offset (FAO). TAS was defined as the coronal-plane mediolateral distance between the centre of the talar dome and the tibial anatomical axis [17], CFD was defined as the shortest coronal-plane distance between the lateral calcaneal wall and the tip of the lateral malleolus [18,19,20], HAA was defined as the coronal-plane angle between the tibial anatomical axis and the hindfoot axis connecting the talar-dome centre to the most plantar calcaneal point [17], SVA was defined as the coronal-plane varus/valgus inclination of the posterior subtalar facet relative to the tibial vertical axis [21,22], and FAO was defined as the perpendicular offset of the talar dome centre from the medial forefoot, the lateral forefoot and the weight-bearing aspect of the calcaneus (constituting a weight-bearing tripod), expressed as a percentage of foot length [23,24]. The latter parameter was evaluated only in neutral stance because the weight-bearing tripod is a physiologically valid reference only in this foot position.

All measurements were performed in 3D Slicer (version 5.6.2) [25] following a standardised workflow applied identically to every dataset. Each weight-bearing CT volume was first reoriented into the common reference coordinate system, with the longitudinal axis of the tibia aligned vertically and the sagittal plane oriented parallel to the line connecting the centre of the calcaneus and the shaft of the second metatarsal [17,21]. All measurements were performed in this tibia-referenced coordinate system.

**Figure 2:**
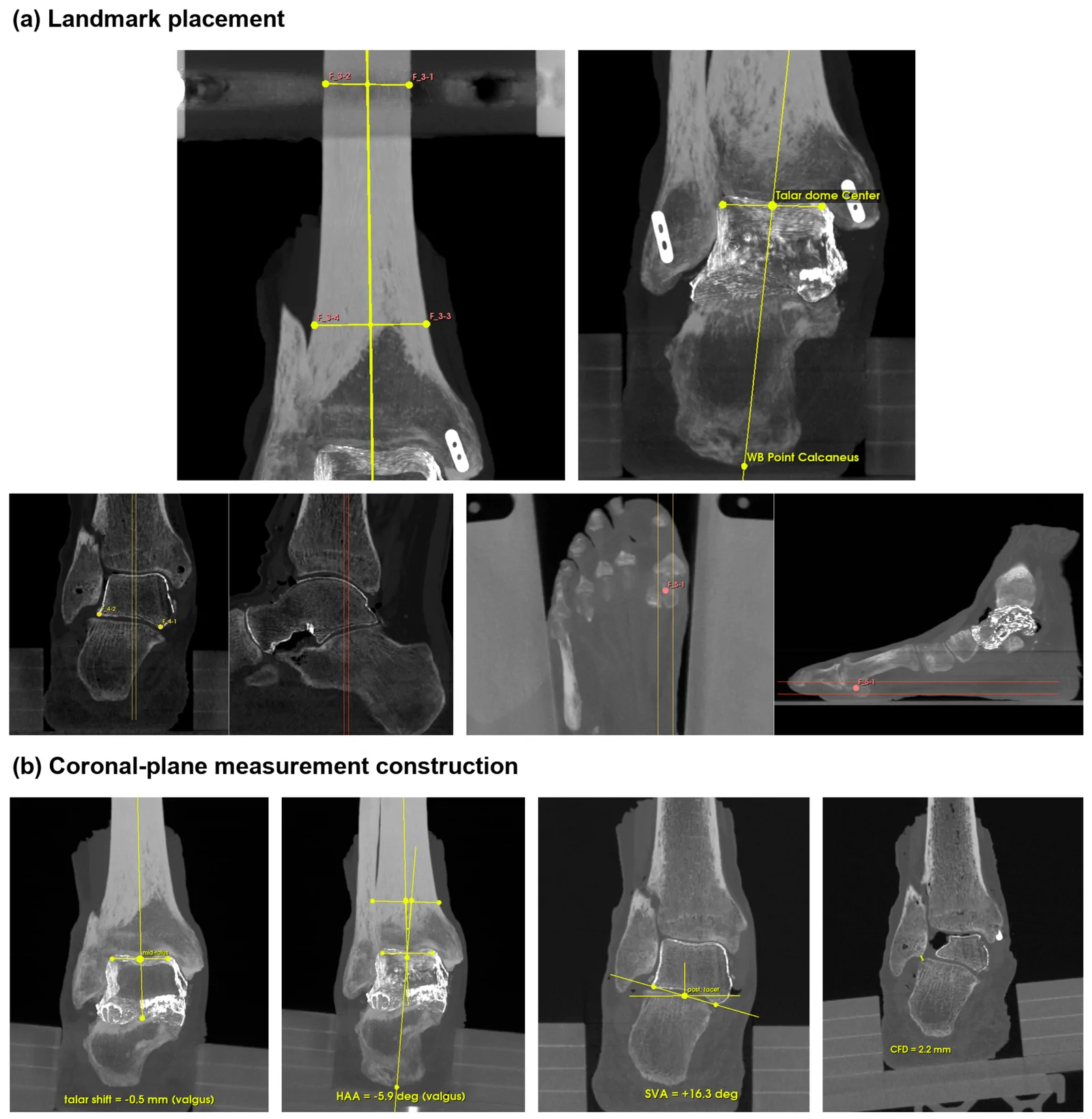
Visualisation of the landmarks and axes used for evaluation of the parameters of interest. (**a**) Landmark placement for determination of the tibial anatomical axis, talocalcaneal axis, posterior subtalar facet and first metatarsal head. (**b**) Coronal-plane view used for evaluation of the four coronal-plane parameters talar shift (TAS), hindfoot alignment angle (HAA), subtalar vertical angle (SVA) and calcaneofibular distance (CFD).

A single-shared set of eleven anatomical landmarks was placed on all reconstructions in the common reference coordinate system (Figure 2) consisting of the following: (1–4) four tibial cortical landmarks at two levels, defining the tibial anatomical axis and the mediolateral reference (Figure 2a, top left); (5, 6) the medial and lateral edges of the most central and proximal part of the talar dome (Figure 2a, top right); (7, 8) the medial and lateral edges of the posterior talar facet of the subtalar joint visualised on the middle coronal plane of the joint (Figure 2a, bottom left); (9) the most plantar weight-bearing point of the calcaneus (Figure 2a, top right); (10) the most lateral point of the calcaneal wall at the level of the fibular tip; and (11) the tip of the lateral malleolus. For each landmark, the most prominent bony aspect was first located on a thick (30 mm) maximum-intensity slab and then set on the thin reconstruction, with its foot position confirmed in all three orthogonal planes (axial, coronal and sagittal) before acceptance to ensure correct 3D localisation. The centre of the talar dome was defined as the midpoint of its medial and lateral edges (two-point method) to improve reproducibility. The mediolateral reference direction was derived from the medial-to-lateral labelling of the paired landmarks, resulting in foot-side independent evaluation of all parameters.

Landmark placement and evaluation of the parameters were performed independently by two observers who assessed the datasets in a randomised order, being masked to each other’s readings and to the automatically computed output. Each observer repeated the measurements three times for every CT scan. The average of the resulting six values was used for analysis.

### 2.5. Statistical Analysis

Statistical analysis was performed using R software package (version 4.6.1) and followed a paired within-specimen design since each specimen was tested in all surgical conditions, foot positions and loading scenarios. The dataset containing mean individual parameter values across observers, stratified by surgical condition, foot position and loading scenario, is provided in Supplementary Table S1, whereas the corresponding dataset of individual parameter values for each observer under all surgical conditions, foot positions and loading scenarios is presented in Supplementary Table S2. Normality of paired-differences’ distribution was screened with the Shapiro−Wilk test. Paired-Samples *t*-test was applied for parametric analysis. Due to the fact that it is not possible to identify *p*-values smaller than 0.062 at a sample size of five specimens with the Wilcoxon Signed-Rank test, an exact permutation test was used for non-parametric sensitivity analysis. The primary outcome was the load-induced change in each parameter—calculated from its value under 700 N load minus the value under 10 N load—within each surgical condition and foot position. Within-specimen paired differences were analysed in three pre-specified families: (A) effect of axial loading (load-induced change) within each surgical condition and foot position, (B) effect of surgical condition (TTR-Alone vs. Intact, TTR-Suture vs. Intact and TTR-Suture vs. TTR-Alone) on load-induced change within each foot position and (C) foot-position-specific effect of suture stabilisation in four pre-specified primary foot positions—inversion and PF-inversion for the ATFL restraint, and eversion and DF-eversion for the deep tibiotalar deltoid restraint. Multiplicity was controlled within each family with the Holm−Bonferroni procedure. Effect sizes were expressed as Cohen’s dz with a 95% Confidence Interval (95% CI) of the mean difference. Two-Way Repeated Measures Analysis of Variance (ANOVA) implementing surgical condition and foot position as factors and considering Greenhouse−Geisser correction was applied to evaluate load-induced changes and analyse whether the effect of surgical condition varied across foot positions. Inter- and intra-observer reliabilities were assessed using the Intraclass Correlation Coefficient (ICC) based on a two-way random-effects model with absolute agreement and single measures [26]. The general level of significance was set at 0.05 for all statistical tests. A paired study design with a sample size of five reaches 80% power at a level of significance of 0.05 only for a standardized effect size of dz not smaller than 1.68, so the study design is sensitive to large within-specimen effects and underpowered for small ones. That is why the non-significant off-target contrasts should be better interpreted as inconclusive than as evidence of no effect. The primary inference rests on the four pre-specified provoked contrasts in family C, corrected within this four-position family and defined before data collection, along with a nine-position correction applied for conservative sensitivity analysis over the full foot-position set in family B.

## 3. Results

### 3.1. Reliability and Distributional Assumptions

Reliability was good to excellent for every parameter of interest. The ICC ranged between observers from 0.89 (95% CI: 0.84–0.92) for SVA to 0.96 (95% CI: 0.94–0.97) for CFD. Within a single observer, the ICC ranged from 0.87 (95% CI: 0.85–0.90) to 0.92 (95% CI: 0.91–0.94) across all parameters. The results for measurement precision of the parameters—excluding FAO evaluated in the neutral position only—are presented in Table 1.

The load-induced changes in the pre-specified provoked foot positions inversion, eversion, PF-inversion and DF-eversion reported below were identified above the threshold of detectable change, whereas the remaining load-induced changes were detected close to the minimal detectable change.

The paired load-induced differences were normally distributed in 95% of the tests (Shapiro−Wilk test, *p* > 0.05), and the exact permutation test yielded results consistent with those of the Paired-Samples *t*-test for all primary contrasts. Consequently, parametric results are reported below.

### 3.2. Load-Induced Change (Primary Outcome)

**Figure 3:**
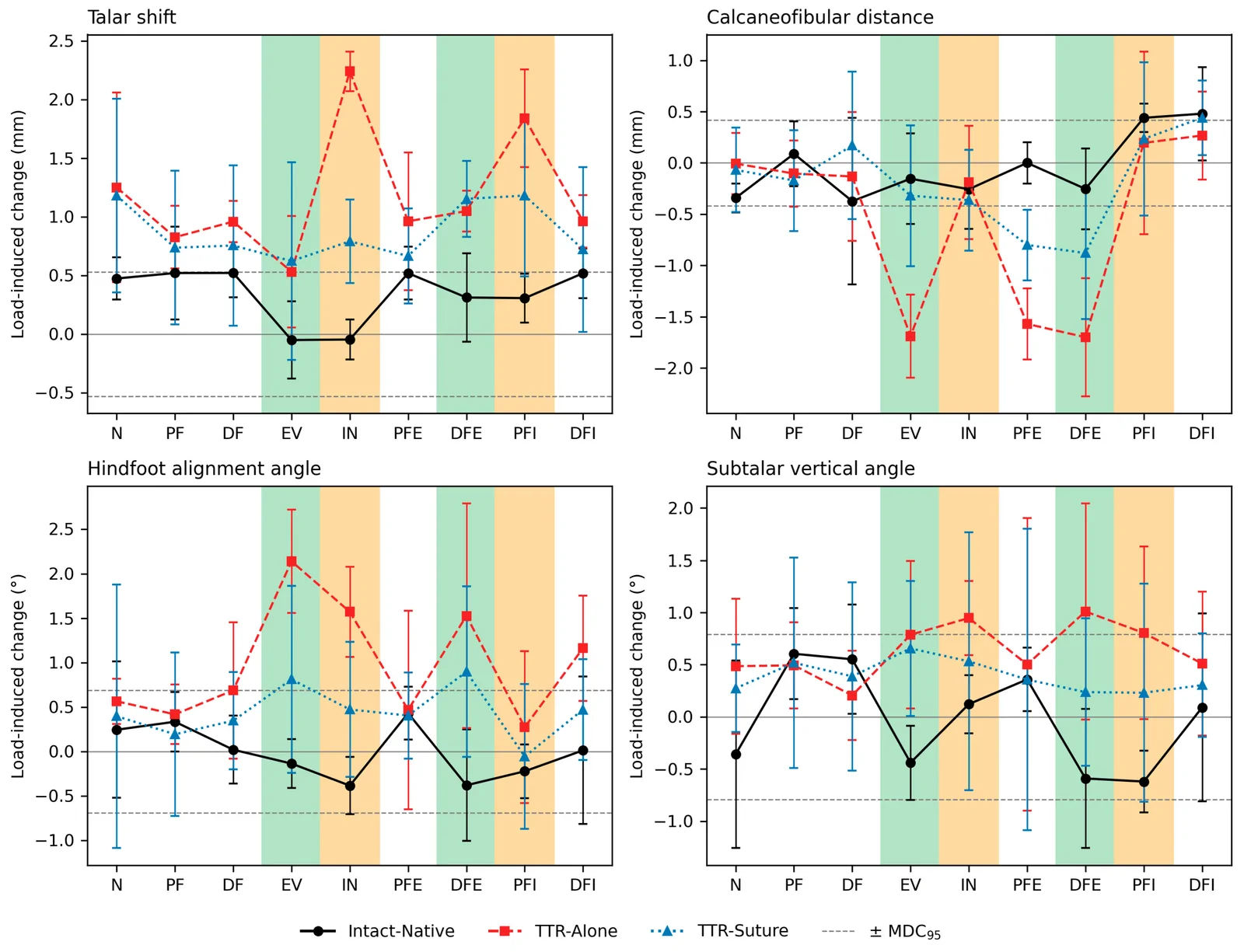
Load-induced change (value under 700 N load minus value under 10 N load) of the four coronal-plane parameters of interest talar shift (TAS), hindfoot alignment angle (HAA), calcaneofibular distance (CFD) and subtalar vertical angle (SVA) by surgical condition (Intact-Native, TTR-Alone and TTR-Suture), across the nine standardised foot positions neutral (N), plantarflexion (PF), dorsiflexion (DF), eversion (EV), inversion (IN), PF-eversion (PFE), DF-eversion (DFE), PF-inversion (PFI) and DF-inversion (DFI) in terms of the mean value and standard deviation. Shaded bands mark the pre-specified provoked positions (gold: IN and PFI challenging ATFL restraint; green: EVand DFE challenging deep tibiotalar deltoid restraint). Dashed lines mark the minimal detectable change of one measurement in one specimen at 95% confidence ([±] MDC_95_) for each parameter.

In Intact-Native surgical condition, the load-induced change of every parameter was small and did not vary with foot position: TAS changed by no more than 0.52 mm on average across the nine foot positions, HAA and SVA changed by no more than 0.62°, and CFD changed by no more than 0.48 mm (Figure 3). The native ligamentous envelope held the talus during loading.

In the TTR-Alone surgical condition, the unconstrained TTR displaced more and selectively. Load-induced TAS change rose from 1.25 mm on average in the neutral foot position to 1.84 mm in PF-inversion and 2.24 mm in inversion—the positions that would challenge the ATFL restraint. Expressed as the excess over the Intact-Native surgical condition, this load-induced change was 1.53 mm (95% CI: 0.88 to 2.18) greater in PF-inversion (dz +2.93) and 2.29 mm (95% CI: 1.91 to 2.66) greater in inversion (dz +7.51). The load-induced CFD change in the TTR-Alone surgical condition demonstrated a mean narrowing of 1.69 mm in eversion and 1.70 mm in DF-eversion—that would challenge the deep tibiotalar deltoid. This narrowing was 1.54 mm (95% CI: 0.82 to 2.26) and 1.45 mm (95% CI: 0.52 to 2.38) more in eversion and DF-eversion than in the Intact-Native surgical condition (dz: −2.65 and −1.93), respectively. Neutral stance remained comparatively stable with regard to all coronal-plane parameters.

In the TTR-Suture surgical condition, the suture-stabilised TTR displaced less than in TTR-Alone surgical condition (unconstrained TTR), and the decrease was mostly expressed in the provoked foot positions (inversion, eversion, PF-inversion and DF-eversion). The load-induced TAS change in inversion fell from 2.24 to 0.79 mm on average, and the load-induced CFD change (narrowing) in eversion fell from 1.69 to 0.32 mm on average, reducing the change toward the range in the Intact-Native surgical condition without fully restoring it (Figure 3).

### 3.3. Effect of Surgical Condition Under Physiological Load by Foot Position

**Figure 4:**
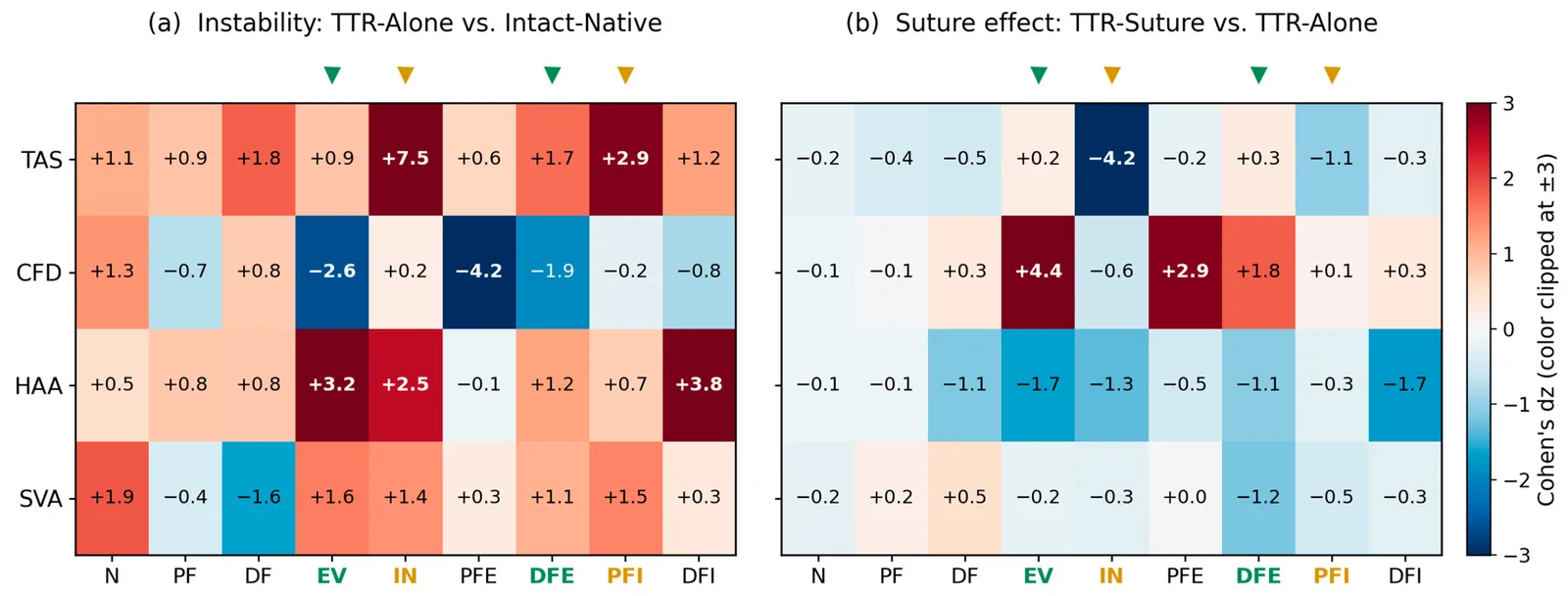
Rounded Cohen’s dz for every coronal-plane parameter of interest (rows: talar shift (TAS), calcaneofibular distance (CFD), hindfoot alignment angle (HAA) and subtalar vertical angle (SVA)) at every foot position (columns: neutral (N), plantarflexion (PF), dorsiflexion (DF), eversion (EV), inversion (IN), PF-eversion (PFE), DF-eversion (DFE), PF-inversion (PFI) and DF-inversion (DFI)). (**a**) Instability—value in TTR-Alone minus value in Intact-Native surgical condition; (**b**) Suture effect—value in TTR-Suture minus value in TTR-Alone surgical condition. Negative values for CFD denote narrowing of the subfibular space. Coloured markers above the columns mark the four provoked positions (gold: inversion (IN), PF-inversion (PFI); green: eversion (EV), DF-eversion (DFE)). Large values in non-provoked combined positions (e.g., PF-eversion (PFE)) are exploratory sensitivity findings.

In TTR-Alone vs. Intact-Native surgical conditions, the unconstrained TTR displaced relative to the intact talus in a foot-position-specific pattern (Table 2, Figure 3 and Figure 4a). The load-induced TAS change increased most in inversion (2.29 mm, dz: +7.51) and PF-inversion (1.53 mm, dz: +2.93), whereas the load-induced CFD change decreased (narrowed) selectively in eversion (1.54 mm, dz: −2.65) and DF-eversion (1.45 mm, dz: −1.93). The load-induced HAA change rose in the provoked positions significantly in inversion and eversion (*p* ≤ 0.035) and non-significantly in DF-eversion (*p* = 0.325). Neutral stance remained comparatively stable, with non-significant change in all parameters.

### 3.4. Foot-Position-Specific Effect of Suture Stabilisation

**Figure 5:**
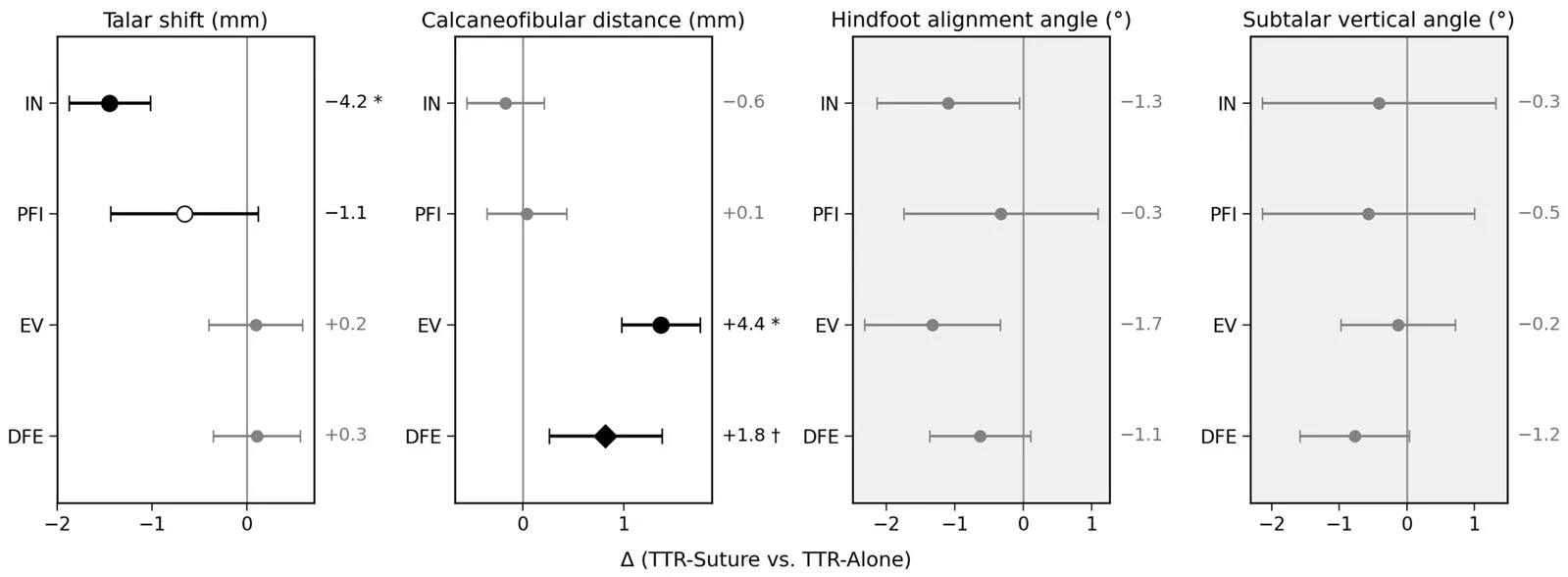
Effect of suture stabilisation: within-specimen difference in load-induced change between TTR-Suture and TTR-Alone surgical conditions (Δ) for each coronal-plane parameter (talar shift (TAS), calcaneofibular distance (CFD), hindfoot alignment (HAA) and subtalar vertical angle (SVA)) at the four pre-specified provoked positions (inversion (IN), PF-inversion (PFI), eversion (EV) and DF-eversion (DFE)). Markers represent mean paired differences; whiskers represent a 95% CI (unadjusted); rounded Cohen’s dz (dz) is annotated on the right hand side of each panel. Among the primary contrasts—TAS in inversion (IN) and PF-inversion (PFI), and CFD in eversion (EV) and DF-eversion (DFE) (first and second panels from left to right)—black filled circles and asterisks (*) indicate significance within both four- and nine-position sensitivity corrections (families C and B, respectively), filled diamond and dagger (†) indicate significance only within the four-position sensitivity correction (family C), and white circle indicates non-significance. Grey circles in all four panels are exploratory.

With regard to the ATFL restraint in the TTR-Suture surgical condition, the suture decreased the load-induced TAS change relative to the unconstrained TTR in TTR-Alone surgical condition by 1.45 mm (95% CI: 1.02 to 1.88) in inversion (four-position sensitivity-corrected *p* = 0.002, dz: −4.20) and by 0.66 mm (95% CI: −0.12 to 1.44) in PF-inversion—the latter not reaching significance (*p* = 0.079, dz: −1.05) (Figure 4b and Figure 5). With regard to the deep tibiotalar deltoid restraint, the suture in the TTR-Suture surgical condition increased the load-induced CFD change (narrowing) versus TTR-Alone surgical condition by 1.37 mm (95% CI: 0.98 to 1.76) in eversion (*p* = 0.002, dz: +4.38) and by 0.82 mm (95% CI: 0.26 to 1.38) in DF-eversion (*p* = 0.030, dz: +1.82). Under the nine-position sensitivity correction (family B), the inversion TAS and eversion CFD effects of suture stabilisation were significant (*p* ≤ 0.006), whereas the DF-eversion CFD and PF-inversion TAS effects of suture stabilisation remained non-significant (*p* ≥ 0.106). Therefore, the inversion TAS and eversion CFD effects can be considered robust within both four- and nine-position sensitivity corrections (families C and B, respectively). In contrast, the DF-eversion CFD and PF-inversion TAS effects should be regarded as correction-dependent or non-significant rather than established findings.

### 3.5. Surgical Condition by Foot-Position Interaction

The main effect of surgical condition (F) was significant for TAS (F = 11.32, *p* = 0.015), CFD (F = 16.62, *p* = 0.004) and HAA (F = 8.89, *p* = 0.016), with a borderline effect for SVA (F = 4.46, *p* = 0.051); generalized eta-squared (η^2^G) ranged from 0.28 to 0.56 (Table 3). Surgical condition by foot-position interaction was significant for TAS (F = 6.88, *p* = 0.007) and CFD (F = 5.34, *p* = 0.025), reached a trend for HAA (F = 3.82, *p* = 0.055), and was not significant for SVA (F = 1.30, *p* = 0.319). All *p*-values were Greenhouse−Geisser-corrected, with epsilon values for the interaction ranging from 0.15 to 0.19, so the interaction was tested with little residual power.

### 3.6. Foot Ankle Offset

The load-induced FAO change (evaluated in the neutral foot position only) remained essentially consistent across the three surgical conditions (Intact-Native: 9.3% (mean value), TTR-Alone: 9.9%, TTR-Suture: 8.2%; F = 0.94, *p* = 0.430). In all three surgical conditions, the load-induced FAO change exceeded the reported in vivo value of approximately 2.3% [23], most likely reflecting specimens’ mounting and embedding in the loading frame rather than a true clinical valgus phenotype. Accordingly, the most informative comparison is between the surgical conditions themselves, demonstrating no significant difference and indicating that the suture stabilisation had no measurable effect on FAO.

## 4. Discussion

This study quantified the positional, load-dependent stability of an unconstrained TTR and the effect of its supplementary transosseous suture stabilisation by means of CT imaging. Across the full range of foot positions, the unconstrained TTR was stable in neutral stance but displaced markedly when the foot was driven into positions challenging the native collateral ATFL and deep tibiotalar deltoid restraints. The suture stabilisation influenced the displacements selectively in the same positions, decreasing the talar shift in inversion and reopening the calcaneofibular distance in eversion toward the intact ankle state. The large effect size of these outcomes in the pre-defined provoked positions resulted from the small within-specimen variance and index reproducibility rather than clinical magnitude. As a result, these interpretable quantities exceeded the minimal detectable change in the corresponding measurements, reproducing biomechanical effects without fixing thresholds for symptoms, displacements or implant survival. The calcaneofibular narrowing was within the range for subfibular impingement evaluated from weight-bearing CTs in previous work [18,19,20]. As reported, just a few millimetres of coronal talar-component displacement or a few degrees of its tilt can result in edge-loading and subluxation of the talar dome [8]. On that scale, a load-induced talar shift excess of approximately 2.2 mm, decreased by approximately 1.4 mm with suture reinforcement, is within a range that may matter biomechanically, although no validated threshold ties talar-component displacement to symptoms, dislocation or implant survival. These values are not comparable to the sagittal manual anterior-drawer thresholds used for native lateral-ligament insufficiency (commonly > 3 to 5 mm), whose setting differs in plane, provocation, imaging modality and pathology, and for that reason, they are not used as a yardstick in the current study.

The rationale for examining the constructs across the range of foot positions—rather than in neutral stance only—follows directly from the anatomical configuration of the suture reinforcement. The talar attachments replacing the anterior talofibular ligament and the deep deltoid are recruited selectively—the former is challenged in inversion and plantarflexion-inversion, and the latter is loaded in eversion and dorsiflexion-eversion—so a prosthesis that is stable in the neutral position may displace only when driven into the positions that load each restraint, and a construct fixed at the lateral and medial malleoli should exert its effect there. A single-position assessment cannot capture this behaviour, which is the central methodological contribution of the present investigation.

Our approach extends the most directly comparable cadaveric work. Sato et al. quantified the consequences of unconstrained TTR under applied static stress and demonstrated anterior drawer instability, varus talar tilt and dislocation, and explicitly proposed future prosthesis designs incorporating eyelets for ligament reconstruction [8]. The suture-eyelet construct explored in the current study addressed this recommendation, and the positional, load-based protocol examined it under conditions closer to ankle function than to static lateral stress. The internally braced prosthesis of Regauer et al. established the technical feasibility of suture-tape reconstruction of the ATFL, the deep deltoid and the talocalcaneal ligaments [9]. The present work quantifies the comparative biomechanical effect of an analogous transosseous construct against both intact and unaugmented controls, across foot positions and under physiological load. In contrast to that previous four-ligament design, we reconstructed only the two restraints most relevant to coronal-plane mortise stability—the anterior talofibular and the deep deltoid ones. Adding further limbs would have lengthened the implantation procedure, and the instability localised to these two restraints supported the use of the simplified construct.

The measured pattern matched the study hypothesis. The unconstrained prosthesis failed selectively: it displaced most in the ATFL-challenging positions with regard to the lateral translational measure (talar shift) and in the deltoid-challenging positions regarding the peritalar calcaneofibular distance, while remaining close to the intact ankle in neutral stance, plantarflexion and dorsiflexion. The calcaneofibular distance did not considerably change in inversion, and the lateral talar shift changed far less in eversion than in inversion—a directional, partial double dissociation, being consistent with a ligament-specific rather than a generic stiffening effect. The suture construct, fixed at the lateral and medial malleoli, restrained the prosthesis in precisely those positions and left the non-provoked postures largely unchanged, so its benefit was position-specific—related to foot positions that challenged each reconstructed restraint—rather than distributed as a uniform tightening. The large off-target effect sizes at some combined positions and the broader, less position-specific hindfoot alignment response indicate that with five specimens the localisation is rather approximate than exclusive. Clinically, these findings give a biomechanical rationale for (1) reinforcing the talar attachments of the anterior talofibular and deep deltoid restraints when an unconstrained talar prosthesis is implanted, and (2) exploring such constructs across the range of foot positions rather than in neutral stance alone. Because the construct requires only two transosseous sutures at the malleoli without fixation to the calcaneus or the navicular, it adds little to the exposure already needed to implant the prosthesis. Whether the reconstruction changes symptoms, dislocation rate or implant survival is a question for a prospective clinical study.

Two features of the inference warrant emphasis. First, primary inference rests on the four pre-specified provoked contrasts corrected within the four-position family. Two effects were robust across the corrections—the talar shift reduction in inversion and the reopening of the calcaneofibular distance in eversion, and these are the firmest results. Second, with a sample size of five specimens, the study is powered only to detect large within-specimen effects. Consequently, non-significant and off-target contrasts should not be interpreted as evidence of no effect; rather, they indicate that any such effects were not estimated with sufficient precision to support firm conclusions.

Several strengths and limitations of the current study have to be mentioned. The within-specimen study design, in which each foot served as its own reference across all conditions, positions and loading scenarios, controlled for inter-specimen variability, and the tibia-referenced coordinate system maintained the comparability of the coronal-plane parameters across positions. The sample size of five specimens powered only large within-foot effects, and the results should be best interpreted through the pre-specified primary contrasts rather than within the full set of parameter comparisons. The donors were older than the usual candidates for total talar replacement, who are commonly present in middle age with post-traumatic or avascular collapse. Age-related cartilage thinning may influence prosthesis seating—a factor that cannot be accounted for when designing a geometry-matched prototype. Moreover, aged periarticular ligament and capsule may be more lax versus younger patients, which would tend to amplify rather than diminish the displacements seen here, so that a restraint stabilising lax tissue should remain useful in a stiffer joint. Conversely, greater joint laxity provides a larger displacement for the suture construct to correct, potentially reducing its absolute benefit in stiffer joints typically observed in younger candidates. Therefore, the current study design inherently limits the influence of donor age on the comparison between surgical conditions, foot positions and loading scenarios. The surgical conditions were tested in a fixed, irreversible sequence, so condition effects are collinear with test order and cannot be fully separated from any cumulative soft-tissue drift across each specimen’s sequential acquisitions. Two features bound this concern. First, the direction of the bias is known for the key contrast: the suture stabilisation was always tested last, after any accumulated laxity, yet it still has reduced displacements, so sequential drift can only underestimate its stabilising effect, not create it. Second, the tested intact ankle itself demonstrated no such drift, its load-induced displacements were at or below approximately 0.5 mm or 0.6° with no dependence on foot position or acquisition order. However, these observations directly bound drift only in the intact state; the more compliant implanted construct could, in principle, exhibit greater drift. Consequently, some of the displacements attributed to the unconstrained prosthesis—tested after the intact state—might partly reflect drift that could not be measured directly, since once the talus has been resected, the intact state could no longer be re-imaged for verification. Regardless, the transition from intact ankle to implanted construct represents a discrete structural change rather than a gradual temporal progression and can therefore not be corrected through acquisition-order adjustment. The provoked positions also load restraints that the construct does not reconstruct—the calcaneofibular ligament in inversion and the superficial deltoid in eversion—so the position-specific effects can be localised to the reconstructed arms only approximately and should not be interpreted as isolating the ATFL and deep deltoid alone. Cadaveric testing captures only the passive response and does not reproduce in vivo muscle action or gait. The provoked positions were held statically at fixed jig angles rather than produced by physiological loading, and utilising a polyamide prototype—rather than a medical-grade metallic clinical implant—is a recognised limitation of cadaveric TTR studies, although the preserved specimen-specific geometry maintains the translatability of the construct-stability findings. Beyond geometry, the prototype differs from a definitive metallic implant in surface finish, bearing friction and stiffness. A smoother and stiffer metallic bearing may seat and articulate differently, potentially altering the absolute displacements in either direction. Whether this position-dependent pattern is preserved in the presence of a metallic implant remains uncertain and could not be evaluated in the current study. In addition, despite preservation of the syndesmotic ligaments and interosseous membrane, the proximal embedding of the tibia and fibula and the mounting of the specimens in the loading frame during image acquisition may have affected the measured outcomes. Clinical translation will require validation using definitive metallic implants. Furthermore, anteroposterior translation of the talar centre was not included as an outcome measure. Although anterior drawer translation has been quantified radiographically in cadaveric TTR work [8], no validated weight-bearing CT protocol currently exists for its assessment in this setting. In addition, defining the talar centre using two points on the talar dome may not accurately represent the three-dimensional centroid of an asymmetric or degenerative talus.

## 5. Conclusions

In this human cadaveric weight-bearing CT imaging study, an unconstrained total talar replacement held its position in neutral stance but displaced under load in inversion and eversion—positions that challenge the native collateral ligaments. Supplementary suture stabilisation influenced the displacements selectively in the same positions, decreasing the talar shift in inversion and reopening the calcaneofibular distance in eversion toward the intact ankle state. These findings give a biomechanical rationale for reinforcing the talar attachments of the anterior talofibular and deep deltoid restraints when an unconstrained talar prosthesis is implanted, highlighting the value of evaluating such constructs under load across the range of foot positions rather than in neutral stance alone.

Two questions are particularly important for future prospective studies: (1) whether supplementary suture reinforcement reduces malalignment and dislocation rates of metallic implants in vivo; and (2) whether provoked-position weight-bearing CTs can serve as a postoperative assessment of construct stability. The present findings are derived from a cadaveric model and, on their own, do not establish a clinical indication, support routine clinical use or predict patient outcomes.

## Figures and Tables

**Table 1:** Reliability and measurement precision of load-induced change (value under 700 N load minus value under 10 N load), estimated over observers for the coronal-plane parameters talar shift (TAS), calcaneofibular distance (CFD), hindfoot alignment angle (HAA) and subtalar vertical angle (SVA), presented in terms of the Inter- and Intra-observer Intraclass Correlation Coefficient (ICC) with a 95% Confidence Interval (95% CI), standard deviation (SD) and standard error of measurement (SEM) related to a single observer’s (non-averaged) measurement, and minimal detectable change of one measurement in one specimen at 95% confidence (MDC_95_ = 1.96 × √2 × SEM).

Parameter	Inter-Observer ICC (95% CI)	Intra-Observer ICC (95% CI)	SD	SEM	MDC_95_
TAS (mm)	0.92 (0.89 to 0.94)	0.90 (0.88 to 0.92)	0.26	0.19	0.53
CFD (mm)	0.96 (0.94 to 0.97)	0.92 (0.91 to 0.94)	0.23	0.15	0.42
HAA (°)	0.92 (0.90 to 0.95)	0.88 (0.86 to 0.90)	0.36	0.25	0.69
SVA (°)	0.89 (0.84 to 0.92)	0.87 (0.85 to 0.90)	0.38	0.28	0.79

**Table 2:** Instability of unconstrained prosthesis: effect of surgical condition (TTR-Alone versus Intact-Native) on load-induced change (value under 700 N load minus value under 10 N load) of the coronal-plane parameters talar shift (TAS), hindfoot alignment angle (HAA), subtalar vertical angle (SVA) and calcaneofibular distance (CFD) in neutral and pre-specified provoked positions (inversion, PF-inversion, eversion and DF-eversion). Δ is the difference in load-induced change with its 95% Confidence Interval (95% CI, unadjusted for multiplicity). Cohen’s dz (dz) expresses effect size. Positive values denote varus situations with regard to TAS, HAA and SVA, and a wider subfibular space regarding CFD. *p*-values are Holm-corrected within the nine-position conservative sensitivity analysis over the full foot-position set (family B).

Parameter	Position	Δ (95% CI)	dz	*p*-Value
TAS (mm)	Neutral	+0.78 (−0.08 to +1.64)	+1.12	0.266
TAS (mm)	Inversion	+2.29 (+1.91 to +2.66)	+7.51	0.001
TAS (mm)	PF-inversion	+1.53 (+0.88 to +2.18)	+2.93	0.022
TAS (mm)	Eversion	+0.58 (−0.25 to +1.42)	+0.86	0.377
TAS (mm)	DF-eversion	+0.74 (+0.19 to +1.29)	+1.67	0.122
CFD (mm)	Neutral	+0.34 (+0.03 to +0.65)	+1.34	0.239
CFD (mm)	Inversion	+0.07 (−0.31 to +0.44)	+0.22	1.000
CFD (mm)	PF-inversion	−0.24 (−1.50 to +1.01)	−0.24	1.000
CFD (mm)	Eversion	−1.54 (−2.26 to −0.82)	−2.65	0.033
CFD (mm)	DF-eversion	−1.45 (−2.38 to −0.52)	−1.93	0.087
HAA (°)	Neutral	+0.32 (−0.44 to +1.09)	+0.52	0.682
HAA (°)	Inversion	+1.96 (+0.99 to +2.93)	+2.51	0.035
HAA (°)	PF-inversion	+0.50 (−0.33 to +1.33)	+0.74	0.682
HAA (°)	Eversion	+2.28 (+1.40 to +3.16)	+3.21	0.016
HAA (°)	DF-eversion	+1.91 (−0.05 to +3.87)	+1.21	0.325
SVA (°)	Neutral	+0.85 (+0.28 to +1.41)	+1.87	0.125
SVA (°)	Inversion	+0.83 (+0.08 to +1.57)	+1.38	0.183
SVA (°)	PF-inversion	+1.42 (+0.24 to +2.60)	+1.50	0.179
SVA (°)	Eversion	+1.23 (+0.25 to +2.21)	+1.55	0.179
SVA (°)	DF-eversion	+1.60 (−0.27 to +3.47)	+1.06	0.304

**Table 3:** Two-Way Repeated Measures ANOVA of the load-induced change, with surgical condition (three levels) and foot position (nine levels) as within-specimen factors. Degrees of freedom (df) and *p*-values are Greenhouse−Geisser-corrected with presented epsilon (ε_GG_); η^2^G is generalised eta-squared. With five specimens, the epsilon estimates for the interaction approach their lower bound, so the interaction is tested with little residual power.

Parameter	Effect	F	df	ε_GG_	*p*-Value	η^2^_G_
TAS (mm)	Condition	11.32	1.34, 5.36	0.670	0.015	0.56
TAS (mm)	Position	4.46	2.76, 11.04	0.345	0.030	0.32
TAS (mm)	Condition × position	6.88	2.80, 11.20	0.175	0.007	0.44
CFD (mm)	Condition	16.62	1.54, 6.14	0.768	0.004	0.33
CFD (mm)	Position	22.43	3.07, 12.28	0.384	<0.001	0.63
CFD (mm)	Condition × position	5.34	2.36, 9.44	0.147	0.025	0.40
HAA (°)	Condition	8.89	1.63, 6.52	0.815	0.016	0.38
HAA (°)	Position	6.02	2.51, 10.05	0.314	0.015	0.19
HAA (°)	Condition × position	3.82	2.40, 9.60	0.150	0.055	0.27
SVA (°)	Condition	4.46	1.97, 7.86	0.983	0.051	0.28
SVA (°)	Position	3.96	2.25, 8.99	0.281	0.055	0.12
SVA (°)	Condition × position	1.30	3.02, 12.09	0.189	0.319	0.18

## Data Availability

Data is available upon reasonable request.

## Supplementary Materials

The following supporting information can be downloaded at: https://www.mdpi.com/article/10.3390/bioengineering13090990/s1, Table S1: Demographic characteristics and mean individual parameter values across observers by surgical condition, foot position, and loading scenario; Table S2: Individual parameter values for each observer and measurement repeat (O1.1–O2.3) across surgical conditions, foot positions, and loading scenarios.

## References

[1] Pearce D.H., Mongiardi C.N., Fornasier V.L., Daniels T.R. (2005). Avascular necrosis of the talus: A pictorial essay. Radiographics.

[2] Kadakia R.J., Akoh C.C., Chen J., Sharma A., Parekh S.G. (2020). 3D Printed Total Talus Replacement for Avascular Necrosis of the Talus. Foot Ankle Int..

[3] Gross C.E., Sershon R.A., Frank J.M., Easley M.E., Holmes G.B. (2016). Treatment of Osteonecrosis of the Talus. JBJS Rev..

[4] Levinson J., Reissig J., Schaheen E., Lee W., Park J. (2021). Complications and Radiographic Outcomes After Tibiotalocalcaneal Fusion with a Retrograde Intramedullary Nail. Foot Ankle Spec..

[5] Taniguchi A., Tanaka Y. (2019). An Alumina Ceramic Total Talar Prosthesis for Avascular Necrosis of the Talus. Foot Ankle Clin..

[6] Morita S., Taniguchi A., Miyamoto T., Kurokawa H., Takakura Y., Takakura Y., Tanaka Y. (2022). The Long-Term Clinical Results of Total Talar Replacement at 10 Years or More After Surgery. J. Bone Jt. Surg. Am..

[7] West T.A., Rush S.M. (2021). Total Talus Replacement: Case Series and Literature Review. J. Foot Ankle Surg..

[8] Sato G., Saengsin J., Sornsakrin P., Bhimani R., Lubberts B., Taniguchi A., DiGiovanni C., Tanaka Y. (2022). The stability of total talar prosthesis-How stable to dislocation? Cadaveric study. J. Orthop. Res..

[9] Regauer M., Lange M., Soldan K., Peyerl S., Baumbach S., Bocker W., Polzer H. (2017). Development of an internally braced prosthesis for total talus replacement. World J. Orthop..

[10] Anastasio A.T., Tabarestani T.Q., Schweitzer K.M. (2023). Exploring the Possibilities of the Custom Total Ankle Total Talus Replacement (TATTR): Short-Form Technique for Adjunctive Lateral Ligamentous Reconstruction with TATTR. Foot Ankle Orthop..

[11] Angthong C., Rajbhandari P. (2020). Total talar prosthesis with and without ankle ligament reconstruction using the three-dimensional computer-aided design and computer numerical control manufacturing techniques. Orthop. Rev..

[12] Pellegrini M.J., Glisson R.R., Wurm M., Ousema P.H., Romash M.M., Nunley J.A., Easley M.E. (2016). Systematic Quantification of Stabilizing Effects of Subtalar Joint Soft-Tissue Constraints in a Novel Cadaveric Model. J. Bone Jt. Surg. Am..

[13] Krahenbuhl N., Horn-Lang T., Hintermann B., Knupp M. (2017). The subtalar joint: A complex mechanism. EFORT Open Rev..

[14] Pastor T., Zderic I., van Knegsel K.P., Berk T., Mechkarska R., Beeres F.J.P., Gueorguiev B., Pastor T. (2025). How many knots are necessary to achieve knot security of two high strength suture tapes? A biomechanical comparative analysis. Arch. Orthop. Trauma Surg..

[15] Souleiman F., Heilemann M., Hennings R., Hepp P., Gueorguiev B., Richards G., Osterhoff G., Gehweiler D. (2022). Effect of weightbearing and foot positioning on 3D distal tibiofibular joint parameters. Sci. Rep..

[16] Dullaert K., Hagen J., Klos K., Gueorguiev B., Lenz M., Richards R.G., Simons P. (2016). The influence of the Peroneus Longus muscle on the foot under axial loading: A CT evaluated dynamic cadaveric model study. Clin. Biomech..

[17] Burssens A., Peeters J., Buedts K., Victor J., Vandeputte G. (2016). Measuring hindfoot alignment in weight bearing CT: A novel clinical relevant measurement method. Foot Ankle Surg..

[18] Jeng C.L., Rutherford T., Hull M.G., Cerrato R.A., Campbell J.T. (2019). Assessment of Bony Subfibular Impingement in Flatfoot Patients Using Weight-Bearing CT Scans. Foot Ankle Int..

[19] de Cesar Netto C., Shakoor D., Dein E.J., Zhang H., Thawait G.K., Richter M., Ficke J.R., Schon L.C., Demehri S., Weightbearing CT International Study Group (2019). Influence of investigator experience on reliability of adult acquired flatfoot deformity measurements using weightbearing computed tomography. Foot Ankle Surg..

[20] Nomkhondorj O., Chun D.I., Park K.R., Cho J. (2025). Weightbearing Computed Tomography (WBCT) Analysis of Subtalar Joint Dynamics in Hindfoot Valgus Malalignment. J. Clin. Med..

[21] Krahenbuhl N., Tschuck M., Bolliger L., Hintermann B., Knupp M. (2016). Orientation of the Subtalar Joint: Measurement and Reliability Using Weightbearing CT Scans. Foot Ankle Int..

[22] Van Bergeyk A.B., Younger A., Carson B. (2002). CT analysis of hindfoot alignment in chronic lateral ankle instability. Foot Ankle Int..

[23] Lintz F., Welck M., Bernasconi A., Thornton J., Cullen N.P., Singh D., Goldberg A. (2017). 3D Biometrics for Hindfoot Alignment Using Weightbearing CT. Foot Ankle Int..

[24] Rojas E.O., Barbachan Mansur N.S., Dibbern K., Lalevee M., Auch E., Schmidt E., Vivtcharenko V., Li S., Phisitkul P., Femino J. (2021). Weightbearing Computed Tomography for Assessment of Foot and Ankle Deformities: The Iowa Experience. Iowa Orthop. J..

[25] Fedorov A., Beichel R., Kalpathy-Cramer J., Finet J., Fillion-Robin J.C., Pujol S., Bauer C., Jennings D., Fennessy F., Sonka M. (2012). 3D Slicer as an image computing platform for the Quantitative Imaging Network. Magn. Reson. Imaging.

[26] Koo T.K., Li M.Y. (2016). A Guideline of Selecting and Reporting Intraclass Correlation Coefficients for Reliability Research. J. Chiropr. Med..

