# Supplementary material for: Position-Dependent Stability of an Unconstrained Total Talar Replacement and Its Suture Reinforcement: A Human Cadaveric Weight-Bearing Computed Tomography Study
Source: Bioengineering (Basel). 2026 Aug 27;13(9):990. doi: 10.3390/bioengineering13090990 (PMC13603971; doi:10.3390/bioengineering13090990)
Supplement: Supplementary file 1 [file bioengineering-13-00990-s001.zip › bioengineering-4506078-supplementary.pdf]

Table S1. Demographic characteristics and mean individual parameter values across observers by surgical condition, foot position, and loading scenario

						TAS (mm)		CFD (mm)		HAA (°)		SVA (°)		FAO (%)	
Specimen	Sex	Age	Side	Condition	Position	10 N	700 N	10 N	700 N	10 N	700 N	10 N	700 N	10 N	700 N
1	F	65	R	Intact-Native	N	-3.100	-2.478	6.526	6.241	-5.351	-4.541	10.310	10.490	8.739	8.961
1	F	65	R	Intact-Native	PF	-3.350	-2.417	6.652	6.855	-5.606	-4.899	10.129	11.515		
1	F	65	R	Intact-Native	DF	-2.945	-2.233	6.105	6.212	-5.078	-4.794	10.647	11.456		
1	F	65	R	Intact-Native	EV	-4.273	-4.241	4.442	4.340	-8.681	-8.860	7.820	7.095		
1	F	65	R	Intact-Native	IN	-1.839	-1.724	8.137	8.208	-1.869	-2.006	13.383	13.560		
1	F	65	R	Intact-Native	PFE	-4.418	-3.817	4.724	4.777	-8.693	-8.224	7.733	7.897		
1	F	65	R	Intact-Native	DFE	-4.294	-3.593	4.383	4.418	-8.124	-7.766	7.958	8.575		
1	F	65	R	Intact-Native	PFI	-2.020	-1.821	7.769	8.190	-2.043	-2.401	12.838	11.974		
1	F	65	R	Intact-Native	DFI	-1.579	-0.860	8.107	8.957	-1.616	-1.262	13.248	13.838		
1	F	65	R	TTR-Alone	N	-2.874	-1.607	6.107	6.419	-4.822	-3.486	10.849	11.471	7.373	7.593
1	F	65	R	TTR-Alone	PF	-2.860	-1.877	6.280	5.712	-5.218	-4.086	10.478	11.061		
1	F	65	R	TTR-Alone	DF	-2.589	-1.616	5.816	6.096	-4.549	-3.195	11.255	11.255		
1	F	65	R	TTR-Alone	EV	-3.980	-3.630	4.303	2.897	-8.218	-6.052	8.119	9.636		
1	F	65	R	TTR-Alone	IN	-1.339	0.851	7.765	8.150	-1.280	0.587	13.661	14.250		
1	F	65	R	TTR-Alone	PFE	-3.989	-3.021	4.714	3.159	-8.157	-7.311	8.095	8.088		
1	F	65	R	TTR-Alone	DFE	-3.726	-3.043	4.167	3.430	-7.898	-6.197	8.575	8.409		
1	F	65	R	TTR-Alone	PFI	-1.640	-0.228	7.575	8.401	-1.787	-1.833	13.257	14.338		
1	F	65	R	TTR-Alone	DFI	-1.367	-0.453	7.937	8.437	-1.058	0.308	13.875	14.097		
1	F	65	R	TTR-Suture	N	-2.942	-2.308	6.330	6.280	-5.063	-5.069	10.412	10.556	4.557	4.777
1	F	65	R	TTR-Suture	PF	-3.056	-2.449	6.455	6.571	-5.389	-5.196	10.209	9.634		
1	F	65	R	TTR-Suture	DF	-2.852	-2.441	5.975	6.186	-4.847	-4.092	10.927	11.024		
1	F	65	R	TTR-Suture	EV	-4.239	-4.037	4.344	4.426	-8.248	-7.244	8.016	8.818		
1	F	65	R	TTR-Suture	IN	-1.432	-0.542	8.159	8.185	-1.459	-0.310	13.625	14.453		
1	F	65	R	TTR-Suture	PFE	-4.350	-3.704	4.371	3.409	-8.342	-8.100	7.893	8.682		
1	F	65	R	TTR-Suture	DFE	-3.967	-2.708	4.232	4.219	-8.069	-7.146	8.300	8.005		
1	F	65	R	TTR-Suture	PFI	-1.735	-0.433	7.734	8.731	-1.985	-1.695	13.103	13.522		
1	F	65	R	TTR-Suture	DFI	-1.519	-1.243	8.167	8.991	-1.341	-0.807	13.556	13.831		
2	M	71	L	Intact-Native	N	-1.428	-1.061	7.845	7.332	-3.448	-3.673	13.831	12.795	8.859	9.079
2	M	71	L	Intact-Native	PF	-1.650	-1.509	7.975	7.628	-3.717	-3.897	13.472	13.376		
2	M	71	L	Intact-Native	DF	-1.289	-1.372	7.715	6.063	-3.134	-3.891	14.506	14.422		
2	M	71	L	Intact-Native	EV	-2.481	-3.476	6.426	5.673	-5.802	-6.472	11.849	11.051		
2	M	71	L	Intact-Native	IN	-0.424	-0.620	8.989	8.318	-0.531	-1.087	16.031	15.634		
2	M	71	L	Intact-Native	PFE	-2.897	-2.473	6.446	6.383	-6.377	-6.127	11.423	11.353		
2	M	71	L	Intact-Native	DFE	-2.376	-2.613	6.001	5.358	-5.772	-5.821	11.941	10.884		
2	M	71	L	Intact-Native	PFI	-0.393	-0.417	9.199	9.702	-0.446	-1.013	16.075	15.308		
2	M	71	L	Intact-Native	DFI	-0.166	0.116	9.524	9.331	-0.094	-0.924	16.419	15.765		
2	M	71	L	TTR-Alone	N	-1.047	0.190	7.533	6.993	-2.948	-2.399	14.405	14.894	8.102	8.322
2	M	71	L	TTR-Alone	PF	-1.418	-0.145	7.842	7.223	-3.180	-2.779	13.963	13.986		
2	M	71	L	TTR-Alone	DF	-0.984	-0.289	7.409	6.747	-2.524	-2.287	14.663	14.358		
2	M	71	L	TTR-Alone	EV	-2.057	-1.617	6.256	4.191	-5.300	-3.467	12.364	13.334		
2	M	71	L	TTR-Alone	IN	0.027	2.555	8.927	8.082	-0.040	2.231	16.388	17.678		
2	M	71	L	TTR-Alone	PFE	-2.502	-0.856	6.008	4.096	-5.821	-6.134	11.811	10.897		
2	M	71	L	TTR-Alone	DFE	-2.021	-1.142	5.988	4.339	-5.304	-4.409	12.402	13.270		
2	M	71	L	TTR-Alone	PFI	-0.083	1.996	8.844	7.553	-0.034	0.668	16.473	17.044		
2	M	71	L	TTR-Alone	DFI	0.166	1.124	9.003	8.810	0.304	0.963	16.738	17.587		
2	M	71	L	TTR-Suture	N	-1.241	0.206	7.657	7.139	-2.962	-3.707	14.159	13.757	10.821	11.041
2	M	71	L	TTR-Suture	PF	-1.606	-0.952	7.834	7.444	-3.665	-3.940	13.512	14.740		
2	M	71	L	TTR-Suture	DF	-1.234	-0.289	7.564	8.740	-2.937	-3.205	14.441	15.078		
2	M	71	L	TTR-Suture	EV	-2.173	-1.032	6.408	5.698	-5.530	-5.566	12.219	12.576		
2	M	71	L	TTR-Suture	IN	-0.190	0.412	9.001	7.931	-0.546	-0.845	16.400	15.259		
2	M	71	L	TTR-Suture	PFE	-2.442	-2.264	6.234	4.887	-6.035	-6.415	11.621	13.366		
2	M	71	L	TTR-Suture	DFE	-2.228	-1.394	6.178	4.708	-5.613	-5.823	12.191	12.830		
2	M	71	L	TTR-Suture	PFI	-0.458	0.632	9.116	7.993	-0.227	-1.127	16.341	16.742		
2	M	71	L	TTR-Suture	DFI	0.069	1.320	9.193	8.895	0.106	0.560	16.458	16.942		
3	F	72	R	Intact-Native	N	-2.640	-1.930	5.880	5.674	-4.823	-3.653	12.359	12.960	9.670	9.890
3	F	72	R	Intact-Native	PF	-2.655	-2.037	6.020	6.005	-5.464	-5.111	12.066	12.862		
3	F	72	R	Intact-Native	DF	-2.505	-1.943	5.692	5.458	-4.724	-4.499	12.605	13.361		
3	F	72	R	Intact-Native	EV	-3.787	-3.363	4.113	4.237	-7.854	-7.841	9.909	9.814		
3	F	72	R	Intact-Native	IN	-1.438	-1.671	7.405	6.647	-1.539	-2.146	14.809	14.346		
3	F	72	R	Intact-Native	PFE	-3.739	-2.923	4.753	4.948	-7.722	-6.987	10.034	10.484		
3	F	72	R	Intact-Native	DFE	-3.601	-3.036	4.301	4.238	-7.443	-8.444	10.429	9.762		
3	F	72	R	Intact-Native	PFI	-1.571	-0.930	7.012	7.656	-2.378	-2.157	14.443	14.283		
3	F	72	R	Intact-Native	DFI	-1.466	-0.765	7.387	7.743	-1.815	-1.504	14.764	15.311		
3	F	72	R	TTR-Alone	N	-2.271	0.349	5.667	5.743	-4.447	-4.042	12.757	13.787	9.668	9.888
3	F	72	R	TTR-Alone	PF	-2.423	-1.728	5.803	6.129	-4.590	-4.513	12.334	13.375		
3	F	72	R	TTR-Alone	DF	-2.118	-0.888	5.506	5.250	-4.265	-2.371	12.928	13.393		
3	F	72	R	TTR-Alone	EV	-3.391	-2.240	3.974	1.636	-7.579	-5.090	10.335	11.390		
3	F	72	R	TTR-Alone	IN	-0.863	1.393	7.252	6.897	-1.201	0.766	15.427	16.183		
3	F	72	R	TTR-Alone	PFE	-3.391	-2.658	4.145	2.373	-6.968	-5.368	10.387	12.667		
3	F	72	R	TTR-Alone	DFE	-3.198	-1.674	3.777	1.200	-6.766	-3.593	10.815	13.241		
3	F	72	R	TTR-Alone	PFI	-1.288	0.529	6.794	6.900	-1.834	-1.531	14.704	17.282		
3	F	72	R	TTR-Alone	DFI	-1.044	0.143	6.989	7.207	-1.258	0.354	15.102	16.415		
3	F	72	R	TTR-Suture	N	-2.485	0.080	5.731	5.111	-4.656	-2.100	12.441	12.315	8.090	8.310
3	F	72	R	TTR-Suture	PF	-2.580	-1.239	5.935	5.014	-5.111	-3.204	12.088	14.246		
3	F	72	R	TTR-Suture	DF	-2.340	-0.843	5.577	5.146	-4.483	-3.310	12.681	14.351		
3	F	72	R	TTR-Suture	EV	-3.536	-2.132	4.039	2.596	-7.824	-5.412	10.059	11.073		
3	F	72	R	TTR-Suture	IN	-1.255	-0.031	7.150	6.417	-1.372	-0.032	15.117	17.034		
3	F	72	R	TTR-Suture	PFE	-3.613	-2.092	4.626	4.078	-7.495	-6.410	10.336	10.124		
3	F	72	R	TTR-Suture	DFE	-3.352	-1.409	4.122	2.781	-7.321	-4.831	10.540	11.633		
3	F	72	R	TTR-Suture	PFI	-1.415	0.543	7.009	7.474	-2.028	-0.774	14.597	14.899		
3	F	72	R	TTR-Suture	DFI	-1.164	0.090	7.434	7.737	-1.563	-0.388	14.943	16.076		
4	F	72	L	Intact-Native	N	-0.946	-0.625	7.088	6.533	-0.992	-1.407	14.634	13.378	13.172	13.392
4	F	72	L	Intact-Native	PF	-1.118	-0.635	7.196	7.086	-1.460	-1.257	14.109	14.653		

4	F	72	L	Intact-Native	DF	-0.831	-0.018	6.948	7.276	-0.818	-0.419	15.114	16.012		
4	F	72	L	Intact-Native	EV	-1.891	-1.168	5.821	6.173	-3.404	-3.230	12.956	12.489		
4	F	72	L	Intact-Native	IN	0.110	0.236	8.430	8.525	1.438	1.315	16.479	16.673		
4	F	72	L	Intact-Native	PFE	-1.924	-1.366	5.678	5.703	-3.806	-3.390	12.438	12.545		
4	F	72	L	Intact-Native	DFE	-1.745	-1.753	5.610	5.132	-3.369	-4.023	12.701	11.312		
4	F	72	L	Intact-Native	PFI	-0.010	0.405	8.229	8.523	1.371	1.221	16.551	16.430		
4	F	72	L	Intact-Native	DFI	0.267	0.409	8.499	9.311	1.698	0.487	16.731	15.677		
4	F	72	L	TTR-Alone	N	-0.587	-0.100	6.798	6.791	-0.643	-0.549	14.980	14.570	12.925	13.145
4	F	72	L	TTR-Alone	PF	-0.687	0.084	7.016	6.563	-0.988	-0.231	14.599	14.525		
4	F	72	L	TTR-Alone	DF	-0.292	0.716	6.575	7.010	-0.245	-0.217	15.437	15.136		
4	F	72	L	TTR-Alone	EV	-1.483	-1.148	5.497	4.178	-2.801	-1.545	13.214	13.043		
4	F	72	L	TTR-Alone	IN	0.295	2.464	8.074	8.254	1.974	2.798	16.914	17.457		
4	F	72	L	TTR-Alone	PFE	-1.589	-0.861	5.640	4.622	-3.285	-4.785	13.128	14.624		
4	F	72	L	TTR-Alone	DFE	-1.348	-0.597	5.116	3.646	-2.692	-3.097	13.323	14.479		
4	F	72	L	TTR-Alone	PFI	0.380	1.866	7.872	9.015	1.906	1.469	16.923	17.154		
4	F	72	L	TTR-Alone	DFI	0.721	1.562	8.241	9.070	2.264	2.565	17.233	17.770		
4	F	72	L	TTR-Suture	N	-0.642	-0.302	6.906	7.165	-0.846	-1.048	14.897	15.002	9.221	9.441
4	F	72	L	TTR-Suture	PF	-1.061	-0.594	7.173	6.672	-1.158	-1.509	14.481	13.845		
4	F	72	L	TTR-Suture	DF	-0.405	-0.271	6.746	6.681	-0.610	-0.612	15.169	14.073		
4	F	72	L	TTR-Suture	EV	-1.566	-1.539	5.708	6.149	-3.213	-3.345	13.076	13.892		
4	F	72	L	TTR-Suture	IN	0.236	0.756	8.082	7.986	1.487	1.326	16.673	16.094		
4	F	72	L	TTR-Suture	PFE	-1.655	-1.022	5.718	5.446	-3.479	-4.384	12.580	10.751		
4	F	72	L	TTR-Suture	DFE	-1.707	-1.127	5.497	5.297	-3.208	-3.221	13.003	12.583		
4	F	72	L	TTR-Suture	PFI	0.026	0.302	8.147	8.815	1.472	1.174	16.826	16.097		
4	F	72	L	TTR-Suture	DFI	0.259	-0.348	8.216	8.649	2.079	2.014	16.999	16.889		
5	M	81	R	Intact-Native	N	-2.084	-1.735	6.578	6.048	-2.884	-3.183	11.710	10.589	4.959	5.179
5	M	81	R	Intact-Native	PF	-2.292	-1.879	6.754	6.596	-3.223	-3.098	11.384	11.796		
5	M	81	R	Intact-Native	DF	-1.928	-1.405	6.424	6.116	-2.461	-2.313	12.168	12.853		
5	M	81	R	Intact-Native	EV	-3.244	-3.698	4.962	4.571	-5.794	-6.175	9.487	8.900		
5	M	81	R	Intact-Native	IN	-0.790	-0.835	8.094	7.785	0.377	0.025	14.294	14.176		
5	M	81	R	Intact-Native	PFE	-3.301	-3.100	5.087	4.878	-6.108	-6.133	9.152	8.786		
5	M	81	R	Intact-Native	DFE	-2.910	-2.424	4.814	4.693	-5.654	-6.458	9.691	9.087		
5	M	81	R	Intact-Native	PFI	-0.928	-0.629	7.922	8.289	0.081	-0.154	14.450	13.584		
5	M	81	R	Intact-Native	DFI	-0.497	0.235	8.342	8.919	0.624	1.014	14.650	15.282		
5	M	81	R	TTR-Alone	N	-1.562	-0.923	6.359	6.110	-2.265	-2.004	12.302	12.165	10.333	10.553
5	M	81	R	TTR-Alone	PF	-1.915	-1.175	6.479	5.875	-2.749	-2.471	11.722	12.189		
5	M	81	R	TTR-Alone	DF	-1.643	-0.743	6.188	6.195	-1.872	-1.660	12.775	12.712		
5	M	81	R	TTR-Alone	EV	-2.836	-2.476	4.862	3.540	-5.356	-2.761	10.102	10.193		
5	M	81	R	TTR-Alone	IN	-0.543	1.513	8.062	7.458	0.980	2.060	14.841	15.182		
5	M	81	R	TTR-Alone	PFE	-2.839	-2.645	4.721	3.128	-5.656	-5.191	9.687	8.744		
5	M	81	R	TTR-Alone	DFE	-2.523	-1.169	4.553	2.486	-5.165	-3.146	10.346	10.966		
5	M	81	R	TTR-Alone	PFI	-0.597	1.809	7.599	7.821	0.742	1.615	14.830	14.709		
5	M	81	R	TTR-Alone	DFI	-0.230	0.446	8.059	8.105	1.158	2.313	15.194	14.484		
5	M	81	R	TTR-Suture	N	-1.890	-0.973	6.465	6.677	-2.724	-2.521	11.990	12.801	7.211	7.431
5	M	81	R	TTR-Suture	PF	-2.253	-1.842	6.861	6.447	-2.907	-2.465	11.699	12.554		
5	M	81	R	TTR-Suture	DF	-1.836	-1.221	6.181	6.580	-2.257	-2.057	12.439	12.777		
5	M	81	R	TTR-Suture	EV	-2.981	-2.658	4.850	4.884	-5.512	-5.063	9.822	9.640		
5	M	81	R	TTR-Suture	IN	-0.642	0.074	7.848	7.614	0.670	1.155	14.552	14.971		
5	M	81	R	TTR-Suture	PFE	-3.132	-2.719	4.865	3.992	-5.878	-6.060	9.545	10.508		
5	M	81	R	TTR-Suture	DFE	-2.975	-1.880	4.681	3.309	-5.257	-4.212	9.813	9.835		
5	M	81	R	TTR-Suture	PFI	-0.675	0.602	7.809	8.002	0.366	-0.234	14.364	15.449		
5	M	81	R	TTR-Suture	DFI	-0.348	0.964	8.211	9.067	0.919	1.104	14.592	14.411		

Table S2. Individual parameter values for each observer and measurement repeat (O1.1–O2.3) across surgical conditions, foot positions, and loading scenarios

						Observer 1			Observer 2		
Specimen	Condition	Position	Load (N)	Parameter	Unit	O1.1	O1.2	O1.3	O2.1	O2.2	O2.3
1	Intact-Native	N	10	TAS	mm	-3.183	-3.222	-2.568	-3.403	-3.370	-2.851
1	Intact-Native	N	10	CFD	mm	6.536	6.477	6.320	6.380	6.695	6.745
1	Intact-Native	N	10	HAA	deg	-5.158	-5.202	-5.156	-5.609	-5.716	-5.263
1	Intact-Native	N	10	SVA	deg	10.513	10.029	10.743	9.779	10.584	10.211
1	Intact-Native	N	10	FAO	percent	8.729	8.808	8.800	8.697	8.708	8.695
1	Intact-Native	N	700	TAS	mm	-2.252	-2.723	-2.449	-2.564	-2.404	-2.477
1	Intact-Native	N	700	CFD	mm	6.439	6.165	6.297	6.129	6.320	6.095
1	Intact-Native	N	700	HAA	deg	-4.352	-4.396	-4.673	-4.746	-4.533	-4.546
1	Intact-Native	N	700	SVA	deg	11.135	10.174	10.514	10.158	10.442	10.516
1	Intact-Native	N	700	FAO	percent	8.965	8.967	9.019	8.925	8.887	9.000
1	Intact-Native	PF	10	TAS	mm	-3.474	-3.280	-3.342	-3.590	-3.096	-3.321
1	Intact-Native	PF	10	CFD	mm	6.768	6.477	6.989	6.517	6.391	6.769
1	Intact-Native	PF	10	HAA	deg	-5.604	-5.885	-5.706	-5.593	-5.357	-5.493
1	Intact-Native	PF	10	SVA	deg	9.772	9.926	10.449	9.840	9.924	10.862
1	Intact-Native	PF	700	TAS	mm	-2.720	-2.327	-2.066	-2.802	-2.138	-2.447
1	Intact-Native	PF	700	CFD	mm	6.782	6.826	7.201	6.714	6.504	7.103
1	Intact-Native	PF	700	HAA	deg	-4.988	-4.944	-4.742	-5.406	-4.769	-4.544
1	Intact-Native	PF	700	SVA	deg	10.947	11.396	11.868	11.782	11.077	12.021
1	Intact-Native	DF	10	TAS	mm	-2.392	-3.183	-2.794	-3.320	-2.806	-3.175
1	Intact-Native	DF	10	CFD	mm	6.259	6.112	6.228	6.146	6.054	5.830
1	Intact-Native	DF	10	HAA	deg	-4.796	-5.377	-5.311	-5.007	-5.195	-4.779
1	Intact-Native	DF	10	SVA	deg	11.301	10.755	10.556	9.926	10.742	10.602
1	Intact-Native	DF	700	TAS	mm	-2.239	-2.286	-2.074	-2.200	-2.230	-2.370
1	Intact-Native	DF	700	CFD	mm	6.015	6.171	6.062	6.648	6.166	6.213
1	Intact-Native	DF	700	HAA	deg	-5.026	-4.796	-4.906	-4.447	-4.722	-4.864
1	Intact-Native	DF	700	SVA	deg	11.400	11.303	11.374	11.331	11.678	11.650
1	Intact-Native	EV	10	TAS	mm	-4.049	-4.516	-4.240	-4.010	-4.529	-4.293
1	Intact-Native	EV	10	CFD	mm	4.456	4.704	4.155	4.306	4.443	4.587
1	Intact-Native	EV	10	HAA	deg	-8.517	-8.635	-8.647	-8.624	-8.717	-8.945
1	Intact-Native	EV	10	SVA	deg	7.782	7.966	8.013	7.856	7.588	7.714
1	Intact-Native	EV	700	TAS	mm	-4.143	-4.464	-4.143	-3.960	-4.659	-4.076
1	Intact-Native	EV	700	CFD	mm	4.182	4.359	4.459	4.297	4.288	4.452
1	Intact-Native	EV	700	HAA	deg	-8.947	-8.973	-8.914	-8.316	-8.975	-9.032
1	Intact-Native	EV	700	SVA	deg	7.122	7.137	7.197	7.320	6.734	7.059
1	Intact-Native	IN	10	TAS	mm	-1.562	-1.831	-1.668	-1.861	-2.096	-2.017
1	Intact-Native	IN	10	CFD	mm	8.152	8.262	8.065	8.007	7.959	8.380
1	Intact-Native	IN	10	HAA	deg	-1.711	-1.426	-1.593	-2.406	-2.407	-1.673
1	Intact-Native	IN	10	SVA	deg	13.845	13.100	13.677	13.405	13.256	13.015
1	Intact-Native	IN	700	TAS	mm	-1.848	-1.865	-1.833	-1.524	-1.448	-1.826
1	Intact-Native	IN	700	CFD	mm	8.150	8.266	8.319	8.163	8.119	8.232
1	Intact-Native	IN	700	HAA	deg	-1.816	-1.950	-1.754	-2.290	-2.174	-2.050
1	Intact-Native	IN	700	SVA	deg	14.034	13.395	13.790	13.557	13.661	12.926
1	Intact-Native	PFE	10	TAS	mm	-4.676	-4.534	-4.558	-4.484	-4.121	-4.138
1	Intact-Native	PFE	10	CFD	mm	4.550	4.616	4.737	4.861	4.700	4.880
1	Intact-Native	PFE	10	HAA	deg	-8.627	-9.070	-8.966	-8.679	-8.309	-8.507
1	Intact-Native	PFE	10	SVA	deg	7.651	7.330	8.048	7.779	7.828	7.763
1	Intact-Native	PFE	700	TAS	mm	-3.730	-3.754	-3.909	-3.718	-4.190	-3.602
1	Intact-Native	PFE	700	CFD	mm	4.894	4.773	4.662	5.001	4.449	4.885
1	Intact-Native	PFE	700	HAA	deg	-8.308	-8.072	-8.190	-8.193	-8.712	-7.871
1	Intact-Native	PFE	700	SVA	deg	7.970	7.567	8.159	8.067	7.917	7.701
1	Intact-Native	DFE	10	TAS	mm	-4.532	-4.170	-3.933	-4.383	-4.428	-4.317
1	Intact-Native	DFE	10	CFD	mm	4.312	4.221	4.370	4.410	4.545	4.441
1	Intact-Native	DFE	10	HAA	deg	-8.197	-7.999	-8.204	-8.067	-8.127	-8.152
1	Intact-Native	DFE	10	SVA	deg	7.841	8.016	7.561	8.241	7.998	8.088
1	Intact-Native	DFE	700	TAS	mm	-3.896	-3.456	-3.527	-3.512	-3.778	-3.391
1	Intact-Native	DFE	700	CFD	mm	4.354	4.434	4.592	4.197	4.583	4.349
1	Intact-Native	DFE	700	HAA	deg	-7.708	-7.844	-7.901	-7.638	-8.216	-7.286
1	Intact-Native	DFE	700	SVA	deg	8.514	8.677	8.026	8.823	8.452	8.959
1	Intact-Native	PFI	10	TAS	mm	-1.988	-1.746	-1.978	-2.148	-2.273	-1.986
1	Intact-Native	PFI	10	CFD	mm	7.559	7.621	8.015	7.720	7.694	8.004
1	Intact-Native	PFI	10	HAA	deg	-1.595	-1.869	-2.213	-2.186	-2.154	-2.240
1	Intact-Native	PFI	10	SVA	deg	13.015	12.440	12.426	12.964	12.895	13.289
1	Intact-Native	PFI	700	TAS	mm	-1.980	-1.611	-2.255	-1.656	-2.026	-1.396
1	Intact-Native	PFI	700	CFD	mm	7.844	7.984	8.479	7.998	8.206	8.626
1	Intact-Native	PFI	700	HAA	deg	-2.459	-2.365	-2.751	-2.232	-2.534	-2.067
1	Intact-Native	PFI	700	SVA	deg	11.834	11.600	12.139	12.253	11.490	12.528

1	Intact-Native	DFI	10	TAS	mm	-1.455	-1.784	-1.771	-1.494	-1.524	-1.444
1	Intact-Native	DFI	10	CFD	mm	8.246	7.970	7.941	8.360	7.988	8.137
1	Intact-Native	DFI	10	HAA	deg	-1.568	-1.543	-1.842	-1.575	-1.482	-1.686
1	Intact-Native	DFI	10	SVA	deg	13.365	13.413	13.016	13.540	13.456	12.696
1	Intact-Native	DFI	700	TAS	mm	-0.486	-1.042	-0.993	-0.707	-1.123	-0.812
1	Intact-Native	DFI	700	CFD	mm	9.179	9.133	8.813	8.749	8.881	8.988
1	Intact-Native	DFI	700	HAA	deg	-0.310	-1.376	-1.344	-1.331	-1.935	-1.273
1	Intact-Native	DFI	700	SVA	deg	13.977	13.604	13.423	13.939	14.157	13.930
1	TTR-Alone	N	10	TAS	mm	-2.784	-3.165	-3.030	-2.617	-3.017	-2.629
1	TTR-Alone	N	10	CFD	mm	5.820	5.746	6.307	6.236	6.211	6.322
1	TTR-Alone	N	10	HAA	deg	-4.927	-5.263	-5.242	-4.325	-4.554	-4.620
1	TTR-Alone	N	10	SVA	deg	10.907	10.552	10.942	11.210	10.948	10.533
1	TTR-Alone	N	10	FAO	percent	7.364	7.382	7.434	7.403	7.329	7.324
1	TTR-Alone	N	700	TAS	mm	-1.599	-1.736	-1.564	-1.488	-1.708	-1.545
1	TTR-Alone	N	700	CFD	mm	6.419	6.046	6.580	6.396	6.585	6.487
1	TTR-Alone	N	700	HAA	deg	-3.387	-3.494	-3.331	-3.478	-3.559	-3.667
1	TTR-Alone	N	700	SVA	deg	11.652	11.319	11.519	11.614	11.380	11.344
1	TTR-Alone	N	700	FAO	percent	7.624	7.622	7.522	7.602	7.567	7.619
1	TTR-Alone	PF	10	TAS	mm	-2.794	-2.909	-3.062	-2.915	-2.695	-2.787
1	TTR-Alone	PF	10	CFD	mm	6.287	6.098	6.351	6.219	6.654	6.069
1	TTR-Alone	PF	10	HAA	deg	-5.158	-5.022	-5.421	-5.049	-5.219	-5.440
1	TTR-Alone	PF	10	SVA	deg	10.552	10.859	10.357	11.078	10.028	9.997
1	TTR-Alone	PF	700	TAS	mm	-1.740	-1.876	-1.969	-1.880	-1.943	-1.856
1	TTR-Alone	PF	700	CFD	mm	5.754	5.543	5.771	5.716	5.813	5.677
1	TTR-Alone	PF	700	HAA	deg	-3.835	-4.369	-4.125	-4.065	-4.067	-4.054
1	TTR-Alone	PF	700	SVA	deg	11.220	11.025	11.365	11.307	11.041	10.407
1	TTR-Alone	DF	10	TAS	mm	-2.588	-2.712	-2.863	-2.286	-2.547	-2.539
1	TTR-Alone	DF	10	CFD	mm	5.717	6.060	5.662	5.775	5.939	5.744
1	TTR-Alone	DF	10	HAA	deg	-4.513	-4.424	-4.648	-4.496	-4.349	-4.864
1	TTR-Alone	DF	10	SVA	deg	11.578	11.602	11.082	10.924	11.362	10.980
1	TTR-Alone	DF	700	TAS	mm	-1.635	-1.557	-1.550	-1.761	-1.692	-1.504
1	TTR-Alone	DF	700	CFD	mm	5.947	6.342	6.207	5.831	5.956	6.294
1	TTR-Alone	DF	700	HAA	deg	-3.086	-3.205	-3.112	-3.397	-2.996	-3.372
1	TTR-Alone	DF	700	SVA	deg	11.065	11.411	11.283	11.341	11.567	10.862
1	TTR-Alone	EV	10	TAS	mm	-3.909	-4.055	-3.753	-4.206	-3.901	-4.057
1	TTR-Alone	EV	10	CFD	mm	4.269	4.302	3.939	4.279	4.350	4.677
1	TTR-Alone	EV	10	HAA	deg	-8.536	-8.185	-8.011	-8.500	-7.961	-8.116
1	TTR-Alone	EV	10	SVA	deg	8.202	8.402	8.385	8.058	7.871	7.797
1	TTR-Alone	EV	700	TAS	mm	-3.847	-3.877	-3.444	-3.523	-3.653	-3.434
1	TTR-Alone	EV	700	CFD	mm	3.120	3.176	2.685	2.946	2.822	2.635
1	TTR-Alone	EV	700	HAA	deg	-6.302	-6.656	-6.103	-5.846	-5.803	-5.603
1	TTR-Alone	EV	700	SVA	deg	9.573	9.498	9.949	9.426	9.715	9.658
1	TTR-Alone	IN	10	TAS	mm	-1.489	-1.348	-1.478	-1.478	-1.027	-1.213
1	TTR-Alone	IN	10	CFD	mm	7.836	7.696	7.762	7.462	7.790	8.043
1	TTR-Alone	IN	10	HAA	deg	-1.235	-1.376	-1.473	-1.446	-0.956	-1.195
1	TTR-Alone	IN	10	SVA	deg	13.537	13.844	13.207	13.797	13.475	14.108
1	TTR-Alone	IN	700	TAS	mm	0.905	0.818	0.755	0.801	1.198	0.627
1	TTR-Alone	IN	700	CFD	mm	8.141	8.076	7.984	8.098	8.280	8.318
1	TTR-Alone	IN	700	HAA	deg	0.536	0.688	0.215	0.772	0.560	0.753
1	TTR-Alone	IN	700	SVA	deg	14.335	14.145	13.987	14.258	14.031	14.745
1	TTR-Alone	PFE	10	TAS	mm	-3.888	-3.914	-4.169	-3.930	-3.798	-4.237
1	TTR-Alone	PFE	10	CFD	mm	4.876	4.555	4.723	4.667	4.840	4.623
1	TTR-Alone	PFE	10	HAA	deg	-8.074	-7.873	-8.448	-8.212	-8.307	-8.030
1	TTR-Alone	PFE	10	SVA	deg	8.245	8.135	8.293	7.807	7.965	8.127
1	TTR-Alone	PFE	700	TAS	mm	-2.961	-3.055	-3.164	-2.579	-3.170	-3.197
1	TTR-Alone	PFE	700	CFD	mm	3.253	3.102	3.160	3.087	2.962	3.392
1	TTR-Alone	PFE	700	HAA	deg	-6.814	-7.508	-7.363	-7.065	-7.540	-7.576
1	TTR-Alone	PFE	700	SVA	deg	7.848	8.442	8.108	8.209	7.753	8.169
1	TTR-Alone	DFE	10	TAS	mm	-3.657	-3.928	-3.582	-3.602	-3.752	-3.837
1	TTR-Alone	DFE	10	CFD	mm	4.271	3.989	4.333	3.931	4.497	3.983
1	TTR-Alone	DFE	10	HAA	deg	-7.650	-8.278	-7.519	-7.811	-8.111	-8.022
1	TTR-Alone	DFE	10	SVA	deg	8.808	8.169	8.534	8.814	8.595	8.531
1	TTR-Alone	DFE	700	TAS	mm	-2.937	-3.082	-2.911	-2.987	-3.055	-3.285
1	TTR-Alone	DFE	700	CFD	mm	3.477	3.489	3.563	3.534	3.385	3.135
1	TTR-Alone	DFE	700	HAA	deg	-6.440	-5.924	-6.104	-6.066	-6.424	-6.225
1	TTR-Alone	DFE	700	SVA	deg	8.976	8.439	8.154	8.565	7.955	8.367
1	TTR-Alone	PFI	10	TAS	mm	-1.546	-1.581	-1.253	-1.838	-1.693	-1.927
1	TTR-Alone	PFI	10	CFD	mm	7.781	7.155	7.553	7.908	7.705	7.350
1	TTR-Alone	PFI	10	HAA	deg	-1.593	-1.449	-1.503	-1.947	-2.178	-2.051
1	TTR-Alone	PFI	10	SVA	deg	13.289	13.251	13.301	13.364	13.041	13.295

1	TTR-Alone	PFI	700	TAS	mm	-0.205	-0.173	-0.101	-0.381	-0.189	-0.318
1	TTR-Alone	PFI	700	CFD	mm	8.419	8.447	8.546	8.456	8.485	8.051
1	TTR-Alone	PFI	700	HAA	deg	-1.670	-1.529	-2.056	-2.015	-1.803	-1.926
1	TTR-Alone	PFI	700	SVA	deg	14.205	14.467	14.461	14.489	14.425	13.979
1	TTR-Alone	DFI	10	TAS	mm	-1.451	-1.330	-1.661	-1.151	-1.310	-1.301
1	TTR-Alone	DFI	10	CFD	mm	7.559	7.902	7.743	8.082	8.120	8.214
1	TTR-Alone	DFI	10	HAA	deg	-1.155	-1.510	-1.165	-0.376	-1.269	-0.871
1	TTR-Alone	DFI	10	SVA	deg	13.665	14.076	13.516	13.901	13.757	14.337
1	TTR-Alone	DFI	700	TAS	mm	-0.468	-0.342	-0.608	-0.433	-0.519	-0.347
1	TTR-Alone	DFI	700	CFD	mm	8.146	8.319	8.529	8.394	8.655	8.579
1	TTR-Alone	DFI	700	HAA	deg	0.152	0.265	0.044	0.795	0.166	0.429
1	TTR-Alone	DFI	700	SVA	deg	14.169	13.918	14.020	14.299	13.899	14.279
1	TTR-Suture	N	10	TAS	mm	-2.649	-2.929	-3.174	-2.913	-2.958	-3.031
1	TTR-Suture	N	10	CFD	mm	6.197	6.037	6.336	6.574	6.266	6.568
1	TTR-Suture	N	10	HAA	deg	-4.494	-5.211	-5.532	-5.182	-5.137	-4.823
1	TTR-Suture	N	10	SVA	deg	10.443	9.931	10.152	10.340	10.795	10.814
1	TTR-Suture	N	10	FAO	percent	4.649	4.538	4.475	4.556	4.547	4.578
1	TTR-Suture	N	700	TAS	mm	-2.076	-2.309	-2.710	-2.424	-2.047	-2.281
1	TTR-Suture	N	700	CFD	mm	6.314	6.010	6.413	6.362	6.465	6.116
1	TTR-Suture	N	700	HAA	deg	-4.703	-5.235	-5.495	-5.174	-4.921	-4.884
1	TTR-Suture	N	700	SVA	deg	10.598	10.228	10.640	10.370	10.467	11.031
1	TTR-Suture	N	700	FAO	percent	4.760	4.730	4.831	4.691	4.743	4.907
1	TTR-Suture	PF	10	TAS	mm	-3.123	-3.137	-3.230	-2.792	-2.793	-3.260
1	TTR-Suture	PF	10	CFD	mm	6.538	6.287	6.382	6.579	6.607	6.339
1	TTR-Suture	PF	10	HAA	deg	-5.426	-5.282	-5.193	-5.495	-5.123	-5.816
1	TTR-Suture	PF	10	SVA	deg	10.094	9.664	10.191	10.019	10.788	10.498
1	TTR-Suture	PF	700	TAS	mm	-2.338	-2.778	-2.247	-2.279	-2.556	-2.496
1	TTR-Suture	PF	700	CFD	mm	6.283	6.935	6.590	6.694	6.562	6.362
1	TTR-Suture	PF	700	HAA	deg	-4.786	-5.490	-4.902	-5.431	-5.016	-5.550
1	TTR-Suture	PF	700	SVA	deg	10.121	9.311	10.034	9.498	9.552	9.287
1	TTR-Suture	DF	10	TAS	mm	-2.784	-3.035	-2.891	-2.918	-2.872	-2.609
1	TTR-Suture	DF	10	CFD	mm	5.719	5.843	5.794	5.998	6.481	6.013
1	TTR-Suture	DF	10	HAA	deg	-4.742	-4.939	-5.169	-4.822	-4.895	-4.517
1	TTR-Suture	DF	10	SVA	deg	11.062	10.728	10.686	11.013	10.996	11.076
1	TTR-Suture	DF	700	TAS	mm	-2.184	-2.256	-2.723	-2.691	-2.536	-2.255
1	TTR-Suture	DF	700	CFD	mm	6.217	6.526	6.289	5.953	6.149	5.983
1	TTR-Suture	DF	700	HAA	deg	-3.701	-3.409	-4.421	-4.775	-4.367	-3.880
1	TTR-Suture	DF	700	SVA	deg	11.313	11.026	10.873	10.592	10.967	11.371
1	TTR-Suture	EV	10	TAS	mm	-4.255	-4.306	-4.303	-4.625	-3.957	-3.987
1	TTR-Suture	EV	10	CFD	mm	4.758	4.492	4.156	4.087	4.444	4.127
1	TTR-Suture	EV	10	HAA	deg	-8.318	-8.619	-8.189	-8.267	-7.991	-8.106
1	TTR-Suture	EV	10	SVA	deg	8.133	8.098	7.873	7.664	8.073	8.257
1	TTR-Suture	EV	700	TAS	mm	-4.260	-4.348	-3.846	-3.855	-4.196	-3.720
1	TTR-Suture	EV	700	CFD	mm	4.446	4.631	4.595	4.449	4.128	4.305
1	TTR-Suture	EV	700	HAA	deg	-7.306	-7.893	-6.718	-6.924	-7.466	-7.154
1	TTR-Suture	EV	700	SVA	deg	8.696	8.501	9.152	8.928	8.728	8.905
1	TTR-Suture	IN	10	TAS	mm	-1.549	-1.246	-1.402	-1.277	-1.384	-1.732
1	TTR-Suture	IN	10	CFD	mm	7.903	8.326	8.276	7.890	8.427	8.132
1	TTR-Suture	IN	10	HAA	deg	-1.642	-1.757	-1.135	-1.245	-1.303	-1.670
1	TTR-Suture	IN	10	SVA	deg	13.565	14.104	13.866	13.799	13.226	13.191
1	TTR-Suture	IN	700	TAS	mm	-0.516	-0.378	-0.552	-0.717	-0.353	-0.739
1	TTR-Suture	IN	700	CFD	mm	8.176	8.421	8.260	7.884	8.302	8.070
1	TTR-Suture	IN	700	HAA	deg	-0.593	-0.295	-0.224	-0.303	-0.068	-0.378
1	TTR-Suture	IN	700	SVA	deg	13.936	14.803	14.628	14.413	14.582	14.357
1	TTR-Suture	PFE	10	TAS	mm	-4.242	-4.526	-4.486	-4.055	-4.344	-4.446
1	TTR-Suture	PFE	10	CFD	mm	4.351	4.358	4.319	4.619	4.300	4.281
1	TTR-Suture	PFE	10	HAA	deg	-8.359	-8.753	-8.646	-7.970	-8.314	-8.007
1	TTR-Suture	PFE	10	SVA	deg	8.010	7.603	7.277	8.169	7.946	8.354
1	TTR-Suture	PFE	700	TAS	mm	-3.544	-4.075	-3.528	-3.710	-3.717	-3.653
1	TTR-Suture	PFE	700	CFD	mm	3.383	3.419	3.414	3.512	3.453	3.271
1	TTR-Suture	PFE	700	HAA	deg	-7.979	-8.570	-8.012	-7.961	-7.993	-8.087
1	TTR-Suture	PFE	700	SVA	deg	8.711	8.702	8.295	8.837	8.655	8.891
1	TTR-Suture	DFE	10	TAS	mm	-3.787	-3.624	-4.047	-4.276	-3.766	-4.301
1	TTR-Suture	DFE	10	CFD	mm	4.248	3.885	4.716	4.037	4.404	4.103
1	TTR-Suture	DFE	10	HAA	deg	-7.865	-7.709	-8.477	-8.250	-7.721	-8.392
1	TTR-Suture	DFE	10	SVA	deg	8.291	8.344	8.460	8.515	8.144	8.048
1	TTR-Suture	DFE	700	TAS	mm	-2.515	-2.730	-3.043	-2.796	-2.573	-2.590
1	TTR-Suture	DFE	700	CFD	mm	4.299	4.042	4.480	4.129	4.228	4.138
1	TTR-Suture	DFE	700	HAA	deg	-6.800	-6.865	-7.607	-7.305	-6.955	-7.347
1	TTR-Suture	DFE	700	SVA	deg	7.869	8.219	7.854	7.847	7.892	8.351

1	TTR-Suture	PFI	10	TAS	mm	-1.553	-1.730	-1.877	-1.726	-2.143	-1.382
1	TTR-Suture	PFI	10	CFD	mm	7.937	7.897	7.607	7.429	7.715	7.818
1	TTR-Suture	PFI	10	HAA	deg	-1.716	-2.233	-2.105	-2.130	-2.097	-1.631
1	TTR-Suture	PFI	10	SVA	deg	13.544	12.960	12.752	13.246	13.385	12.730
1	TTR-Suture	PFI	700	TAS	mm	-0.637	-0.410	-0.700	-0.303	-0.467	-0.081
1	TTR-Suture	PFI	700	CFD	mm	8.829	8.618	8.368	8.850	8.857	8.865
1	TTR-Suture	PFI	700	HAA	deg	-1.648	-1.713	-1.611	-1.771	-1.784	-1.642
1	TTR-Suture	PFI	700	SVA	deg	13.639	13.527	13.629	13.797	13.359	13.181
1	TTR-Suture	DFI	10	TAS	mm	-1.384	-1.402	-1.651	-1.410	-1.570	-1.696
1	TTR-Suture	DFI	10	CFD	mm	8.062	8.281	7.894	8.355	8.226	8.185
1	TTR-Suture	DFI	10	HAA	deg	-1.093	-1.001	-1.909	-1.190	-1.446	-1.405
1	TTR-Suture	DFI	10	SVA	deg	13.899	13.815	13.657	13.270	13.203	13.493
1	TTR-Suture	DFI	700	TAS	mm	-1.390	-1.201	-1.481	-1.116	-1.251	-1.020
1	TTR-Suture	DFI	700	CFD	mm	8.776	9.192	8.909	9.098	8.981	8.989
1	TTR-Suture	DFI	700	HAA	deg	-0.794	-0.790	-1.216	-0.468	-0.832	-0.740
1	TTR-Suture	DFI	700	SVA	deg	13.955	13.360	13.669	13.725	14.045	14.232
2	Intact-Native	N	10	TAS	mm	-1.102	-1.826	-1.386	-1.341	-1.398	-1.513
2	Intact-Native	N	10	CFD	mm	8.032	7.726	8.191	7.621	7.711	7.789
2	Intact-Native	N	10	HAA	deg	-3.007	-3.660	-3.341	-3.351	-3.682	-3.647
2	Intact-Native	N	10	SVA	deg	14.198	13.475	14.019	13.760	13.573	13.959
2	Intact-Native	N	10	FAO	percent	8.798	8.866	8.886	8.824	8.875	8.905
2	Intact-Native	N	700	TAS	mm	-1.037	-1.127	-1.422	-0.976	-0.832	-0.973
2	Intact-Native	N	700	CFD	mm	7.448	7.498	7.639	7.331	7.171	6.903
2	Intact-Native	N	700	HAA	deg	-3.839	-3.557	-4.217	-3.372	-3.454	-3.600
2	Intact-Native	N	700	SVA	deg	12.711	12.640	12.750	12.758	12.813	13.098
2	Intact-Native	N	700	FAO	percent	9.058	9.083	9.006	9.135	9.101	9.089
2	Intact-Native	PF	10	TAS	mm	-1.754	-1.645	-1.711	-1.832	-1.576	-1.379
2	Intact-Native	PF	10	CFD	mm	7.671	7.877	8.290	8.000	7.894	8.118
2	Intact-Native	PF	10	HAA	deg	-3.834	-3.843	-3.745	-4.207	-3.514	-3.160
2	Intact-Native	PF	10	SVA	deg	13.496	13.035	13.042	13.426	14.092	13.738
2	Intact-Native	PF	700	TAS	mm	-1.798	-1.640	-1.782	-1.344	-1.112	-1.379
2	Intact-Native	PF	700	CFD	mm	7.346	7.707	8.091	7.256	7.440	7.929
2	Intact-Native	PF	700	HAA	deg	-3.943	-3.955	-4.373	-3.715	-3.459	-3.938
2	Intact-Native	PF	700	SVA	deg	13.474	13.453	12.967	13.550	13.746	13.067
2	Intact-Native	DF	10	TAS	mm	-1.422	-1.713	-1.246	-1.454	-1.082	-0.819
2	Intact-Native	DF	10	CFD	mm	7.725	7.523	7.811	7.773	7.833	7.626
2	Intact-Native	DF	10	HAA	deg	-3.005	-3.670	-3.262	-3.613	-2.628	-2.623
2	Intact-Native	DF	10	SVA	deg	14.854	14.350	14.269	14.464	14.700	14.396
2	Intact-Native	DF	700	TAS	mm	-1.252	-1.359	-1.156	-1.605	-1.321	-1.540
2	Intact-Native	DF	700	CFD	mm	6.123	6.033	5.912	6.107	6.211	5.994
2	Intact-Native	DF	700	HAA	deg	-3.954	-3.650	-3.896	-4.065	-3.609	-4.170
2	Intact-Native	DF	700	SVA	deg	14.741	14.484	14.232	14.283	14.625	14.167
2	Intact-Native	EV	10	TAS	mm	-2.569	-2.336	-2.413	-2.817	-2.264	-2.488
2	Intact-Native	EV	10	CFD	mm	6.141	6.547	6.869	6.665	6.232	6.105
2	Intact-Native	EV	10	HAA	deg	-6.217	-5.403	-5.591	-6.089	-5.750	-5.762
2	Intact-Native	EV	10	SVA	deg	11.555	12.548	11.323	11.802	11.945	11.923
2	Intact-Native	EV	700	TAS	mm	-3.761	-3.571	-3.679	-3.152	-3.164	-3.526
2	Intact-Native	EV	700	CFD	mm	5.717	6.027	5.630	5.563	5.561	5.540
2	Intact-Native	EV	700	HAA	deg	-6.790	-6.485	-6.473	-6.281	-6.513	-6.290
2	Intact-Native	EV	700	SVA	deg	10.472	11.519	11.380	11.067	10.882	10.985
2	Intact-Native	IN	10	TAS	mm	-0.392	-0.541	-0.325	-0.485	-0.488	-0.315
2	Intact-Native	IN	10	CFD	mm	9.011	9.175	8.959	8.819	8.853	9.116
2	Intact-Native	IN	10	HAA	deg	-0.210	-0.537	-0.431	-0.770	-0.434	-0.804
2	Intact-Native	IN	10	SVA	deg	15.986	16.398	16.270	15.890	15.704	15.937
2	Intact-Native	IN	700	TAS	mm	-0.706	-0.545	-0.362	-0.916	-0.439	-0.752
2	Intact-Native	IN	700	CFD	mm	8.036	8.566	8.501	8.174	8.348	8.282
2	Intact-Native	IN	700	HAA	deg	-0.803	-1.005	-0.823	-1.399	-1.213	-1.279
2	Intact-Native	IN	700	SVA	deg	15.376	15.462	16.002	15.599	15.681	15.685
2	Intact-Native	PFE	10	TAS	mm	-2.913	-2.959	-2.920	-2.846	-2.730	-3.012
2	Intact-Native	PFE	10	CFD	mm	6.541	6.391	6.308	6.329	6.363	6.742
2	Intact-Native	PFE	10	HAA	deg	-6.770	-6.273	-6.775	-6.231	-5.966	-6.248
2	Intact-Native	PFE	10	SVA	deg	11.281	11.543	11.454	11.309	11.468	11.483
2	Intact-Native	PFE	700	TAS	mm	-2.486	-2.732	-2.567	-2.396	-2.333	-2.325
2	Intact-Native	PFE	700	CFD	mm	6.599	6.513	6.671	6.107	6.036	6.372
2	Intact-Native	PFE	700	HAA	deg	-5.893	-6.401	-6.652	-6.130	-5.857	-5.831
2	Intact-Native	PFE	700	SVA	deg	11.500	11.195	11.458	11.301	11.541	11.124
2	Intact-Native	DFE	10	TAS	mm	-2.215	-2.333	-2.509	-2.333	-2.425	-2.442
2	Intact-Native	DFE	10	CFD	mm	5.541	6.167	6.213	6.247	5.889	5.951
2	Intact-Native	DFE	10	HAA	deg	-5.351	-5.660	-6.046	-5.625	-5.872	-6.081
2	Intact-Native	DFE	10	SVA	deg	11.481	11.995	11.705	12.549	12.162	11.754

2	Intact-Native	DFE	700	TAS	mm	-2.765	-2.509	-2.354	-2.661	-2.687	-2.704
2	Intact-Native	DFE	700	CFD	mm	5.343	5.053	5.442	5.615	5.500	5.198
2	Intact-Native	DFE	700	HAA	deg	-5.839	-5.402	-5.646	-5.787	-6.070	-6.184
2	Intact-Native	DFE	700	SVA	deg	10.499	11.211	11.132	10.530	10.992	10.941
2	Intact-Native	PFI	10	TAS	mm	-0.365	-0.527	-0.243	-0.207	-0.288	-0.728
2	Intact-Native	PFI	10	CFD	mm	9.708	9.260	9.220	8.968	9.214	8.822
2	Intact-Native	PFI	10	HAA	deg	-0.320	-0.823	-0.328	-0.292	-0.253	-0.659
2	Intact-Native	PFI	10	SVA	deg	16.289	15.734	16.495	16.203	15.946	15.782
2	Intact-Native	PFI	700	TAS	mm	-0.463	0.028	-0.535	-0.658	-0.172	-0.701
2	Intact-Native	PFI	700	CFD	mm	9.652	9.952	9.387	9.656	9.889	9.676
2	Intact-Native	PFI	700	HAA	deg	-1.581	-0.779	-1.362	-0.970	-0.336	-1.050
2	Intact-Native	PFI	700	SVA	deg	14.876	15.185	15.192	15.489	15.502	15.602
2	Intact-Native	DFI	10	TAS	mm	-0.504	-0.406	0.069	-0.144	0.165	-0.177
2	Intact-Native	DFI	10	CFD	mm	9.215	9.768	9.478	9.678	9.318	9.687
2	Intact-Native	DFI	10	HAA	deg	-0.307	-0.039	-0.040	-0.400	0.645	-0.426
2	Intact-Native	DFI	10	SVA	deg	16.436	16.093	16.328	16.501	16.689	16.465
2	Intact-Native	DFI	700	TAS	mm	0.012	-0.255	0.205	0.364	0.404	-0.031
2	Intact-Native	DFI	700	CFD	mm	9.192	9.383	9.487	9.289	9.185	9.452
2	Intact-Native	DFI	700	HAA	deg	-0.873	-1.357	-0.996	-1.007	-0.494	-0.815
2	Intact-Native	DFI	700	SVA	deg	15.871	15.412	15.598	15.927	16.072	15.711
2	TTR-Alone	N	10	TAS	mm	-1.286	-1.113	-1.076	-1.129	-0.807	-0.871
2	TTR-Alone	N	10	CFD	mm	7.112	7.626	7.619	7.670	7.630	7.539
2	TTR-Alone	N	10	HAA	deg	-3.372	-3.024	-3.220	-2.402	-3.022	-2.648
2	TTR-Alone	N	10	SVA	deg	14.142	14.236	14.321	14.460	14.106	15.167
2	TTR-Alone	N	10	FAO	percent	8.072	8.125	8.102	8.136	8.022	8.155
2	TTR-Alone	N	700	TAS	mm	-0.201	0.189	0.156	0.434	0.249	0.311
2	TTR-Alone	N	700	CFD	mm	6.653	7.190	7.012	7.267	6.793	7.041
2	TTR-Alone	N	700	HAA	deg	-2.776	-2.451	-1.870	-2.382	-2.658	-2.256
2	TTR-Alone	N	700	SVA	deg	14.384	14.844	15.233	14.470	15.149	15.281
2	TTR-Alone	N	700	FAO	percent	8.293	8.422	8.346	8.273	8.345	8.250
2	TTR-Alone	PF	10	TAS	mm	-1.564	-1.407	-1.262	-1.510	-1.216	-1.547
2	TTR-Alone	PF	10	CFD	mm	7.890	8.057	7.874	7.615	7.738	7.875
2	TTR-Alone	PF	10	HAA	deg	-3.348	-3.114	-2.680	-3.620	-2.979	-3.341
2	TTR-Alone	PF	10	SVA	deg	14.104	14.026	14.240	13.771	13.903	13.734
2	TTR-Alone	PF	700	TAS	mm	-0.228	-0.340	0.029	-0.111	-0.334	0.115
2	TTR-Alone	PF	700	CFD	mm	7.242	7.382	6.975	7.204	7.354	7.182
2	TTR-Alone	PF	700	HAA	deg	-2.857	-2.695	-2.544	-2.906	-2.855	-2.817
2	TTR-Alone	PF	700	SVA	deg	13.652	14.095	14.029	14.405	14.070	13.668
2	TTR-Alone	DF	10	TAS	mm	-1.343	-0.811	-1.297	-0.858	-0.976	-0.618
2	TTR-Alone	DF	10	CFD	mm	7.484	7.449	7.409	7.335	7.204	7.572
2	TTR-Alone	DF	10	HAA	deg	-2.725	-2.258	-2.594	-2.707	-2.634	-2.225
2	TTR-Alone	DF	10	SVA	deg	14.490	14.799	14.381	15.171	14.320	14.816
2	TTR-Alone	DF	700	TAS	mm	-0.574	-0.258	-0.198	-0.099	-0.260	-0.343
2	TTR-Alone	DF	700	CFD	mm	6.596	6.997	6.642	6.696	6.450	7.100
2	TTR-Alone	DF	700	HAA	deg	-2.633	-2.008	-1.811	-2.259	-2.472	-2.541
2	TTR-Alone	DF	700	SVA	deg	14.525	14.603	14.036	14.440	14.174	14.368
2	TTR-Alone	EV	10	TAS	mm	-2.076	-1.952	-1.892	-2.256	-2.184	-1.984
2	TTR-Alone	EV	10	CFD	mm	5.904	6.082	6.354	6.315	6.478	6.403
2	TTR-Alone	EV	10	HAA	deg	-5.234	-5.156	-5.056	-5.407	-5.505	-5.441
2	TTR-Alone	EV	10	SVA	deg	12.630	12.137	12.482	12.337	12.548	12.051
2	TTR-Alone	EV	700	TAS	mm	-1.559	-1.408	-1.639	-1.904	-1.701	-1.492
2	TTR-Alone	EV	700	CFD	mm	4.085	4.019	4.225	4.429	4.221	4.165
2	TTR-Alone	EV	700	HAA	deg	-3.555	-3.589	-3.708	-3.553	-3.199	-3.200
2	TTR-Alone	EV	700	SVA	deg	13.268	13.084	13.414	13.369	13.506	13.363
2	TTR-Alone	IN	10	TAS	mm	0.029	0.243	-0.097	-0.061	0.124	-0.076
2	TTR-Alone	IN	10	CFD	mm	8.846	8.890	8.950	8.978	8.915	8.984
2	TTR-Alone	IN	10	HAA	deg	0.035	0.257	-0.121	-0.201	-0.228	0.019
2	TTR-Alone	IN	10	SVA	deg	16.344	16.760	16.467	16.248	16.199	16.308
2	TTR-Alone	IN	700	TAS	mm	2.723	2.432	2.567	2.405	2.747	2.455
2	TTR-Alone	IN	700	CFD	mm	8.137	7.907	7.927	8.249	8.160	8.110
2	TTR-Alone	IN	700	HAA	deg	2.171	2.137	2.295	2.115	2.216	2.454
2	TTR-Alone	IN	700	SVA	deg	17.174	17.900	17.828	17.712	17.873	17.581
2	TTR-Alone	PFE	10	TAS	mm	-2.696	-2.445	-2.282	-2.303	-2.659	-2.624
2	TTR-Alone	PFE	10	CFD	mm	5.746	6.311	5.725	6.083	6.152	6.029
2	TTR-Alone	PFE	10	HAA	deg	-5.955	-5.712	-5.744	-5.918	-5.900	-5.699
2	TTR-Alone	PFE	10	SVA	deg	11.596	11.719	11.696	12.048	11.522	12.285
2	TTR-Alone	PFE	700	TAS	mm	-0.819	-1.143	-0.734	-0.672	-0.920	-0.846
2	TTR-Alone	PFE	700	CFD	mm	4.204	4.086	4.089	3.866	4.145	4.189
2	TTR-Alone	PFE	700	HAA	deg	-6.161	-5.893	-6.222	-6.135	-6.366	-6.028
2	TTR-Alone	PFE	700	SVA	deg	11.296	10.554	10.895	10.698	10.884	11.055

2	TTR-Alone	DFE	10	TAS	mm	-1.996	-1.632	-2.166	-2.087	-2.151	-2.094
2	TTR-Alone	DFE	10	CFD	mm	5.741	6.249	5.971	5.736	6.111	6.123
2	TTR-Alone	DFE	10	HAA	deg	-5.351	-4.848	-5.562	-5.611	-5.224	-5.227
2	TTR-Alone	DFE	10	SVA	deg	11.999	13.128	11.944	12.309	12.534	12.499
2	TTR-Alone	DFE	700	TAS	mm	-1.516	-1.065	-1.351	-0.858	-0.871	-1.193
2	TTR-Alone	DFE	700	CFD	mm	4.083	4.301	4.313	4.246	4.587	4.503
2	TTR-Alone	DFE	700	HAA	deg	-4.960	-4.295	-4.852	-4.147	-4.120	-4.079
2	TTR-Alone	DFE	700	SVA	deg	13.386	13.815	12.888	12.995	13.258	13.278
2	TTR-Alone	PFI	10	TAS	mm	-0.010	-0.310	0.242	-0.245	-0.175	0.002
2	TTR-Alone	PFI	10	CFD	mm	8.860	8.714	9.050	8.656	8.770	9.017
2	TTR-Alone	PFI	10	HAA	deg	0.152	-0.630	-0.019	0.645	-0.199	-0.155
2	TTR-Alone	PFI	10	SVA	deg	16.446	16.462	16.222	16.568	16.729	16.412
2	TTR-Alone	PFI	700	TAS	mm	2.392	1.898	2.035	1.926	1.791	1.937
2	TTR-Alone	PFI	700	CFD	mm	7.454	7.321	7.452	7.464	7.761	7.865
2	TTR-Alone	PFI	700	HAA	deg	1.143	0.121	0.881	1.064	0.261	0.538
2	TTR-Alone	PFI	700	SVA	deg	17.095	16.787	16.797	17.537	17.100	16.948
2	TTR-Alone	DFI	10	TAS	mm	0.280	-0.240	0.441	0.358	0.288	-0.130
2	TTR-Alone	DFI	10	CFD	mm	9.131	9.065	9.060	8.994	8.840	8.926
2	TTR-Alone	DFI	10	HAA	deg	0.360	0.366	0.894	0.237	0.569	-0.603
2	TTR-Alone	DFI	10	SVA	deg	16.625	16.575	16.713	17.284	16.770	16.458
2	TTR-Alone	DFI	700	TAS	mm	1.228	0.756	1.389	1.363	0.937	1.070
2	TTR-Alone	DFI	700	CFD	mm	9.080	8.644	8.714	8.990	8.808	8.625
2	TTR-Alone	DFI	700	HAA	deg	0.752	0.925	1.388	1.285	0.795	0.633
2	TTR-Alone	DFI	700	SVA	deg	17.680	17.826	17.773	17.351	17.422	17.472
2	TTR-Suture	N	10	TAS	mm	-1.061	-1.313	-0.912	-1.326	-1.269	-1.563
2	TTR-Suture	N	10	CFD	mm	7.682	7.710	7.563	7.687	7.714	7.588
2	TTR-Suture	N	10	HAA	deg	-2.326	-3.571	-2.587	-2.992	-3.037	-3.257
2	TTR-Suture	N	10	SVA	deg	14.804	14.360	14.210	14.251	13.626	13.702
2	TTR-Suture	N	10	FAO	percent	10.668	10.827	10.784	10.873	10.825	10.951
2	TTR-Suture	N	700	TAS	mm	0.327	0.396	0.311	0.106	0.095	-0.001
2	TTR-Suture	N	700	CFD	mm	7.090	7.053	7.241	7.141	7.056	7.255
2	TTR-Suture	N	700	HAA	deg	-3.495	-3.825	-3.707	-3.618	-3.735	-3.863
2	TTR-Suture	N	700	SVA	deg	14.256	13.950	13.333	13.855	13.609	13.539
2	TTR-Suture	N	700	FAO	percent	11.075	11.032	11.047	11.042	10.912	11.140
2	TTR-Suture	PF	10	TAS	mm	-1.957	-1.456	-1.463	-1.607	-1.504	-1.647
2	TTR-Suture	PF	10	CFD	mm	7.953	7.930	7.764	7.861	7.786	7.713
2	TTR-Suture	PF	10	HAA	deg	-3.887	-3.638	-3.707	-3.606	-3.684	-3.468
2	TTR-Suture	PF	10	SVA	deg	13.542	13.593	13.336	13.452	13.643	13.504
2	TTR-Suture	PF	700	TAS	mm	-1.404	-0.952	-0.657	-0.902	-1.001	-0.795
2	TTR-Suture	PF	700	CFD	mm	7.427	7.624	7.406	7.549	7.445	7.216
2	TTR-Suture	PF	700	HAA	deg	-4.125	-4.099	-3.980	-4.098	-3.637	-3.702
2	TTR-Suture	PF	700	SVA	deg	15.295	14.560	14.741	14.464	14.682	14.701
2	TTR-Suture	DF	10	TAS	mm	-1.107	-1.380	-1.142	-1.463	-1.044	-1.270
2	TTR-Suture	DF	10	CFD	mm	7.989	7.615	7.432	7.690	7.535	7.120
2	TTR-Suture	DF	10	HAA	deg	-2.740	-3.300	-3.051	-3.121	-2.618	-2.793
2	TTR-Suture	DF	10	SVA	deg	14.647	14.655	14.174	14.105	14.660	14.406
2	TTR-Suture	DF	700	TAS	mm	-0.079	-0.636	-0.307	-0.534	-0.055	-0.124
2	TTR-Suture	DF	700	CFD	mm	8.840	8.882	8.258	8.919	8.923	8.619
2	TTR-Suture	DF	700	HAA	deg	-3.086	-3.533	-3.339	-3.405	-2.668	-3.201
2	TTR-Suture	DF	700	SVA	deg	15.406	15.291	14.973	14.896	14.674	15.225
2	TTR-Suture	EV	10	TAS	mm	-2.109	-2.188	-2.066	-2.141	-2.314	-2.220
2	TTR-Suture	EV	10	CFD	mm	6.526	6.567	6.260	6.367	6.392	6.337
2	TTR-Suture	EV	10	HAA	deg	-5.589	-5.680	-5.221	-5.259	-6.099	-5.334
2	TTR-Suture	EV	10	SVA	deg	12.001	12.462	12.092	12.656	11.619	12.483
2	TTR-Suture	EV	700	TAS	mm	-1.188	-1.282	-0.545	-1.219	-1.224	-0.737
2	TTR-Suture	EV	700	CFD	mm	5.582	5.766	5.319	5.933	5.944	5.644
2	TTR-Suture	EV	700	HAA	deg	-6.037	-5.938	-4.753	-5.473	-5.859	-5.339
2	TTR-Suture	EV	700	SVA	deg	12.393	12.194	12.721	12.713	12.409	13.025
2	TTR-Suture	IN	10	TAS	mm	-0.310	-0.256	-0.073	-0.041	-0.282	-0.178
2	TTR-Suture	IN	10	CFD	mm	9.011	8.522	9.055	9.145	9.065	9.210
2	TTR-Suture	IN	10	HAA	deg	-0.355	-0.599	-0.349	-0.574	-0.860	-0.538
2	TTR-Suture	IN	10	SVA	deg	16.588	16.085	16.394	16.233	16.712	16.385
2	TTR-Suture	IN	700	TAS	mm	0.477	0.370	0.530	0.358	0.430	0.310
2	TTR-Suture	IN	700	CFD	mm	8.217	7.871	7.900	7.686	8.162	7.752
2	TTR-Suture	IN	700	HAA	deg	-0.252	-1.274	-0.959	-0.899	-0.619	-1.065
2	TTR-Suture	IN	700	SVA	deg	15.349	15.243	15.339	15.079	15.684	14.861
2	TTR-Suture	PFE	10	TAS	mm	-2.672	-2.446	-2.664	-2.303	-2.454	-2.113
2	TTR-Suture	PFE	10	CFD	mm	6.424	6.028	6.376	6.179	6.183	6.212
2	TTR-Suture	PFE	10	HAA	deg	-6.208	-5.791	-6.190	-6.256	-6.337	-5.426
2	TTR-Suture	PFE	10	SVA	deg	11.351	11.229	11.504	11.875	11.452	12.316

2	TTR-Suture	PFE	700	TAS	mm	-2.063	-2.196	-2.454	-2.531	-2.338	-2.002
2	TTR-Suture	PFE	700	CFD	mm	5.188	4.928	4.707	5.061	4.827	4.611
2	TTR-Suture	PFE	700	HAA	deg	-6.195	-6.394	-6.876	-6.309	-6.671	-6.046
2	TTR-Suture	PFE	700	SVA	deg	13.070	13.546	13.132	13.568	13.217	13.661
2	TTR-Suture	DFE	10	TAS	mm	-2.211	-2.241	-2.264	-2.212	-2.340	-2.099
2	TTR-Suture	DFE	10	CFD	mm	6.029	6.427	6.316	6.015	6.335	5.949
2	TTR-Suture	DFE	10	HAA	deg	-5.502	-5.229	-5.926	-5.532	-5.533	-5.954
2	TTR-Suture	DFE	10	SVA	deg	11.779	12.174	12.221	12.038	12.637	12.297
2	TTR-Suture	DFE	700	TAS	mm	-1.706	-1.359	-1.418	-1.525	-1.157	-1.198
2	TTR-Suture	DFE	700	CFD	mm	4.588	4.826	4.894	4.606	4.726	4.608
2	TTR-Suture	DFE	700	HAA	deg	-6.000	-5.343	-6.251	-5.907	-5.807	-5.631
2	TTR-Suture	DFE	700	SVA	deg	12.546	12.922	12.741	12.718	13.024	13.026
2	TTR-Suture	PFI	10	TAS	mm	-0.252	-0.497	-0.396	-0.555	-0.413	-0.635
2	TTR-Suture	PFI	10	CFD	mm	9.109	9.015	8.848	9.242	9.481	9.001
2	TTR-Suture	PFI	10	HAA	deg	-0.312	-0.178	-0.271	-0.456	-0.056	-0.090
2	TTR-Suture	PFI	10	SVA	deg	16.220	16.415	16.588	16.493	16.199	16.129
2	TTR-Suture	PFI	700	TAS	mm	0.650	0.267	0.756	0.741	0.652	0.728
2	TTR-Suture	PFI	700	CFD	mm	7.885	7.848	8.264	7.915	8.041	8.004
2	TTR-Suture	PFI	700	HAA	deg	-1.246	-1.323	-0.824	-1.205	-1.093	-1.073
2	TTR-Suture	PFI	700	SVA	deg	16.564	16.459	16.920	16.672	16.881	16.953
2	TTR-Suture	DFI	10	TAS	mm	0.368	0.155	-0.134	-0.080	0.169	-0.065
2	TTR-Suture	DFI	10	CFD	mm	9.366	9.494	9.219	9.225	8.894	8.960
2	TTR-Suture	DFI	10	HAA	deg	0.333	-0.106	0.377	-0.050	0.165	-0.084
2	TTR-Suture	DFI	10	SVA	deg	16.700	16.296	16.401	16.062	16.438	16.852
2	TTR-Suture	DFI	700	TAS	mm	1.401	1.603	1.687	0.785	1.209	1.232
2	TTR-Suture	DFI	700	CFD	mm	8.840	9.116	9.174	8.672	8.845	8.722
2	TTR-Suture	DFI	700	HAA	deg	0.313	0.602	1.240	0.278	0.519	0.410
2	TTR-Suture	DFI	700	SVA	deg	16.944	17.269	16.949	16.588	16.991	16.913
3	Intact-Native	N	10	TAS	mm	-2.558	-2.608	-2.585	-2.431	-3.139	-2.519
3	Intact-Native	N	10	CFD	mm	6.024	5.883	5.434	5.795	5.974	6.168
3	Intact-Native	N	10	HAA	deg	-4.725	-4.936	-4.925	-4.424	-5.455	-4.473
3	Intact-Native	N	10	SVA	deg	12.231	12.249	12.503	12.443	12.380	12.349
3	Intact-Native	N	10	FAO	percent	9.713	9.640	9.665	9.687	9.615	9.702
3	Intact-Native	N	700	TAS	mm	-1.837	-1.729	-1.908	-1.900	-2.328	-1.881
3	Intact-Native	N	700	CFD	mm	5.742	5.804	5.697	5.717	5.495	5.586
3	Intact-Native	N	700	HAA	deg	-3.792	-3.473	-3.747	-3.405	-3.944	-3.555
3	Intact-Native	N	700	SVA	deg	12.595	13.227	12.505	13.183	12.999	13.253
3	Intact-Native	N	700	FAO	percent	9.941	9.915	9.882	9.827	9.963	9.812
3	Intact-Native	PF	10	TAS	mm	-3.067	-2.760	-2.636	-2.492	-2.400	-2.576
3	Intact-Native	PF	10	CFD	mm	5.900	6.124	5.773	6.098	6.141	6.087
3	Intact-Native	PF	10	HAA	deg	-5.619	-5.586	-5.052	-5.170	-5.474	-5.884
3	Intact-Native	PF	10	SVA	deg	11.667	12.279	11.737	12.452	12.138	12.123
3	Intact-Native	PF	700	TAS	mm	-2.062	-2.441	-1.796	-1.839	-1.964	-2.122
3	Intact-Native	PF	700	CFD	mm	6.024	6.237	5.831	6.094	5.835	6.011
3	Intact-Native	PF	700	HAA	deg	-5.023	-5.314	-4.715	-5.018	-5.106	-5.490
3	Intact-Native	PF	700	SVA	deg	13.309	12.814	13.030	12.821	12.684	12.512
3	Intact-Native	DF	10	TAS	mm	-2.618	-2.122	-2.332	-2.734	-2.555	-2.668
3	Intact-Native	DF	10	CFD	mm	5.745	5.879	5.552	5.671	5.564	5.743
3	Intact-Native	DF	10	HAA	deg	-4.773	-4.735	-4.703	-5.031	-4.530	-4.569
3	Intact-Native	DF	10	SVA	deg	13.024	12.644	12.986	12.169	12.563	12.243
3	Intact-Native	DF	700	TAS	mm	-2.080	-1.849	-2.063	-2.054	-1.960	-1.654
3	Intact-Native	DF	700	CFD	mm	5.902	5.227	5.420	5.562	5.405	5.229
3	Intact-Native	DF	700	HAA	deg	-4.634	-4.317	-4.315	-4.733	-4.757	-4.237
3	Intact-Native	DF	700	SVA	deg	13.429	13.302	13.211	13.487	13.198	13.540
3	Intact-Native	EV	10	TAS	mm	-3.551	-3.797	-3.932	-3.690	-3.821	-3.929
3	Intact-Native	EV	10	CFD	mm	4.107	3.994	4.051	4.239	4.025	4.263
3	Intact-Native	EV	10	HAA	deg	-7.260	-7.955	-8.102	-7.917	-7.841	-8.047
3	Intact-Native	EV	10	SVA	deg	10.301	9.746	10.253	9.917	9.915	9.323
3	Intact-Native	EV	700	TAS	mm	-3.043	-3.545	-3.249	-3.444	-3.246	-3.653
3	Intact-Native	EV	700	CFD	mm	4.261	4.396	4.107	4.227	4.093	4.339
3	Intact-Native	EV	700	HAA	deg	-7.194	-8.138	-7.543	-8.244	-7.868	-8.058
3	Intact-Native	EV	700	SVA	deg	9.625	9.657	9.771	10.300	9.894	9.635
3	Intact-Native	IN	10	TAS	mm	-1.425	-1.432	-1.538	-1.239	-1.573	-1.419
3	Intact-Native	IN	10	CFD	mm	7.572	7.289	7.426	7.280	7.408	7.454
3	Intact-Native	IN	10	HAA	deg	-1.890	-1.585	-1.693	-1.329	-1.446	-1.291
3	Intact-Native	IN	10	SVA	deg	14.512	14.566	14.547	15.112	15.038	15.081
3	Intact-Native	IN	700	TAS	mm	-1.744	-1.552	-1.806	-1.601	-1.505	-1.820
3	Intact-Native	IN	700	CFD	mm	6.613	6.381	6.885	6.746	6.600	6.656
3	Intact-Native	IN	700	HAA	deg	-2.114	-1.821	-2.296	-2.497	-1.807	-2.343
3	Intact-Native	IN	700	SVA	deg	14.635	14.497	14.384	14.009	14.332	14.222

3	Intact-Native	PFE	10	TAS	mm	-3.689	-3.591	-3.975	-3.529	-3.806	-3.842
3	Intact-Native	PFE	10	CFD	mm	4.805	4.709	4.778	5.085	4.600	4.543
3	Intact-Native	PFE	10	HAA	deg	-7.336	-7.254	-8.376	-7.635	-7.877	-7.856
3	Intact-Native	PFE	10	SVA	deg	9.990	10.349	9.638	10.395	9.901	9.934
3	Intact-Native	PFE	700	TAS	mm	-2.882	-2.995	-3.115	-2.899	-2.906	-2.740
3	Intact-Native	PFE	700	CFD	mm	5.171	5.142	4.640	5.282	4.735	4.716
3	Intact-Native	PFE	700	HAA	deg	-6.710	-6.943	-7.410	-7.199	-6.812	-6.848
3	Intact-Native	PFE	700	SVA	deg	9.888	10.811	9.969	10.872	10.678	10.689
3	Intact-Native	DFE	10	TAS	mm	-3.620	-3.531	-3.644	-3.390	-3.912	-3.510
3	Intact-Native	DFE	10	CFD	mm	4.355	4.106	4.150	4.666	4.389	4.140
3	Intact-Native	DFE	10	HAA	deg	-7.337	-7.562	-7.246	-7.400	-7.802	-7.313
3	Intact-Native	DFE	10	SVA	deg	10.355	10.326	10.603	10.719	10.229	10.344
3	Intact-Native	DFE	700	TAS	mm	-3.023	-2.773	-3.374	-2.960	-3.194	-2.891
3	Intact-Native	DFE	700	CFD	mm	4.252	4.189	4.267	4.261	4.366	4.092
3	Intact-Native	DFE	700	HAA	deg	-8.265	-7.965	-8.975	-8.664	-8.729	-8.065
3	Intact-Native	DFE	700	SVA	deg	9.815	10.119	9.850	9.978	9.072	9.737
3	Intact-Native	PFI	10	TAS	mm	-1.740	-1.452	-1.519	-1.495	-1.581	-1.638
3	Intact-Native	PFI	10	CFD	mm	6.984	7.081	6.769	7.099	6.905	7.233
3	Intact-Native	PFI	10	HAA	deg	-2.392	-2.524	-2.169	-2.699	-2.810	-1.673
3	Intact-Native	PFI	10	SVA	deg	14.529	14.231	14.600	14.881	13.812	14.607
3	Intact-Native	PFI	700	TAS	mm	-1.139	-0.744	-0.651	-0.928	-0.953	-1.166
3	Intact-Native	PFI	700	CFD	mm	7.611	7.962	7.602	7.595	7.518	7.647
3	Intact-Native	PFI	700	HAA	deg	-2.014	-2.791	-1.814	-2.097	-2.044	-2.180
3	Intact-Native	PFI	700	SVA	deg	14.088	13.616	14.967	14.555	14.131	14.339
3	Intact-Native	DFI	10	TAS	mm	-1.580	-1.468	-1.080	-1.606	-1.538	-1.527
3	Intact-Native	DFI	10	CFD	mm	7.249	7.407	7.585	7.410	7.301	7.369
3	Intact-Native	DFI	10	HAA	deg	-1.984	-1.858	-1.432	-2.093	-1.807	-1.718
3	Intact-Native	DFI	10	SVA	deg	14.689	14.605	14.982	14.455	14.872	14.978
3	Intact-Native	DFI	700	TAS	mm	-1.205	-0.740	-0.801	-0.595	-0.462	-0.785
3	Intact-Native	DFI	700	CFD	mm	7.509	7.888	7.864	7.646	7.597	7.954
3	Intact-Native	DFI	700	HAA	deg	-1.932	-1.336	-1.367	-1.308	-1.269	-1.815
3	Intact-Native	DFI	700	SVA	deg	14.854	15.269	15.616	15.335	15.578	15.213
3	TTR-Alone	N	10	TAS	mm	-2.676	-2.542	-2.189	-1.777	-2.251	-2.191
3	TTR-Alone	N	10	CFD	mm	5.574	5.503	5.444	5.965	5.695	5.820
3	TTR-Alone	N	10	HAA	deg	-5.207	-4.359	-4.495	-3.876	-4.149	-4.594
3	TTR-Alone	N	10	SVA	deg	12.607	12.656	12.905	12.576	12.720	13.076
3	TTR-Alone	N	10	FAO	percent	9.715	9.668	9.677	9.649	9.696	9.601
3	TTR-Alone	N	700	TAS	mm	0.093	0.158	0.791	0.540	0.218	0.294
3	TTR-Alone	N	700	CFD	mm	5.532	6.007	5.511	5.956	5.841	5.611
3	TTR-Alone	N	700	HAA	deg	-4.421	-3.923	-4.096	-3.672	-4.272	-3.870
3	TTR-Alone	N	700	SVA	deg	13.847	13.600	13.684	13.672	13.925	13.996
3	TTR-Alone	N	700	FAO	percent	9.954	9.881	9.902	9.854	9.852	9.886
3	TTR-Alone	PF	10	TAS	mm	-2.448	-2.498	-2.402	-2.130	-2.679	-2.383
3	TTR-Alone	PF	10	CFD	mm	5.916	5.954	5.827	5.871	5.481	5.768
3	TTR-Alone	PF	10	HAA	deg	-4.602	-4.873	-4.271	-4.193	-4.936	-4.668
3	TTR-Alone	PF	10	SVA	deg	12.226	12.603	12.417	12.385	11.961	12.412
3	TTR-Alone	PF	700	TAS	mm	-1.754	-1.752	-1.757	-1.473	-1.807	-1.828
3	TTR-Alone	PF	700	CFD	mm	6.236	6.475	6.084	5.926	5.944	6.107
3	TTR-Alone	PF	700	HAA	deg	-4.387	-4.725	-4.296	-4.658	-4.472	-4.542
3	TTR-Alone	PF	700	SVA	deg	13.523	13.370	13.490	13.298	13.210	13.357
3	TTR-Alone	DF	10	TAS	mm	-2.193	-1.986	-2.106	-2.315	-2.056	-2.049
3	TTR-Alone	DF	10	CFD	mm	5.670	5.427	5.244	5.472	5.387	5.839
3	TTR-Alone	DF	10	HAA	deg	-4.191	-4.151	-4.384	-4.595	-4.117	-4.154
3	TTR-Alone	DF	10	SVA	deg	13.281	12.954	12.768	12.413	13.077	13.078
3	TTR-Alone	DF	700	TAS	mm	-0.873	-0.783	-0.809	-1.050	-0.914	-0.901
3	TTR-Alone	DF	700	CFD	mm	5.108	5.300	5.390	5.046	5.299	5.355
3	TTR-Alone	DF	700	HAA	deg	-2.357	-2.513	-2.324	-2.186	-2.662	-2.182
3	TTR-Alone	DF	700	SVA	deg	13.223	13.260	13.180	12.980	13.861	13.853
3	TTR-Alone	EV	10	TAS	mm	-3.569	-3.605	-3.569	-3.259	-3.018	-3.326
3	TTR-Alone	EV	10	CFD	mm	4.146	3.744	4.083	3.763	3.836	4.270
3	TTR-Alone	EV	10	HAA	deg	-7.659	-7.771	-7.383	-7.377	-7.663	-7.621
3	TTR-Alone	EV	10	SVA	deg	10.632	10.211	10.484	10.448	10.068	10.165
3	TTR-Alone	EV	700	TAS	mm	-2.094	-2.367	-2.439	-2.163	-2.176	-2.199
3	TTR-Alone	EV	700	CFD	mm	1.627	1.604	1.649	1.697	1.747	1.489
3	TTR-Alone	EV	700	HAA	deg	-4.866	-5.426	-5.260	-4.673	-5.514	-4.803
3	TTR-Alone	EV	700	SVA	deg	11.824	11.372	11.342	11.381	11.053	11.368
3	TTR-Alone	IN	10	TAS	mm	-0.758	-1.241	-1.125	-0.441	-1.073	-0.540
3	TTR-Alone	IN	10	CFD	mm	7.368	7.351	6.925	7.336	7.076	7.459
3	TTR-Alone	IN	10	HAA	deg	-1.126	-1.450	-1.561	-0.749	-1.682	-0.639
3	TTR-Alone	IN	10	SVA	deg	15.756	15.040	14.940	15.879	15.360	15.586

3	TTR-Alone	IN	700	TAS	mm	1.630	1.159	1.639	1.180	1.258	1.494
3	TTR-Alone	IN	700	CFD	mm	7.068	6.959	6.795	6.978	6.741	6.840
3	TTR-Alone	IN	700	HAA	deg	0.909	0.629	0.909	1.015	0.377	0.754
3	TTR-Alone	IN	700	SVA	deg	16.116	15.870	16.167	16.447	16.109	16.388
3	TTR-Alone	PFE	10	TAS	mm	-3.264	-3.741	-3.344	-3.480	-3.592	-2.923
3	TTR-Alone	PFE	10	CFD	mm	3.928	4.099	4.114	4.130	4.039	4.559
3	TTR-Alone	PFE	10	HAA	deg	-6.948	-7.127	-6.959	-7.227	-7.209	-6.340
3	TTR-Alone	PFE	10	SVA	deg	10.180	10.521	10.663	9.735	10.325	10.896
3	TTR-Alone	PFE	700	TAS	mm	-2.494	-3.023	-2.795	-2.552	-2.626	-2.460
3	TTR-Alone	PFE	700	CFD	mm	2.420	2.629	2.286	2.357	2.153	2.391
3	TTR-Alone	PFE	700	HAA	deg	-5.036	-5.694	-5.530	-5.675	-5.183	-5.090
3	TTR-Alone	PFE	700	SVA	deg	12.136	12.950	12.264	12.831	12.749	13.073
3	TTR-Alone	DFE	10	TAS	mm	-3.188	-3.258	-3.136	-3.232	-3.213	-3.162
3	TTR-Alone	DFE	10	CFD	mm	3.790	3.520	3.806	4.000	3.869	3.676
3	TTR-Alone	DFE	10	HAA	deg	-6.964	-6.826	-6.445	-6.689	-7.064	-6.605
3	TTR-Alone	DFE	10	SVA	deg	11.129	10.714	10.570	11.149	10.525	10.801
3	TTR-Alone	DFE	700	TAS	mm	-1.329	-1.551	-1.640	-1.933	-1.808	-1.781
3	TTR-Alone	DFE	700	CFD	mm	1.249	0.999	1.128	1.169	1.579	1.076
3	TTR-Alone	DFE	700	HAA	deg	-3.084	-3.542	-3.774	-3.618	-3.711	-3.831
3	TTR-Alone	DFE	700	SVA	deg	13.523	13.497	12.780	13.145	13.222	13.277
3	TTR-Alone	PFI	10	TAS	mm	-1.492	-0.943	-1.466	-1.126	-1.531	-1.170
3	TTR-Alone	PFI	10	CFD	mm	6.963	6.868	6.548	7.152	6.460	6.772
3	TTR-Alone	PFI	10	HAA	deg	-1.596	-1.337	-1.909	-1.860	-2.119	-2.183
3	TTR-Alone	PFI	10	SVA	deg	14.719	14.742	14.793	15.187	14.223	14.561
3	TTR-Alone	PFI	700	TAS	mm	0.074	0.991	0.637	0.552	0.387	0.534
3	TTR-Alone	PFI	700	CFD	mm	6.798	6.748	6.840	7.180	6.767	7.067
3	TTR-Alone	PFI	700	HAA	deg	-1.939	-1.537	-1.700	-1.513	-0.933	-1.566
3	TTR-Alone	PFI	700	SVA	deg	16.639	17.240	17.118	17.681	17.521	17.490
3	TTR-Alone	DFI	10	TAS	mm	-1.150	-0.661	-0.847	-0.985	-1.057	-1.567
3	TTR-Alone	DFI	10	CFD	mm	7.092	6.938	6.998	7.410	6.649	6.848
3	TTR-Alone	DFI	10	HAA	deg	-1.465	-0.962	-1.120	-0.732	-1.261	-2.006
3	TTR-Alone	DFI	10	SVA	deg	15.119	14.997	15.508	15.095	15.169	14.727
3	TTR-Alone	DFI	700	TAS	mm	0.200	0.099	-0.160	0.450	0.330	-0.062
3	TTR-Alone	DFI	700	CFD	mm	7.251	7.235	7.476	7.269	6.956	7.056
3	TTR-Alone	DFI	700	HAA	deg	0.586	-0.008	0.179	0.948	0.570	-0.150
3	TTR-Alone	DFI	700	SVA	deg	16.648	15.809	15.990	16.374	16.952	16.717
3	TTR-Suture	N	10	TAS	mm	-2.326	-2.844	-2.381	-2.573	-2.240	-2.544
3	TTR-Suture	N	10	CFD	mm	5.751	5.520	6.003	5.837	5.899	5.377
3	TTR-Suture	N	10	HAA	deg	-4.672	-5.108	-4.514	-4.744	-4.178	-4.718
3	TTR-Suture	N	10	SVA	deg	12.323	12.364	12.837	12.356	12.332	12.433
3	TTR-Suture	N	10	FAO	percent	8.167	8.180	8.155	7.999	7.937	8.103
3	TTR-Suture	N	700	TAS	mm	-0.109	0.433	-0.066	-0.030	0.090	0.163
3	TTR-Suture	N	700	CFD	mm	5.097	5.308	5.066	5.226	4.918	5.049
3	TTR-Suture	N	700	HAA	deg	-1.802	-1.773	-2.249	-2.342	-2.290	-2.144
3	TTR-Suture	N	700	SVA	deg	12.459	12.968	12.557	11.616	12.461	11.828
3	TTR-Suture	N	700	FAO	percent	8.319	8.284	8.309	8.290	8.336	8.320
3	TTR-Suture	PF	10	TAS	mm	-2.116	-2.455	-2.649	-2.613	-3.049	-2.601
3	TTR-Suture	PF	10	CFD	mm	5.682	5.574	5.900	6.476	6.202	5.778
3	TTR-Suture	PF	10	HAA	deg	-4.508	-4.974	-5.270	-5.170	-5.497	-5.249
3	TTR-Suture	PF	10	SVA	deg	12.285	12.260	12.310	12.120	11.799	11.754
3	TTR-Suture	PF	700	TAS	mm	-1.119	-1.220	-1.447	-1.184	-1.306	-1.156
3	TTR-Suture	PF	700	CFD	mm	4.643	5.038	4.919	5.395	5.096	4.992
3	TTR-Suture	PF	700	HAA	deg	-3.639	-3.199	-3.488	-3.117	-2.867	-2.917
3	TTR-Suture	PF	700	SVA	deg	13.829	14.112	14.627	14.331	14.256	14.324
3	TTR-Suture	DF	10	TAS	mm	-2.058	-2.465	-2.067	-2.385	-2.231	-2.831
3	TTR-Suture	DF	10	CFD	mm	5.713	5.680	5.289	5.584	5.608	5.587
3	TTR-Suture	DF	10	HAA	deg	-3.995	-4.517	-4.477	-4.661	-4.222	-5.028
3	TTR-Suture	DF	10	SVA	deg	13.151	12.595	12.486	12.520	12.762	12.572
3	TTR-Suture	DF	700	TAS	mm	-0.659	-1.185	-1.280	-0.622	-0.391	-0.923
3	TTR-Suture	DF	700	CFD	mm	5.315	5.062	5.028	5.384	4.975	5.109
3	TTR-Suture	DF	700	HAA	deg	-2.631	-3.389	-3.936	-3.231	-3.437	-3.235
3	TTR-Suture	DF	700	SVA	deg	14.714	14.491	14.329	14.008	14.125	14.440
3	TTR-Suture	EV	10	TAS	mm	-3.356	-3.673	-3.630	-3.057	-3.527	-3.974
3	TTR-Suture	EV	10	CFD	mm	3.972	4.146	3.831	3.946	4.344	3.993
3	TTR-Suture	EV	10	HAA	deg	-7.622	-8.051	-7.945	-7.442	-7.856	-8.027
3	TTR-Suture	EV	10	SVA	deg	10.481	10.186	9.857	10.114	9.968	9.748
3	TTR-Suture	EV	700	TAS	mm	-2.132	-2.116	-2.143	-2.062	-1.904	-2.435
3	TTR-Suture	EV	700	CFD	mm	2.361	2.783	2.690	2.547	2.466	2.730
3	TTR-Suture	EV	700	HAA	deg	-5.462	-5.486	-5.216	-5.388	-5.295	-5.628
3	TTR-Suture	EV	700	SVA	deg	11.307	10.861	10.937	11.117	11.003	11.211

3	TTR-Suture	IN	10	TAS	mm	-1.165	-1.422	-1.230	-1.201	-1.265	-1.246
3	TTR-Suture	IN	10	CFD	mm	7.243	7.297	7.304	7.076	6.838	7.140
3	TTR-Suture	IN	10	HAA	deg	-1.183	-1.586	-1.113	-1.714	-1.353	-1.283
3	TTR-Suture	IN	10	SVA	deg	15.534	14.969	15.383	14.698	15.013	15.106
3	TTR-Suture	IN	700	TAS	mm	-0.233	-0.054	-0.120	-0.061	0.202	0.082
3	TTR-Suture	IN	700	CFD	mm	6.201	6.718	6.270	6.507	6.444	6.364
3	TTR-Suture	IN	700	HAA	deg	-0.064	-0.409	-0.228	0.032	0.374	0.104
3	TTR-Suture	IN	700	SVA	deg	17.219	16.440	16.813	17.178	17.635	16.921
3	TTR-Suture	PFE	10	TAS	mm	-3.589	-3.611	-3.632	-3.786	-3.453	-3.605
3	TTR-Suture	PFE	10	CFD	mm	4.689	4.530	4.740	4.457	4.814	4.527
3	TTR-Suture	PFE	10	HAA	deg	-7.597	-7.497	-7.336	-7.741	-7.587	-7.211
3	TTR-Suture	PFE	10	SVA	deg	10.733	10.532	10.506	9.767	10.133	10.344
3	TTR-Suture	PFE	700	TAS	mm	-2.051	-2.053	-2.113	-2.090	-2.194	-2.048
3	TTR-Suture	PFE	700	CFD	mm	4.178	3.817	4.146	4.182	4.162	3.981
3	TTR-Suture	PFE	700	HAA	deg	-6.299	-6.504	-6.508	-6.636	-6.353	-6.158
3	TTR-Suture	PFE	700	SVA	deg	10.067	10.252	10.222	10.104	9.780	10.316
3	TTR-Suture	DFE	10	TAS	mm	-2.806	-3.725	-3.494	-3.531	-3.241	-3.316
3	TTR-Suture	DFE	10	CFD	mm	3.816	4.224	4.411	3.940	4.412	3.931
3	TTR-Suture	DFE	10	HAA	deg	-6.608	-7.590	-7.344	-7.864	-7.018	-7.500
3	TTR-Suture	DFE	10	SVA	deg	10.664	10.150	10.732	10.352	10.742	10.599
3	TTR-Suture	DFE	700	TAS	mm	-1.229	-1.056	-1.707	-1.547	-1.293	-1.622
3	TTR-Suture	DFE	700	CFD	mm	2.406	2.812	2.928	2.785	3.027	2.729
3	TTR-Suture	DFE	700	HAA	deg	-4.647	-4.345	-4.927	-5.203	-4.780	-5.084
3	TTR-Suture	DFE	700	SVA	deg	11.731	12.169	11.625	11.303	11.590	11.380
3	TTR-Suture	PFI	10	TAS	mm	-1.203	-1.217	-1.466	-1.535	-1.853	-1.217
3	TTR-Suture	PFI	10	CFD	mm	7.220	6.898	6.788	7.058	6.858	7.234
3	TTR-Suture	PFI	10	HAA	deg	-1.735	-2.077	-1.924	-2.411	-2.421	-1.600
3	TTR-Suture	PFI	10	SVA	deg	14.156	14.596	14.460	14.853	14.516	15.001
3	TTR-Suture	PFI	700	TAS	mm	0.633	0.383	0.308	0.775	0.506	0.653
3	TTR-Suture	PFI	700	CFD	mm	7.819	7.288	7.411	7.510	7.200	7.613
3	TTR-Suture	PFI	700	HAA	deg	-0.431	-0.859	-0.403	-0.881	-1.392	-0.677
3	TTR-Suture	PFI	700	SVA	deg	15.014	15.234	14.735	14.998	14.469	14.946
3	TTR-Suture	DFI	10	TAS	mm	-0.959	-1.375	-1.224	-1.033	-1.088	-1.306
3	TTR-Suture	DFI	10	CFD	mm	7.702	7.418	7.247	7.351	7.454	7.434
3	TTR-Suture	DFI	10	HAA	deg	-1.559	-1.616	-1.770	-1.790	-1.014	-1.628
3	TTR-Suture	DFI	10	SVA	deg	15.165	14.908	15.200	14.655	15.301	14.430
3	TTR-Suture	DFI	700	TAS	mm	0.437	-0.283	-0.095	0.350	0.072	0.060
3	TTR-Suture	DFI	700	CFD	mm	7.634	7.753	7.666	7.678	7.991	7.700
3	TTR-Suture	DFI	700	HAA	deg	0.120	-0.630	-0.402	-0.590	-0.437	-0.392
3	TTR-Suture	DFI	700	SVA	deg	15.899	16.328	16.022	16.357	16.299	15.550
4	Intact-Native	N	10	TAS	mm	-1.327	-0.873	-0.792	-0.807	-0.837	-1.037
4	Intact-Native	N	10	CFD	mm	6.771	6.889	6.839	7.407	7.442	7.179
4	Intact-Native	N	10	HAA	deg	-1.372	-1.074	-0.998	-0.944	-1.010	-0.552
4	Intact-Native	N	10	SVA	deg	13.934	15.045	14.749	14.562	14.779	14.732
4	Intact-Native	N	10	FAO	percent	13.270	13.041	13.114	13.190	13.243	13.174
4	Intact-Native	N	700	TAS	mm	-0.682	-0.674	-0.579	-0.524	-0.374	-0.918
4	Intact-Native	N	700	CFD	mm	6.618	6.512	6.461	6.515	6.503	6.591
4	Intact-Native	N	700	HAA	deg	-1.337	-1.254	-1.370	-1.304	-1.356	-1.819
4	Intact-Native	N	700	SVA	deg	13.238	13.388	13.480	13.499	13.415	13.245
4	Intact-Native	N	700	FAO	percent	13.354	13.455	13.371	13.296	13.463	13.414
4	Intact-Native	PF	10	TAS	mm	-1.270	-1.210	-1.378	-0.849	-0.958	-1.040
4	Intact-Native	PF	10	CFD	mm	7.203	7.239	7.297	6.960	7.202	7.277
4	Intact-Native	PF	10	HAA	deg	-1.585	-1.440	-1.335	-1.287	-1.416	-1.698
4	Intact-Native	PF	10	SVA	deg	14.106	14.476	13.933	14.243	14.392	13.503
4	Intact-Native	PF	700	TAS	mm	-0.721	-0.448	-0.612	-0.594	-0.615	-0.819
4	Intact-Native	PF	700	CFD	mm	7.227	7.180	6.981	7.060	7.027	7.043
4	Intact-Native	PF	700	HAA	deg	-1.364	-1.198	-1.017	-1.330	-1.260	-1.371
4	Intact-Native	PF	700	SVA	deg	14.868	15.115	14.610	14.423	14.582	14.321
4	Intact-Native	DF	10	TAS	mm	-1.088	-0.971	-0.719	-0.696	-0.586	-0.927
4	Intact-Native	DF	10	CFD	mm	6.857	6.766	6.891	7.177	7.075	6.920
4	Intact-Native	DF	10	HAA	deg	-1.149	-0.701	-1.060	-0.639	-0.572	-0.785
4	Intact-Native	DF	10	SVA	deg	15.309	14.689	15.429	14.944	15.417	14.897
4	Intact-Native	DF	700	TAS	mm	-0.054	0.156	-0.321	0.053	0.024	0.034
4	Intact-Native	DF	700	CFD	mm	7.041	7.332	7.384	7.377	7.291	7.231
4	Intact-Native	DF	700	HAA	deg	-0.676	-0.141	-0.684	0.037	-0.374	-0.677
4	Intact-Native	DF	700	SVA	deg	15.984	16.228	15.689	15.799	16.195	16.177
4	Intact-Native	EV	10	TAS	mm	-1.985	-1.780	-1.780	-1.782	-1.813	-2.207
4	Intact-Native	EV	10	CFD	mm	5.705	5.922	5.938	5.610	6.155	5.598
4	Intact-Native	EV	10	HAA	deg	-3.557	-3.458	-3.347	-3.200	-3.264	-3.595
4	Intact-Native	EV	10	SVA	deg	12.849	13.288	13.500	12.416	12.846	12.834

4	Intact-Native	EV	700	TAS	mm	-1.424	-0.928	-0.992	-1.044	-1.303	-1.320
4	Intact-Native	EV	700	CFD	mm	5.943	6.293	5.849	6.550	6.254	6.148
4	Intact-Native	EV	700	HAA	deg	-3.401	-3.006	-2.919	-3.264	-3.574	-3.213
4	Intact-Native	EV	700	SVA	deg	12.257	12.817	12.156	12.541	12.489	12.676
4	Intact-Native	IN	10	TAS	mm	-0.154	0.019	0.165	0.164	0.182	0.284
4	Intact-Native	IN	10	CFD	mm	8.205	8.473	8.447	8.493	8.435	8.529
4	Intact-Native	IN	10	HAA	deg	0.693	1.144	1.622	1.660	2.100	1.410
4	Intact-Native	IN	10	SVA	deg	16.500	16.199	16.707	16.145	16.508	16.815
4	Intact-Native	IN	700	TAS	mm	0.158	-0.060	0.144	0.633	0.135	0.406
4	Intact-Native	IN	700	CFD	mm	8.449	8.302	8.730	8.708	8.403	8.557
4	Intact-Native	IN	700	HAA	deg	1.184	1.220	1.413	1.590	1.465	1.017
4	Intact-Native	IN	700	SVA	deg	17.227	16.395	16.939	16.485	16.453	16.541
4	Intact-Native	PFE	10	TAS	mm	-1.694	-1.851	-2.269	-1.554	-2.068	-2.110
4	Intact-Native	PFE	10	CFD	mm	5.846	5.303	5.749	5.654	5.457	6.061
4	Intact-Native	PFE	10	HAA	deg	-3.886	-3.616	-4.204	-3.210	-3.761	-4.158
4	Intact-Native	PFE	10	SVA	deg	12.535	12.714	12.719	12.287	12.455	11.921
4	Intact-Native	PFE	700	TAS	mm	-1.317	-1.331	-1.836	-1.134	-1.433	-1.146
4	Intact-Native	PFE	700	CFD	mm	5.776	5.494	5.773	5.516	5.710	5.949
4	Intact-Native	PFE	700	HAA	deg	-3.843	-3.383	-3.533	-2.898	-3.455	-3.229
4	Intact-Native	PFE	700	SVA	deg	12.715	12.628	12.054	12.535	12.898	12.439
4	Intact-Native	DFE	10	TAS	mm	-2.015	-1.637	-1.833	-1.624	-1.735	-1.628
4	Intact-Native	DFE	10	CFD	mm	5.645	5.763	5.560	5.577	5.847	5.265
4	Intact-Native	DFE	10	HAA	deg	-3.583	-3.365	-3.413	-3.252	-3.342	-3.260
4	Intact-Native	DFE	10	SVA	deg	13.067	12.638	12.546	12.605	12.613	12.737
4	Intact-Native	DFE	700	TAS	mm	-1.962	-1.859	-1.960	-1.269	-1.757	-1.710
4	Intact-Native	DFE	700	CFD	mm	5.238	4.983	5.063	5.184	5.335	4.988
4	Intact-Native	DFE	700	HAA	deg	-3.847	-4.289	-4.220	-3.497	-4.109	-4.176
4	Intact-Native	DFE	700	SVA	deg	11.340	11.068	10.858	11.773	10.932	11.898
4	Intact-Native	PFI	10	TAS	mm	-0.008	0.182	0.060	-0.293	0.070	-0.074
4	Intact-Native	PFI	10	CFD	mm	8.269	8.243	8.202	8.346	8.385	7.930
4	Intact-Native	PFI	10	HAA	deg	1.385	1.771	1.577	1.083	1.408	1.003
4	Intact-Native	PFI	10	SVA	deg	16.508	16.651	16.603	16.411	16.653	16.482
4	Intact-Native	PFI	700	TAS	mm	0.419	0.713	0.586	-0.170	-0.007	0.889
4	Intact-Native	PFI	700	CFD	mm	8.688	8.886	8.351	8.552	8.626	8.033
4	Intact-Native	PFI	700	HAA	deg	1.013	1.585	1.757	0.629	0.880	1.464
4	Intact-Native	PFI	700	SVA	deg	16.163	16.839	16.794	15.978	16.068	16.737
4	Intact-Native	DFI	10	TAS	mm	0.278	0.594	0.559	-0.005	0.108	0.070
4	Intact-Native	DFI	10	CFD	mm	8.146	8.410	8.738	8.292	8.478	8.930
4	Intact-Native	DFI	10	HAA	deg	1.589	1.929	1.913	1.401	1.741	1.613
4	Intact-Native	DFI	10	SVA	deg	16.733	16.461	16.796	16.971	16.921	16.506
4	Intact-Native	DFI	700	TAS	mm	0.306	0.368	0.349	0.409	0.569	0.455
4	Intact-Native	DFI	700	CFD	mm	9.351	9.188	9.296	9.345	9.425	9.261
4	Intact-Native	DFI	700	HAA	deg	0.640	0.596	0.450	0.324	0.602	0.308
4	Intact-Native	DFI	700	SVA	deg	15.864	15.928	15.683	15.327	15.788	15.470
4	TTR-Alone	N	10	TAS	mm	-0.712	-0.782	-0.235	-0.906	-0.631	-0.255
4	TTR-Alone	N	10	CFD	mm	6.499	6.627	7.138	6.719	6.848	6.959
4	TTR-Alone	N	10	HAA	deg	-1.016	-1.103	-0.182	-0.791	-0.443	-0.324
4	TTR-Alone	N	10	SVA	deg	15.041	14.636	14.967	14.597	15.375	15.262
4	TTR-Alone	N	10	FAO	percent	12.986	12.900	12.862	12.937	12.938	12.929
4	TTR-Alone	N	700	TAS	mm	-0.245	-0.168	0.075	-0.283	-0.269	0.289
4	TTR-Alone	N	700	CFD	mm	6.539	6.859	6.989	6.736	6.510	7.115
4	TTR-Alone	N	700	HAA	deg	-0.976	-0.647	-0.282	-0.327	-0.460	-0.604
4	TTR-Alone	N	700	SVA	deg	14.822	13.901	14.770	14.721	14.717	14.487
4	TTR-Alone	N	700	FAO	percent	13.090	13.157	13.234	13.162	13.076	13.150
4	TTR-Alone	PF	10	TAS	mm	-0.727	-0.966	-0.365	-0.541	-0.739	-0.782
4	TTR-Alone	PF	10	CFD	mm	7.195	6.880	6.849	6.905	7.251	7.017
4	TTR-Alone	PF	10	HAA	deg	-0.853	-0.663	-0.436	-0.982	-1.512	-1.482
4	TTR-Alone	PF	10	SVA	deg	14.652	14.868	14.387	14.345	14.707	14.634
4	TTR-Alone	PF	700	TAS	mm	0.403	-0.471	0.246	0.375	-0.084	0.034
4	TTR-Alone	PF	700	CFD	mm	6.976	6.607	6.522	6.386	6.557	6.332
4	TTR-Alone	PF	700	HAA	deg	-0.218	-0.527	0.164	-0.009	-0.378	-0.419
4	TTR-Alone	PF	700	SVA	deg	14.441	14.505	14.581	14.588	14.613	14.420
4	TTR-Alone	DF	10	TAS	mm	-0.325	-0.478	-0.564	-0.093	0.170	-0.464
4	TTR-Alone	DF	10	CFD	mm	6.776	6.583	6.259	6.456	6.719	6.658
4	TTR-Alone	DF	10	HAA	deg	-0.195	-0.040	-0.613	-0.218	0.145	-0.551
4	TTR-Alone	DF	10	SVA	deg	15.412	15.110	15.058	15.576	15.810	15.658
4	TTR-Alone	DF	700	TAS	mm	0.679	0.729	0.831	0.671	0.802	0.582
4	TTR-Alone	DF	700	CFD	mm	7.175	7.125	6.954	7.037	6.907	6.861
4	TTR-Alone	DF	700	HAA	deg	-0.267	0.194	-0.156	-0.313	-0.123	-0.639
4	TTR-Alone	DF	700	SVA	deg	15.399	15.253	15.030	14.881	15.307	14.948

4	TTR-Alone	EV	10	TAS	mm	-1.211	-1.300	-1.683	-1.432	-1.974	-1.299
4	TTR-Alone	EV	10	CFD	mm	5.806	5.414	5.321	5.636	5.355	5.451
4	TTR-Alone	EV	10	HAA	deg	-2.798	-2.975	-2.999	-2.730	-2.742	-2.561
4	TTR-Alone	EV	10	SVA	deg	13.282	13.602	13.226	13.141	13.193	12.840
4	TTR-Alone	EV	700	TAS	mm	-0.979	-0.882	-1.549	-1.165	-1.389	-0.927
4	TTR-Alone	EV	700	CFD	mm	4.305	4.190	4.237	3.982	4.218	4.139
4	TTR-Alone	EV	700	HAA	deg	-1.577	-1.373	-1.709	-1.504	-1.775	-1.330
4	TTR-Alone	EV	700	SVA	deg	13.155	13.114	13.281	12.827	13.035	12.846
4	TTR-Alone	IN	10	TAS	mm	0.540	0.105	0.300	0.345	0.287	0.193
4	TTR-Alone	IN	10	CFD	mm	7.945	8.191	8.366	8.038	7.984	7.921
4	TTR-Alone	IN	10	HAA	deg	2.503	2.261	2.041	1.836	1.401	1.802
4	TTR-Alone	IN	10	SVA	deg	17.434	16.844	16.450	16.943	16.569	17.242
4	TTR-Alone	IN	700	TAS	mm	2.464	1.915	2.435	2.504	2.575	2.890
4	TTR-Alone	IN	700	CFD	mm	8.341	7.986	8.458	8.406	8.222	8.109
4	TTR-Alone	IN	700	HAA	deg	2.570	2.202	2.925	2.864	2.918	3.312
4	TTR-Alone	IN	700	SVA	deg	17.782	16.943	17.557	17.368	17.269	17.823
4	TTR-Alone	PFE	10	TAS	mm	-1.791	-1.484	-1.794	-1.660	-1.278	-1.529
4	TTR-Alone	PFE	10	CFD	mm	5.809	6.013	5.156	5.686	5.435	5.741
4	TTR-Alone	PFE	10	HAA	deg	-3.147	-3.569	-3.302	-3.421	-2.972	-3.300
4	TTR-Alone	PFE	10	SVA	deg	13.623	13.163	13.522	12.676	13.197	12.589
4	TTR-Alone	PFE	700	TAS	mm	-0.918	-0.953	-0.801	-1.064	-0.724	-0.705
4	TTR-Alone	PFE	700	CFD	mm	4.690	4.749	4.334	4.723	4.678	4.561
4	TTR-Alone	PFE	700	HAA	deg	-4.697	-4.899	-4.250	-4.948	-4.881	-5.035
4	TTR-Alone	PFE	700	SVA	deg	14.325	14.165	14.483	14.804	14.830	15.137
4	TTR-Alone	DFE	10	TAS	mm	-1.226	-1.447	-1.224	-1.400	-1.481	-1.307
4	TTR-Alone	DFE	10	CFD	mm	5.188	5.269	5.089	5.152	5.152	4.847
4	TTR-Alone	DFE	10	HAA	deg	-2.426	-2.660	-2.714	-2.265	-3.000	-3.090
4	TTR-Alone	DFE	10	SVA	deg	13.389	13.475	13.521	13.665	12.556	13.330
4	TTR-Alone	DFE	700	TAS	mm	-0.892	-0.865	-0.602	-0.388	-0.352	-0.481
4	TTR-Alone	DFE	700	CFD	mm	3.742	3.737	3.690	3.412	3.566	3.732
4	TTR-Alone	DFE	700	HAA	deg	-3.039	-3.518	-3.183	-2.785	-2.886	-3.170
4	TTR-Alone	DFE	700	SVA	deg	14.501	14.511	13.833	14.576	14.016	15.436
4	TTR-Alone	PFI	10	TAS	mm	-0.003	0.361	0.907	0.548	0.335	0.129
4	TTR-Alone	PFI	10	CFD	mm	8.093	7.601	7.689	8.038	7.972	7.836
4	TTR-Alone	PFI	10	HAA	deg	1.769	1.544	2.596	1.834	1.986	1.710
4	TTR-Alone	PFI	10	SVA	deg	16.777	16.562	17.036	16.635	17.134	17.396
4	TTR-Alone	PFI	700	TAS	mm	1.727	2.121	2.081	1.791	1.612	1.867
4	TTR-Alone	PFI	700	CFD	mm	9.048	8.941	9.057	8.839	9.096	9.107
4	TTR-Alone	PFI	700	HAA	deg	1.614	1.486	1.590	1.515	1.417	1.193
4	TTR-Alone	PFI	700	SVA	deg	17.304	16.905	17.277	17.183	16.872	17.381
4	TTR-Alone	DFI	10	TAS	mm	0.610	1.076	0.771	0.721	0.658	0.488
4	TTR-Alone	DFI	10	CFD	mm	8.274	7.884	8.358	8.285	8.274	8.371
4	TTR-Alone	DFI	10	HAA	deg	2.309	2.692	1.987	1.953	2.636	2.009
4	TTR-Alone	DFI	10	SVA	deg	17.479	17.697	17.162	16.327	17.559	17.172
4	TTR-Alone	DFI	700	TAS	mm	1.414	1.669	1.665	1.579	1.716	1.329
4	TTR-Alone	DFI	700	CFD	mm	8.885	8.875	9.222	9.214	9.161	9.060
4	TTR-Alone	DFI	700	HAA	deg	2.141	2.243	2.674	2.762	3.098	2.474
4	TTR-Alone	DFI	700	SVA	deg	17.756	17.891	17.654	17.319	17.885	18.112
4	TTR-Suture	N	10	TAS	mm	-0.440	-0.669	-0.510	-0.695	-0.691	-0.846
4	TTR-Suture	N	10	CFD	mm	7.006	7.121	6.711	6.594	6.966	7.038
4	TTR-Suture	N	10	HAA	deg	-0.658	-0.588	-1.045	-1.066	-0.748	-0.969
4	TTR-Suture	N	10	SVA	deg	15.218	14.638	14.996	14.572	14.915	15.043
4	TTR-Suture	N	10	FAO	percent	9.280	9.166	9.207	9.236	9.338	9.101
4	TTR-Suture	N	700	TAS	mm	-0.293	-0.141	-0.529	-0.241	-0.078	-0.527
4	TTR-Suture	N	700	CFD	mm	7.054	7.309	7.115	6.914	7.533	7.064
4	TTR-Suture	N	700	HAA	deg	-1.157	-0.714	-1.390	-1.080	-0.863	-1.087
4	TTR-Suture	N	700	SVA	deg	14.975	14.999	14.964	14.770	15.316	14.987
4	TTR-Suture	N	700	FAO	percent	9.473	9.405	9.483	9.437	9.401	9.448
4	TTR-Suture	PF	10	TAS	mm	-0.938	-1.101	-1.578	-0.866	-0.962	-0.923
4	TTR-Suture	PF	10	CFD	mm	7.391	7.076	7.117	7.117	7.154	7.182
4	TTR-Suture	PF	10	HAA	deg	-1.021	-1.135	-1.498	-1.023	-0.971	-1.300
4	TTR-Suture	PF	10	SVA	deg	14.184	14.541	14.555	14.806	14.573	14.227
4	TTR-Suture	PF	700	TAS	mm	-0.598	-0.530	-0.772	-0.374	-0.894	-0.396
4	TTR-Suture	PF	700	CFD	mm	6.887	6.498	6.601	6.521	6.795	6.732
4	TTR-Suture	PF	700	HAA	deg	-1.364	-1.203	-1.666	-1.568	-1.539	-1.715
4	TTR-Suture	PF	700	SVA	deg	13.644	13.580	13.894	14.461	13.547	13.942
4	TTR-Suture	DF	10	TAS	mm	-0.055	-0.649	-0.675	-0.773	0.027	-0.307
4	TTR-Suture	DF	10	CFD	mm	6.861	6.502	6.705	6.806	6.887	6.716
4	TTR-Suture	DF	10	HAA	deg	-0.421	-1.107	-0.677	-0.961	0.071	-0.567
4	TTR-Suture	DF	10	SVA	deg	15.530	14.603	15.099	14.686	15.723	15.372

4	TTR-Suture	DF	700	TAS	mm	-0.163	-0.216	-0.251	-0.488	-0.149	-0.361
4	TTR-Suture	DF	700	CFD	mm	6.821	6.530	6.710	6.691	6.872	6.464
4	TTR-Suture	DF	700	HAA	deg	-0.394	-0.345	-0.317	-1.254	-0.448	-0.913
4	TTR-Suture	DF	700	SVA	deg	14.309	13.757	14.462	13.664	14.313	13.934
4	TTR-Suture	EV	10	TAS	mm	-1.119	-1.960	-1.521	-1.743	-1.635	-1.419
4	TTR-Suture	EV	10	CFD	mm	5.599	6.008	5.681	5.603	5.468	5.891
4	TTR-Suture	EV	10	HAA	deg	-3.232	-3.850	-3.239	-3.009	-3.117	-2.829
4	TTR-Suture	EV	10	SVA	deg	13.563	13.029	12.906	12.969	12.984	13.007
4	TTR-Suture	EV	700	TAS	mm	-1.201	-1.398	-1.570	-1.795	-1.760	-1.508
4	TTR-Suture	EV	700	CFD	mm	6.241	6.374	6.006	6.168	5.853	6.253
4	TTR-Suture	EV	700	HAA	deg	-3.169	-3.169	-3.486	-3.456	-3.537	-3.252
4	TTR-Suture	EV	700	SVA	deg	13.903	14.035	13.572	13.690	13.983	14.172
4	TTR-Suture	IN	10	TAS	mm	0.303	0.473	-0.209	0.051	0.562	0.237
4	TTR-Suture	IN	10	CFD	mm	8.241	8.254	8.100	7.885	7.933	8.076
4	TTR-Suture	IN	10	HAA	deg	1.298	1.711	1.033	1.447	1.935	1.499
4	TTR-Suture	IN	10	SVA	deg	16.856	16.757	16.704	16.738	16.509	16.476
4	TTR-Suture	IN	700	TAS	mm	1.166	0.800	0.433	0.417	0.708	1.009
4	TTR-Suture	IN	700	CFD	mm	8.008	7.951	8.061	7.743	7.970	8.181
4	TTR-Suture	IN	700	HAA	deg	1.400	1.591	1.046	1.000	1.229	1.690
4	TTR-Suture	IN	700	SVA	deg	15.930	16.260	15.589	16.425	16.222	16.139
4	TTR-Suture	PFE	10	TAS	mm	-1.659	-1.777	-1.943	-1.645	-1.457	-1.449
4	TTR-Suture	PFE	10	CFD	mm	5.859	5.521	5.941	5.734	5.801	5.455
4	TTR-Suture	PFE	10	HAA	deg	-3.714	-3.583	-3.790	-3.139	-3.587	-3.061
4	TTR-Suture	PFE	10	SVA	deg	12.518	12.559	12.710	12.534	12.419	12.743
4	TTR-Suture	PFE	700	TAS	mm	-0.908	-1.126	-1.309	-0.952	-0.959	-0.878
4	TTR-Suture	PFE	700	CFD	mm	5.306	5.224	5.367	5.696	5.624	5.462
4	TTR-Suture	PFE	700	HAA	deg	-4.505	-4.571	-4.695	-4.054	-4.447	-4.030
4	TTR-Suture	PFE	700	SVA	deg	10.725	10.484	10.662	10.814	10.668	11.153
4	TTR-Suture	DFE	10	TAS	mm	-1.653	-2.224	-1.949	-1.386	-1.555	-1.475
4	TTR-Suture	DFE	10	CFD	mm	5.682	5.738	5.524	5.283	5.136	5.621
4	TTR-Suture	DFE	10	HAA	deg	-3.069	-3.598	-3.354	-3.183	-3.002	-3.039
4	TTR-Suture	DFE	10	SVA	deg	13.109	12.876	12.637	13.142	13.281	12.971
4	TTR-Suture	DFE	700	TAS	mm	-0.839	-1.295	-1.149	-1.243	-1.297	-0.937
4	TTR-Suture	DFE	700	CFD	mm	5.352	5.231	5.386	5.461	5.020	5.330
4	TTR-Suture	DFE	700	HAA	deg	-2.882	-3.295	-3.357	-3.590	-3.183	-3.019
4	TTR-Suture	DFE	700	SVA	deg	12.865	12.675	12.669	12.411	12.524	12.356
4	TTR-Suture	PFI	10	TAS	mm	-0.037	0.247	0.052	0.128	-0.090	-0.141
4	TTR-Suture	PFI	10	CFD	mm	8.273	8.205	8.334	8.055	8.026	7.989
4	TTR-Suture	PFI	10	HAA	deg	1.677	1.480	1.537	1.600	1.692	0.845
4	TTR-Suture	PFI	10	SVA	deg	16.985	16.802	16.698	17.025	17.266	16.178
4	TTR-Suture	PFI	700	TAS	mm	0.113	0.455	0.011	0.478	0.374	0.383
4	TTR-Suture	PFI	700	CFD	mm	8.908	8.743	8.697	8.737	8.947	8.856
4	TTR-Suture	PFI	700	HAA	deg	1.203	1.310	0.693	1.650	1.512	0.674
4	TTR-Suture	PFI	700	SVA	deg	16.323	16.879	15.495	15.972	16.069	15.844
4	TTR-Suture	DFI	10	TAS	mm	-0.144	0.386	0.279	0.193	0.325	0.514
4	TTR-Suture	DFI	10	CFD	mm	7.825	8.444	8.044	8.269	8.445	8.267
4	TTR-Suture	DFI	10	HAA	deg	1.646	2.118	2.083	2.232	2.463	1.932
4	TTR-Suture	DFI	10	SVA	deg	16.992	17.087	16.586	17.202	17.218	16.910
4	TTR-Suture	DFI	700	TAS	mm	-0.534	0.237	-0.500	-0.454	-0.461	-0.379
4	TTR-Suture	DFI	700	CFD	mm	8.588	8.647	8.271	8.729	8.935	8.725
4	TTR-Suture	DFI	700	HAA	deg	1.470	2.443	1.909	1.667	2.478	2.118
4	TTR-Suture	DFI	700	SVA	deg	16.751	17.056	16.515	16.716	17.334	16.960
5	Intact-Native	N	10	TAS	mm	-1.926	-2.081	-2.288	-1.977	-2.303	-1.928
5	Intact-Native	N	10	CFD	mm	6.598	6.552	6.598	6.697	6.246	6.779
5	Intact-Native	N	10	HAA	deg	-2.531	-2.818	-2.905	-2.976	-3.089	-2.988
5	Intact-Native	N	10	SVA	deg	11.459	11.507	11.773	12.011	11.593	11.914
5	Intact-Native	N	10	FAO	percent	4.927	4.922	4.955	5.009	4.944	4.998
5	Intact-Native	N	700	TAS	mm	-1.684	-1.563	-1.787	-1.743	-1.952	-1.679
5	Intact-Native	N	700	CFD	mm	6.358	5.749	6.256	5.896	5.772	6.255
5	Intact-Native	N	700	HAA	deg	-3.008	-3.159	-3.443	-2.968	-3.290	-3.229
5	Intact-Native	N	700	SVA	deg	10.948	10.419	10.339	10.646	10.428	10.752
5	Intact-Native	N	700	FAO	percent	5.183	5.229	5.199	5.210	5.143	5.108
5	Intact-Native	PF	10	TAS	mm	-2.396	-2.893	-2.288	-2.032	-2.444	-1.700
5	Intact-Native	PF	10	CFD	mm	6.709	6.565	6.615	6.893	6.678	7.061
5	Intact-Native	PF	10	HAA	deg	-3.118	-3.829	-3.411	-2.880	-3.181	-2.917
5	Intact-Native	PF	10	SVA	deg	11.181	11.103	11.777	11.418	11.003	11.825
5	Intact-Native	PF	700	TAS	mm	-1.836	-1.945	-1.704	-1.744	-2.226	-1.821
5	Intact-Native	PF	700	CFD	mm	6.761	6.345	6.701	6.549	6.432	6.787
5	Intact-Native	PF	700	HAA	deg	-2.714	-3.201	-2.775	-3.057	-3.214	-3.628
5	Intact-Native	PF	700	SVA	deg	11.671	11.288	12.031	11.877	11.747	12.165

5	Intact-Native	DF	10	TAS	mm	-1.768	-2.005	-1.832	-2.101	-1.945	-1.914
5	Intact-Native	DF	10	CFD	mm	6.422	6.253	6.242	6.624	6.341	6.665
5	Intact-Native	DF	10	HAA	deg	-2.394	-2.434	-2.569	-2.573	-2.312	-2.486
5	Intact-Native	DF	10	SVA	deg	11.973	12.143	12.066	12.488	11.971	12.368
5	Intact-Native	DF	700	TAS	mm	-1.520	-1.271	-1.414	-1.477	-1.056	-1.693
5	Intact-Native	DF	700	CFD	mm	5.957	6.226	6.126	6.160	6.115	6.114
5	Intact-Native	DF	700	HAA	deg	-2.391	-2.349	-2.446	-2.323	-1.715	-2.655
5	Intact-Native	DF	700	SVA	deg	12.912	13.002	13.029	12.963	12.711	12.502
5	Intact-Native	EV	10	TAS	mm	-2.965	-3.381	-3.603	-3.216	-3.500	-2.801
5	Intact-Native	EV	10	CFD	mm	5.037	4.971	4.730	4.790	5.113	5.133
5	Intact-Native	EV	10	HAA	deg	-5.050	-5.677	-5.774	-5.924	-6.551	-5.791
5	Intact-Native	EV	10	SVA	deg	9.345	9.674	9.512	9.558	9.223	9.611
5	Intact-Native	EV	700	TAS	mm	-3.793	-3.942	-3.829	-3.750	-3.608	-3.269
5	Intact-Native	EV	700	CFD	mm	4.377	4.286	4.789	4.563	4.598	4.813
5	Intact-Native	EV	700	HAA	deg	-5.803	-6.018	-6.048	-6.756	-6.338	-6.085
5	Intact-Native	EV	700	SVA	deg	8.848	9.256	8.672	8.844	8.930	8.847
5	Intact-Native	IN	10	TAS	mm	-0.640	-0.896	-0.987	-0.743	-0.849	-0.628
5	Intact-Native	IN	10	CFD	mm	8.316	7.964	7.968	8.069	8.008	8.236
5	Intact-Native	IN	10	HAA	deg	0.474	0.573	0.249	0.373	0.173	0.422
5	Intact-Native	IN	10	SVA	deg	14.574	14.302	14.382	14.450	14.043	14.010
5	Intact-Native	IN	700	TAS	mm	-0.751	-0.861	-1.059	-0.622	-1.007	-0.712
5	Intact-Native	IN	700	CFD	mm	7.599	7.916	7.974	7.641	7.498	8.081
5	Intact-Native	IN	700	HAA	deg	0.151	-0.254	-0.113	0.031	0.111	0.225
5	Intact-Native	IN	700	SVA	deg	14.623	14.055	13.884	14.499	13.462	14.533
5	Intact-Native	PFE	10	TAS	mm	-3.494	-3.268	-3.428	-3.165	-3.160	-3.290
5	Intact-Native	PFE	10	CFD	mm	5.028	5.056	5.191	4.915	5.242	5.091
5	Intact-Native	PFE	10	HAA	deg	-6.390	-6.174	-6.185	-5.936	-6.040	-5.926
5	Intact-Native	PFE	10	SVA	deg	9.186	9.475	9.528	9.119	8.722	8.884
5	Intact-Native	PFE	700	TAS	mm	-3.245	-2.983	-3.288	-2.998	-3.059	-3.026
5	Intact-Native	PFE	700	CFD	mm	4.761	4.922	4.789	5.070	4.968	4.758
5	Intact-Native	PFE	700	HAA	deg	-6.224	-6.360	-5.921	-5.802	-6.472	-6.019
5	Intact-Native	PFE	700	SVA	deg	8.574	8.467	8.831	8.753	9.043	9.046
5	Intact-Native	DFE	10	TAS	mm	-2.511	-2.872	-2.844	-3.219	-2.816	-3.197
5	Intact-Native	DFE	10	CFD	mm	4.673	4.496	4.545	5.220	4.726	5.224
5	Intact-Native	DFE	10	HAA	deg	-5.470	-5.912	-5.472	-5.795	-5.303	-5.969
5	Intact-Native	DFE	10	SVA	deg	10.076	9.624	9.592	9.408	9.940	9.506
5	Intact-Native	DFE	700	TAS	mm	-2.267	-2.303	-2.704	-2.570	-2.381	-2.319
5	Intact-Native	DFE	700	CFD	mm	4.295	4.688	4.657	5.090	4.626	4.802
5	Intact-Native	DFE	700	HAA	deg	-6.407	-6.529	-6.597	-6.637	-6.225	-6.356
5	Intact-Native	DFE	700	SVA	deg	9.069	9.100	8.682	9.083	9.318	9.271
5	Intact-Native	PFI	10	TAS	mm	-0.775	-0.894	-0.914	-0.915	-0.951	-1.116
5	Intact-Native	PFI	10	CFD	mm	8.464	7.980	7.783	7.917	7.640	7.746
5	Intact-Native	PFI	10	HAA	deg	0.277	0.310	0.053	0.180	-0.173	-0.162
5	Intact-Native	PFI	10	SVA	deg	15.000	14.295	14.144	14.478	14.748	14.032
5	Intact-Native	PFI	700	TAS	mm	-0.380	-0.824	-0.395	-0.765	-0.622	-0.788
5	Intact-Native	PFI	700	CFD	mm	8.270	8.298	8.422	8.349	8.231	8.162
5	Intact-Native	PFI	700	HAA	deg	0.132	-0.312	-0.137	0.047	-0.491	-0.166
5	Intact-Native	PFI	700	SVA	deg	13.979	13.433	13.306	13.679	13.431	13.674
5	Intact-Native	DFI	10	TAS	mm	-0.539	-0.487	-0.827	-0.555	-0.440	-0.134
5	Intact-Native	DFI	10	CFD	mm	8.224	8.275	8.450	8.375	8.001	8.730
5	Intact-Native	DFI	10	HAA	deg	0.797	1.170	-0.068	0.242	0.630	0.974
5	Intact-Native	DFI	10	SVA	deg	14.704	14.409	14.182	14.542	14.785	15.280
5	Intact-Native	DFI	700	TAS	mm	0.202	0.306	0.041	0.072	0.270	0.517
5	Intact-Native	DFI	700	CFD	mm	8.472	8.854	8.963	9.127	8.738	9.361
5	Intact-Native	DFI	700	HAA	deg	1.350	1.484	0.915	0.371	1.048	0.919
5	Intact-Native	DFI	700	SVA	deg	15.645	15.075	15.402	14.741	15.274	15.557
5	TTR-Alone	N	10	TAS	mm	-1.436	-1.724	-1.929	-1.547	-1.394	-1.345
5	TTR-Alone	N	10	CFD	mm	6.428	6.298	6.518	6.248	6.096	6.567
5	TTR-Alone	N	10	HAA	deg	-2.156	-2.093	-2.042	-2.076	-2.756	-2.465
5	TTR-Alone	N	10	SVA	deg	12.225	12.226	12.287	12.154	12.511	12.406
5	TTR-Alone	N	10	FAO	percent	10.420	10.368	10.276	10.383	10.320	10.233
5	TTR-Alone	N	700	TAS	mm	-0.624	-0.675	-1.084	-1.304	-1.059	-0.791
5	TTR-Alone	N	700	CFD	mm	6.021	6.145	5.928	6.180	5.998	6.391
5	TTR-Alone	N	700	HAA	deg	-1.905	-1.879	-1.953	-2.037	-2.199	-2.050
5	TTR-Alone	N	700	SVA	deg	12.192	11.714	12.241	12.243	12.439	12.159
5	TTR-Alone	N	700	FAO	percent	10.555	10.563	10.578	10.482	10.503	10.638
5	TTR-Alone	PF	10	TAS	mm	-2.244	-1.879	-2.019	-1.574	-2.056	-1.716
5	TTR-Alone	PF	10	CFD	mm	6.395	6.559	6.536	6.696	6.452	6.237
5	TTR-Alone	PF	10	HAA	deg	-3.118	-2.599	-3.259	-2.435	-2.668	-2.416
5	TTR-Alone	PF	10	SVA	deg	11.668	11.546	11.404	11.830	11.674	12.213

5	TTR-Alone	PF	700	TAS	mm	-1.253	-1.090	-1.140	-1.195	-1.488	-0.884
5	TTR-Alone	PF	700	CFD	mm	5.782	5.723	5.982	5.700	5.861	6.203
5	TTR-Alone	PF	700	HAA	deg	-2.704	-2.515	-2.560	-2.359	-2.479	-2.210
5	TTR-Alone	PF	700	SVA	deg	12.078	12.146	12.232	11.853	12.236	12.591
5	TTR-Alone	DF	10	TAS	mm	-1.666	-1.717	-1.715	-1.600	-1.587	-1.572
5	TTR-Alone	DF	10	CFD	mm	6.209	6.164	6.101	6.278	6.074	6.305
5	TTR-Alone	DF	10	HAA	deg	-2.338	-1.741	-1.740	-1.831	-1.641	-1.944
5	TTR-Alone	DF	10	SVA	deg	12.569	12.764	12.227	13.145	12.835	13.112
5	TTR-Alone	DF	700	TAS	mm	-0.673	-0.796	-0.703	-0.616	-1.085	-0.585
5	TTR-Alone	DF	700	CFD	mm	6.313	6.097	5.923	6.356	6.171	6.308
5	TTR-Alone	DF	700	HAA	deg	-1.808	-1.472	-1.664	-1.520	-1.822	-1.675
5	TTR-Alone	DF	700	SVA	deg	12.870	12.847	12.100	13.074	12.501	12.879
5	TTR-Alone	EV	10	TAS	mm	-2.791	-2.732	-2.758	-2.738	-2.933	-3.066
5	TTR-Alone	EV	10	CFD	mm	4.833	4.739	4.733	4.907	5.143	4.815
5	TTR-Alone	EV	10	HAA	deg	-5.128	-5.066	-5.356	-5.739	-5.270	-5.576
5	TTR-Alone	EV	10	SVA	deg	9.658	10.093	9.767	10.411	10.508	10.174
5	TTR-Alone	EV	700	TAS	mm	-2.804	-2.217	-2.606	-2.510	-2.157	-2.559
5	TTR-Alone	EV	700	CFD	mm	3.346	3.738	3.293	3.609	3.634	3.620
5	TTR-Alone	EV	700	HAA	deg	-3.377	-2.549	-2.462	-2.599	-2.586	-2.993
5	TTR-Alone	EV	700	SVA	deg	9.814	10.085	10.321	10.846	10.297	9.796
5	TTR-Alone	IN	10	TAS	mm	-0.425	-0.497	-0.094	-0.860	-0.773	-0.608
5	TTR-Alone	IN	10	CFD	mm	7.894	8.200	8.281	8.163	8.041	7.796
5	TTR-Alone	IN	10	HAA	deg	0.890	0.596	1.672	1.033	0.786	0.903
5	TTR-Alone	IN	10	SVA	deg	15.218	14.302	14.996	14.875	14.486	15.169
5	TTR-Alone	IN	700	TAS	mm	1.328	1.745	1.729	1.379	1.381	1.517
5	TTR-Alone	IN	700	CFD	mm	7.353	7.414	7.704	7.379	7.398	7.501
5	TTR-Alone	IN	700	HAA	deg	2.042	2.294	2.474	1.839	2.033	1.675
5	TTR-Alone	IN	700	SVA	deg	15.507	15.293	15.798	14.758	14.721	15.018
5	TTR-Alone	PFE	10	TAS	mm	-2.627	-2.800	-3.207	-2.894	-2.505	-2.999
5	TTR-Alone	PFE	10	CFD	mm	4.576	4.352	5.233	4.965	4.566	4.632
5	TTR-Alone	PFE	10	HAA	deg	-5.382	-5.731	-5.755	-5.730	-5.248	-6.088
5	TTR-Alone	PFE	10	SVA	deg	9.986	9.734	9.069	10.232	10.082	9.018
5	TTR-Alone	PFE	700	TAS	mm	-2.935	-2.275	-2.912	-2.762	-2.520	-2.468
5	TTR-Alone	PFE	700	CFD	mm	2.933	3.225	3.276	3.106	2.975	3.255
5	TTR-Alone	PFE	700	HAA	deg	-5.588	-4.884	-5.152	-5.191	-5.134	-5.198
5	TTR-Alone	PFE	700	SVA	deg	8.809	8.956	8.571	9.209	8.806	8.114
5	TTR-Alone	DFE	10	TAS	mm	-2.775	-2.741	-2.418	-2.238	-2.290	-2.676
5	TTR-Alone	DFE	10	CFD	mm	4.338	4.447	4.666	4.590	4.690	4.588
5	TTR-Alone	DFE	10	HAA	deg	-5.282	-5.346	-4.740	-5.189	-4.788	-5.645
5	TTR-Alone	DFE	10	SVA	deg	9.989	10.277	10.922	10.374	10.264	10.252
5	TTR-Alone	DFE	700	TAS	mm	-0.999	-1.504	-1.157	-1.082	-0.908	-1.364
5	TTR-Alone	DFE	700	CFD	mm	2.247	2.356	2.652	2.435	2.737	2.486
5	TTR-Alone	DFE	700	HAA	deg	-2.757	-3.516	-3.145	-3.113	-3.026	-3.317
5	TTR-Alone	DFE	700	SVA	deg	10.845	10.697	10.735	11.441	10.974	11.105
5	TTR-Alone	PFI	10	TAS	mm	-0.416	-0.456	-0.514	-0.612	-0.782	-0.804
5	TTR-Alone	PFI	10	CFD	mm	7.461	7.280	7.596	7.821	7.559	7.876
5	TTR-Alone	PFI	10	HAA	deg	0.902	0.851	0.572	0.752	1.057	0.321
5	TTR-Alone	PFI	10	SVA	deg	14.916	14.603	14.976	15.079	15.063	14.346
5	TTR-Alone	PFI	700	TAS	mm	1.856	1.837	2.018	1.876	1.630	1.636
5	TTR-Alone	PFI	700	CFD	mm	7.672	7.617	7.702	8.064	7.585	8.288
5	TTR-Alone	PFI	700	HAA	deg	1.781	1.371	1.929	1.451	1.696	1.460
5	TTR-Alone	PFI	700	SVA	deg	14.998	14.269	14.942	14.795	14.731	14.521
5	TTR-Alone	DFI	10	TAS	mm	0.061	-0.363	-0.232	-0.365	-0.028	-0.451
5	TTR-Alone	DFI	10	CFD	mm	7.866	8.021	8.185	8.211	8.106	7.965
5	TTR-Alone	DFI	10	HAA	deg	1.566	1.084	0.927	1.040	1.561	0.773
5	TTR-Alone	DFI	10	SVA	deg	15.035	15.073	15.117	15.271	15.560	15.106
5	TTR-Alone	DFI	700	TAS	mm	0.609	0.169	0.571	0.368	0.565	0.394
5	TTR-Alone	DFI	700	CFD	mm	8.194	8.133	7.841	7.893	8.369	8.202
5	TTR-Alone	DFI	700	HAA	deg	2.529	2.055	2.353	1.877	2.516	2.546
5	TTR-Alone	DFI	700	SVA	deg	14.742	14.392	14.698	14.327	14.285	14.457
5	TTR-Suture	N	10	TAS	mm	-2.097	-1.818	-1.747	-2.136	-1.730	-1.815
5	TTR-Suture	N	10	CFD	mm	6.509	6.165	6.510	6.339	6.792	6.473
5	TTR-Suture	N	10	HAA	deg	-3.191	-2.685	-2.575	-2.798	-2.663	-2.429
5	TTR-Suture	N	10	SVA	deg	12.045	12.015	11.863	11.876	11.996	12.146
5	TTR-Suture	N	10	FAO	percent	7.238	7.196	7.176	7.186	7.259	7.209
5	TTR-Suture	N	700	TAS	mm	-1.025	-0.870	-1.013	-1.127	-0.838	-0.966
5	TTR-Suture	N	700	CFD	mm	7.049	6.612	6.667	6.391	6.627	6.714
5	TTR-Suture	N	700	HAA	deg	-2.849	-2.379	-2.754	-2.275	-2.481	-2.390
5	TTR-Suture	N	700	SVA	deg	12.759	12.971	12.656	12.473	12.894	13.055
5	TTR-Suture	N	700	FAO	percent	7.322	7.467	7.552	7.333	7.531	7.381

5	TTR-Suture	PF	10	TAS	mm	-2.509	-2.134	-2.450	-1.997	-2.076	-2.352
5	TTR-Suture	PF	10	CFD	mm	6.831	6.678	7.146	7.003	7.013	6.493
5	TTR-Suture	PF	10	HAA	deg	-2.933	-2.529	-2.782	-2.942	-2.774	-3.482
5	TTR-Suture	PF	10	SVA	deg	11.593	11.423	11.950	11.419	12.133	11.676
5	TTR-Suture	PF	700	TAS	mm	-1.955	-1.610	-1.982	-1.570	-2.067	-1.866
5	TTR-Suture	PF	700	CFD	mm	6.430	6.326	6.649	6.574	6.356	6.346
5	TTR-Suture	PF	700	HAA	deg	-2.584	-2.139	-2.723	-2.185	-2.373	-2.786
5	TTR-Suture	PF	700	SVA	deg	12.535	12.444	12.250	12.454	12.725	12.913
5	TTR-Suture	DF	10	TAS	mm	-1.979	-1.886	-2.033	-1.664	-1.696	-1.760
5	TTR-Suture	DF	10	CFD	mm	6.285	6.210	6.081	6.244	6.006	6.261
5	TTR-Suture	DF	10	HAA	deg	-2.312	-2.091	-2.524	-2.458	-1.942	-2.216
5	TTR-Suture	DF	10	SVA	deg	12.298	12.436	12.301	12.288	12.710	12.599
5	TTR-Suture	DF	700	TAS	mm	-0.976	-1.202	-1.276	-1.151	-1.378	-1.345
5	TTR-Suture	DF	700	CFD	mm	6.554	6.703	6.566	6.368	6.423	6.869
5	TTR-Suture	DF	700	HAA	deg	-2.011	-2.111	-2.444	-2.147	-1.915	-1.714
5	TTR-Suture	DF	700	SVA	deg	13.028	12.934	11.967	12.709	13.293	12.731
5	TTR-Suture	EV	10	TAS	mm	-2.738	-3.442	-2.969	-2.938	-2.762	-3.037
5	TTR-Suture	EV	10	CFD	mm	4.706	5.193	5.059	4.753	4.798	4.591
5	TTR-Suture	EV	10	HAA	deg	-5.403	-5.866	-5.085	-5.408	-5.602	-5.707
5	TTR-Suture	EV	10	SVA	deg	10.014	9.353	10.209	9.972	9.907	9.475
5	TTR-Suture	EV	700	TAS	mm	-2.390	-2.953	-2.479	-2.933	-2.283	-2.910
5	TTR-Suture	EV	700	CFD	mm	4.820	4.989	4.946	4.815	5.040	4.695
5	TTR-Suture	EV	700	HAA	deg	-5.087	-5.471	-4.745	-5.311	-4.655	-5.108
5	TTR-Suture	EV	700	SVA	deg	9.952	8.878	10.283	9.541	9.841	9.347
5	TTR-Suture	IN	10	TAS	mm	-0.960	-0.810	-0.334	-0.713	-0.337	-0.700
5	TTR-Suture	IN	10	CFD	mm	7.816	8.100	7.711	7.467	7.647	8.349
5	TTR-Suture	IN	10	HAA	deg	0.892	0.589	0.724	0.194	0.759	0.861
5	TTR-Suture	IN	10	SVA	deg	14.425	14.771	14.591	14.627	14.705	14.192
5	TTR-Suture	IN	700	TAS	mm	-0.122	-0.174	-0.085	0.264	0.510	0.049
5	TTR-Suture	IN	700	CFD	mm	7.457	7.390	7.566	7.521	7.775	7.977
5	TTR-Suture	IN	700	HAA	deg	1.119	0.756	0.947	1.081	1.697	1.328
5	TTR-Suture	IN	700	SVA	deg	14.724	14.851	14.693	15.399	15.535	14.626
5	TTR-Suture	PFE	10	TAS	mm	-3.046	-3.007	-3.334	-3.253	-3.058	-3.095
5	TTR-Suture	PFE	10	CFD	mm	4.901	4.798	4.695	4.689	4.918	5.189
5	TTR-Suture	PFE	10	HAA	deg	-5.723	-5.776	-6.029	-6.016	-5.951	-5.770
5	TTR-Suture	PFE	10	SVA	deg	9.527	9.743	9.679	9.015	9.760	9.548
5	TTR-Suture	PFE	700	TAS	mm	-2.807	-2.835	-2.876	-2.529	-2.750	-2.518
5	TTR-Suture	PFE	700	CFD	mm	4.125	4.055	3.864	3.638	4.139	4.132
5	TTR-Suture	PFE	700	HAA	deg	-6.038	-5.989	-6.369	-6.164	-5.862	-5.935
5	TTR-Suture	PFE	700	SVA	deg	10.131	10.229	11.018	10.271	10.854	10.544
5	TTR-Suture	DFE	10	TAS	mm	-2.992	-2.702	-2.726	-3.107	-3.271	-3.054
5	TTR-Suture	DFE	10	CFD	mm	4.564	4.394	4.539	4.987	4.542	5.059
5	TTR-Suture	DFE	10	HAA	deg	-5.416	-4.569	-5.096	-5.612	-5.525	-5.323
5	TTR-Suture	DFE	10	SVA	deg	10.036	9.983	10.105	9.502	9.657	9.594
5	TTR-Suture	DFE	700	TAS	mm	-2.196	-1.759	-2.001	-1.789	-1.749	-1.784
5	TTR-Suture	DFE	700	CFD	mm	3.219	3.425	3.405	3.415	3.079	3.313
5	TTR-Suture	DFE	700	HAA	deg	-4.513	-4.151	-4.313	-4.101	-4.007	-4.187
5	TTR-Suture	DFE	700	SVA	deg	9.253	9.919	9.611	10.250	10.001	9.975
5	TTR-Suture	PFI	10	TAS	mm	-0.856	-0.444	-0.935	-0.860	-0.465	-0.491
5	TTR-Suture	PFI	10	CFD	mm	7.716	7.723	7.884	8.026	7.797	7.707
5	TTR-Suture	PFI	10	HAA	deg	0.319	0.397	0.486	0.268	0.408	0.315
5	TTR-Suture	PFI	10	SVA	deg	14.708	14.176	14.492	14.242	14.026	14.542
5	TTR-Suture	PFI	700	TAS	mm	0.705	0.775	0.610	0.533	0.413	0.573
5	TTR-Suture	PFI	700	CFD	mm	8.164	7.806	7.805	7.935	8.079	8.226
5	TTR-Suture	PFI	700	HAA	deg	0.213	0.382	-0.584	-0.252	-0.641	-0.523
5	TTR-Suture	PFI	700	SVA	deg	15.729	15.657	15.645	15.166	14.839	15.660
5	TTR-Suture	DFI	10	TAS	mm	-0.307	-0.341	-0.275	-0.290	-0.563	-0.310
5	TTR-Suture	DFI	10	CFD	mm	7.951	8.120	8.035	8.512	8.240	8.407
5	TTR-Suture	DFI	10	HAA	deg	0.836	0.485	1.097	1.234	0.937	0.927
5	TTR-Suture	DFI	10	SVA	deg	15.188	14.543	14.269	14.765	14.306	14.479
5	TTR-Suture	DFI	700	TAS	mm	1.130	0.687	0.965	1.053	1.078	0.873
5	TTR-Suture	DFI	700	CFD	mm	9.145	8.979	8.971	9.161	9.022	9.125
5	TTR-Suture	DFI	700	HAA	deg	1.175	0.658	1.358	1.466	1.368	0.596
5	TTR-Suture	DFI	700	SVA	deg	14.502	14.382	14.226	14.796	14.611	13.950
